# Whole genome sequencing for *USH2A*-associated disease reveals several pathogenic deep-intronic variants that are amenable to splice correction

**DOI:** 10.1016/j.xhgg.2023.100181

**Published:** 2023-01-18

**Authors:** Janine Reurink, Nicole Weisschuh, Alejandro Garanto, Adrian Dockery, L. Ingeborgh van den Born, Isabelle Fajardy, Lonneke Haer-Wigman, Susanne Kohl, Bernd Wissinger, G. Jane Farrar, Tamar Ben-Yosef, Fatma Kivrak Pfiffner, Wolfgang Berger, Marianna E. Weener, Lubica Dudakova, Petra Liskova, Dror Sharon, Manar Salameh, Ashley Offenheim, Elise Heon, Giorgia Girotto, Paolo Gasparini, Anna Morgan, Arthur A. Bergen, Jacoline B. ten Brink, Caroline C.W. Klaver, Lisbeth Tranebjærg, Nanna D. Rendtorff, Sascha Vermeer, Jeroen J. Smits, Ronald J.E. Pennings, Marco Aben, Jaap Oostrik, Galuh D.N. Astuti, Jordi Corominas Galbany, Hester Y. Kroes, Milan Phan, Wendy A.G. van Zelst-Stams, Alberta A.H.J. Thiadens, Joke B.G.M. Verheij, Mary J. van Schooneveld, Suzanne E. de Bruijn, Catherina H.Z. Li, Carel B. Hoyng, Christian Gilissen, Lisenka E.L.M. Vissers, Frans P.M. Cremers, Hannie Kremer, Erwin van Wijk, Susanne Roosing

**Affiliations:** 1 Department of Human Genetics, Radboud University Medical Center, Nijmegen, the Netherlands; 2 Donders Institute for Brain Cognition and Behaviour, Radboud University Medical Center, Nijmegen, the Netherlands; 3 Molecular Genetics Laboratory, Institute for Ophthalmic Research, Centre for Ophthalmology, University of Tübingen, Tübingen, Germany; 4 Department of Pediatrics, Amalia’s Children Hospital, Radboud University Medical Center, Nijmegen, The Netherlands; 5 Radboud Institute of Molecular Life Sciences, Radboud University Medical Center, Nijmegen, the Netherlands; 6 The School of Genetics & Microbiology, Smurfit Institute of Genetics, Trinity College Dublin, Dublin 2, Ireland; 7 The Rotterdam Eye Hospital, Rotterdam, the Netherlands; 8 Centre de Biologie Pathologie Génétique, CHU de Lille, Lille, France; 9 The Rappaport Faculty of Medicine, Technion-Israel Institute of Technology, Haifa, Israel; 10 Institute of Medical Molecular Genetics, University of Zurich, Schlieren, Switzerland; 11 Neuroscience Center Zurich, University and ETH Zurich, Zurich, Switzerland; 12 Center for Integrative Human Physiology, University of Zurich, Zurich, Switzerland; 13 CRO Oftalmic, Moscow, Russia; 14 Department of Paediatrics and Inherited Metabolic Disorders, First Faculty of Medicine, Charles University and General University Hospital in Prague, Prague, Czech Republic; 15 Department of Ophthalmology, First Faculty of Medicine, Charles University and General University Hospital in Prague, Prague, Czech Republic; 16 Division of Ophthalmology, Hadassah University Medical Center, Faculty of Medicine, The Hebrew University of Jerusalem, Jerusalem, Israel; 17 Departments of Ophthalmology and Vision Sciences, The Hospital for Sick Children, The University of Toronto, Toronto, ON, Canada; 18 Institute for Maternal and Child Health—I.R.C.C.S. “Burlo Garofolo”, 34137 Trieste, Italy; 19 Department of Medicine, Surgery and Health Sciences, University of Trieste, 34149 Trieste, Italy; 20 Department of Clinical Genetics, Amsterdam UMC, University of Amsterdam, 1105 Amsterdam, the Netherlands; 21 Department of Ophthalmology, Amsterdam UMC, University of Amsterdam, 1105 Amsterdam, the Netherlands; 22 Department of Ophthalmology, Radboud University Medical Center, Nijmegen, the Netherlands; 23 Department of Ophthalmology, Erasmus Medical Center, Rotterdam, the Netherlands; 24 Department of Epidemiology, Erasmus Medical Center, Rotterdam, the Netherlands; 25 Department of Clinical Genetics, The Kennedy Center, Copenhagen University Hospital, 2600 Glostrup, Denmark; 26 Institute of Clinical Medicine, University of Copenhagen, 2200 Copenhagen, Denmark; 27 Center for Human Genetics, University Hospitals Leuven, Leuven, Belgium; 28 Hearing & Genes, Department of Otorhinolaryngology, Radboud University Medical Center, Nijmegen, the Netherlands; 29 Division Laboratories, Pharmacy and Biomedical Genetics, Department of Genetics, University Medical Center of Utrecht, Utrecht, the Netherlands; 30 Division of Human Genetics, Center for Biomedical Research (CEBIOR), Faculty of Medicine, Diponegoro University, Semarang, Indonesia; 31 Department of Ophthalmology, Rijnstate Hospital, Arnhem, the Netherlands; 32 Department of Medical Genetics, University Medical Center Groningen, University of Groningen, Groningen, the Netherlands

**Keywords:** USH2A, Usher syndrome, retinitis pigmentosa, usherin, whole genome sequencing, minigene splice assay, splicing, antisense oligonucleotides, photoreceptor precursor cells, pseudoexon

## Abstract

A significant number of individuals with a rare disorder such as Usher syndrome (USH) and (non-)syndromic autosomal recessive retinitis pigmentosa (arRP) remain genetically unexplained. Therefore, we assessed subjects suspected of *USH2A*-associated disease and no or mono-allelic *USH2A* variants using whole genome sequencing (WGS) followed by an improved pipeline for variant interpretation to provide a conclusive diagnosis.

One hundred subjects were screened using WGS to identify causative variants in *USH2A* or other USH/arRP-associated genes. In addition to the existing variant interpretation pipeline, a particular focus was put on assessing splice-affecting properties of variants, both *in silico* and *in vitro*. Also structural variants were extensively addressed. For variants resulting in pseudoexon inclusion, we designed and evaluated antisense oligonucleotides (AONs) using minigene splice assays and patient-derived photoreceptor precursor cells.

Biallelic variants were identified in 49 of 100 subjects, including novel splice-affecting variants and structural variants, in *USH2A* or arRP/USH-associated genes. Thirteen variants were shown to affect *USH2A* pre-mRNA splicing, including four deep-intronic *USH2A* variants resulting in pseudoexon inclusion, which could be corrected upon AON treatment.

We have shown that WGS, combined with a thorough variant interpretation pipeline focused on assessing pre-mRNA splicing defects and structural variants, is a powerful method to provide subjects with a rare genetic condition, a (likely) conclusive genetic diagnosis. This is essential for the development of future personalized treatments and for patients to be eligible for such treatments.

## Teaser

We show that whole genome sequencing, combined with thorough variant interpretation focused on assessing pre-mRNA splicing defects and structural variants, is a powerful method to provide individuals with suspected *USH2A*-associated disease a (likely) conclusive genetic diagnosis. In addition, we developed antisense oligonucleotides that effectively redirect aberrant pre-mRNA splicing.

## Introduction

Whole genome sequencing (WGS) is increasingly recognized as a vital technique in (diagnostic) genetic screening of disease, including inherited retinal diseases (IRDs), such as *USH2A*-associated Usher syndrome type 2 (USH2, MIM: #276901) and autosomal recessive retinitis pigmentosa (arRP, MIM: #613809). The added value compared with whole exome sequencing (WES) and most targeted sequencing panels is that it detects (novel) deep-intronic variants,^1^^,^^2^^,^^3^ structural variants (SVs),^4^^,^^5^ and variants in regulatory regions,^6^ resulting in an increased diagnostic yield in IRD cases.^4^^,^^7^^,^^8^

In previous studies, WES and targeted sequencing panels solved 36% to 63% of persons with non-syndromic arRP and 84% to 90% of Usher syndrome (USH) cases.^4^^,^^9^ We hypothesized that a substantial portion of the remaining unsolved cases has an SV or a deleterious deep-intronic variant that potentially results in inclusion of a pseudoexon (PE). To date, 80 (likely) pathogenic SVs (larger than 50 nucleotides) have already been submitted to ClinVar (March 29, 2022) and several deep-intronic variants resulting in PE inclusion have been reported for *USH2A*.^1^^,^^2^^,^^3^ Therefore, it is plausible that novel SVs and deep-intronic variants that cause PE inclusion will be identified when genetically unexplained cases are screened with WGS.

The *USH2A* gene is one of the most frequently mutated IRD-associated genes, implicated in both USH2 and arRP, as pathogenic variants are identified in 57% to 79% and 12% to 25% of affected individuals, respectively.^10^ *USH2A*-associated IRD is still considered untreatable. However, several gene-specific treatment options are currently in different stages of (pre-)clinical development, including splice-modulation using antisense oligonucleotides (AONs),^11^ CRISPR-Cas9-based gene correction,^12^ and exon-excision.^13^ *Ultevursen*, an AON that induces the skipping of *USH2A* exon 13, is currently being evaluated in a phase II/III clinical trial (ClinicalTrials.gov Identifier: NCT05158296). These developments further stress the importance for subjects to obtain a conclusive genetic diagnosis in order to be eligible for future personalized therapies.

In this study, we aimed to identify missing variants in *USH2A* in a cohort of 100 largely mono-allelic arRP and USH2 subjects through WGS, assess an effect on splicing of all detected single nucleotide variants (SNVs), and to develop an AON-based splice correction strategy for specific deep-intronic variants. In addition, our approach could serve as a future framework for detection and treatment of variants in genes associated with other hereditary disorders.

## Material and methods

### Ethics declaration

Written informed consent was obtained by the corresponding centers, adherent to the tenets of the declaration of Helsinki and as approved by the local ethics committee of the Radboud University Medical Center Nijmegen, as an amendment to the approval by the local ethics committee of the Rotterdam Eye Hospital (MEC-2010-359; OZR protocol no. 2009-32).

### Whole genome sequencing

DNA of unrelated probands was collected from subjects from the Netherlands, Germany, Ireland, Northern Ireland, Israel, Russia, Czech Republic, Switzerland, France, Italy, Denmark, Canada, and Belgium. Subjects were included if they were clinically diagnosed with arRP (n = 47), USH2 (n = 49), or autosomal recessive hearing impairment (DFNB, n = 3) in those who were still in the first or second decade of life. One subject with cone-rod dystrophy (CRD) and hearing loss was included. In addition, we required that pre-screening of the (majority of) *USH2A* exons was performed and only one (potentially or likely) pathogenic variant in *USH2A* was identified or no *USH2A* variants were identified. In 67 subjects, a (potentially or likely) pathogenic variant in *USH2A* was reported prior to WGS screening (34 for RP, 30 for USH, and three for DFNB). *USH2A* variants that were identified in probands prior to their inclusion in our WGS analysis pipeline are shown in Table 1 and screening methods that were used prior to WGS are listed in Table S1.

**Table 1:** An overview of all samples including identified potentially pathogenic *USH2A* variants

Study ID	Causal gene	Variant 1 cDNA	Protein effect variant 1	Variant 1 ACMG classification	Variant 2 cDNA	Protein effect variant 2	Variant 2 ACMG classification	Status of proband
**Solved and possibly solved**								

arRP1	*USH2A*	c.2299del	p.(Glu767Serfs^∗^21)	Pathogenic	c.14134-5T>C	p.[=,Val4712Profs^∗^2]	VUS	Possibly solved
arRP4^14^	*PQLC2*	c.596G>A	p.(Arg199Gln)	Likely pathogenic	c.596G>A	p.(Arg199Gln)	Likely pathogenic	Solved
arRP6	*EYS*	c.2527G>A	p.(Gly843Arg)	Likely pathogenic	c.-12233_-447-18280del	p.(?)	VUS	Possibly solved
arRP8	*USH2A*	c.11864G>A	p.(Trp3955^∗^)	Pathogenic	c.9949C>T	p.(Arg3317Cys)	VUS	Possibly solved
arRP10	*USH2A*	c.10817T>C	p.(Leu3606Pro)	VUS	c.5573-19A>G	p.Gly1858_Thr1925del	Likely pathogenic	Possibly solved
arRP11	*USH2A*	c.1679del	p.(Pro560Leufs^∗^31)	Pathogenic	c.9371+1G>C	p.(?)	Pathogenic	Solved
arRP19	*USH2A*	c.8559-2A>G	p.(?)	Pathogenic	c.13335_13343del	p.(Glu4445_Met4447del)	Likely pathogenic	Solved
arRP20	*USH2A*	c.12574C>T	p.(Arg4192Cys)	Likely pathogenic	c.4627+25436_4987+659del	p.(?)	Likely pathogenic	Solved
arRP21	*USH2A*	c.5573-1G>T	p.(?)	Likely pathogenic	c.2303G>A	p.[Cys768Tyr,Cys766Tyrfs^∗^3,Glu767_Gly937del]	VUS	Possibly solved
arRP22	*USH2A*	c.10712C>T	p.(Thr3571Met)	Likely pathogenic	c.12575G>A	p.(Arg4192His)	Pathogenic	Solved
arRP24	*USH2A*	c.11105G>A	p.(Trp3702^∗^)	Pathogenic	c.9433C>T	p.(Leu3145Phe)	VUS	Possibly solved
arRP26	*USH2A*	c.14803C>T	p.(Arg4935^∗^)	Pathogenic	c.12575G>A	p.(Arg4192His)	Pathogenic	Solved
arRP29	*PROM1*	c.1301+2T>C	p.(?)	Pathogenic	c.2131-695A>C	p.(?)	VUS	Possibly solved
arRP34^6^	*USH2A*	c.2276G>T	p.(Cys759Phe)	Pathogenic	c.2276G>T	p.(Cys759Phe)	Pathogenic	Solved
arRP36	*USH2A*	c.2276G>T	p.(Cys759Phe)	Pathogenic	c.4397-3890A>G	p.Ala1465_Ala1466ins^∗^5	Likely pathogenic	Solvedˆ
arRP37^15^	*RPE65*	c.886dup	p.(Arg296Lysfs^∗^7)	Pathogenic	c.675C>A	p.(Asp215Valfs^∗^4)	Pathogenic	Solvedˆ
arRP41^6^	*USH2A*	c.2276G>T	p.(Cys759Phe)	Pathogenic	c.2276G>T	p.(Cys759Phe)	Pathogenic	Solved
arRP42^6^	*USH2A*	c.2276G>T	p.(Cys759Phe)	Pathogenic	c.2276G>T	p.(Cys759Phe)	Pathogenic	Solved
DFNB2	*USH2A*	c.653T>A	p.(Val218Glu)	Pathogenic	c.4627+25436_4987+659del	p.(?)	Likely pathogenic	Solvedˆ
USH5	*USH2A*	c.1267G>T	p.(Gly423^∗^)	Likely pathogenic	c.14791+5G>T	p.Tyr4862Alafs^∗^22	Likely pathogenic	Solved
USH7	*USH2A*	c.13245_13246del	p.(Gly4416Valfs^∗^2)	Likely pathogenic	c.652-23899_2809+1417dup	p.(?)	Pathogenic	Solved
USH8	*USH2A*	c.1823G>A	p.(Cys608Tyr)	Likely pathogenic	c.8655_8681+1681del	p.(?)	Likely pathogenic	Solved
USH10	*USH2A*	c.12067-2A>G	p.(?)	Pathogenic	c.4628-22994_2652del	p.(?)	Pathogenic	Solved
USH11	*PEX6*	c.2245G>A	p.(Gly749Ser)	Likely pathogenic	c.1802G>A	p.(Arg601Gln)	Likely pathogenic	Solvedˆ
USH12	*USH2A*	c.2299del	p.(Glu767Serfs^∗^21)	Pathogenic	c.9259-9T>A	p.Val3087Phefs^∗^4	Likely pathogenic	Solvedˆ
USH13	*USH2A*	c.11864G>A	p.(Trp3955^∗^)	Pathogenic	c.5775A>T	p.Gly1858_Thr1925del	Likely pathogenic	Solvedˆ
USH16	*USH2A*	c.7244C>G	p.(Ser2415^∗^)	Pathogenic	c.7595-2144A>G	p.Lys2532Thrfs^∗^56	Pathogenic	Solved
USH17	*USH2A*	c.2299del	p.(Glu767Serfs^∗^21)	Pathogenic	c.15063_15081delinsGC	p.(Thr5022Glnfs^∗^150)	Pathogenic	Solved
USH19	*USH2A*	c.8740C>T	p.(Arg2914^∗^)	Pathogenic	c.9371+1G>C	p.(?)	Pathogenic	Solved
USH20^16^	*ARSG*	c.588C>A	p.(Tyr196^∗^)	Pathogenic	c.705-3940_ 982+2952del	p.(Ser235Argfs^∗^29)	Pathogenic	Solvedˆ
USH22	*USH2A*	c.3187_3188del	p.(Gln1063Serfs^∗^15)	Pathogenic	c.1841-2A>G	p.(?)	Pathogenic	Solved
USH23	*USH2A*	c.802G>C	p.(Gly268Arg)	Likely pathogenic	c.14583-26A>G	p.[=,Tyr4862Alafs^∗^22]	VUS	Possibly solved
USH24	*USH2A*	c.15089C>A	p.(Ser5030^∗^)	Pathogenic	c.5573-834A>G	p.(?)	Likely pathogenic	Solved
USH25	*MYO7A*	c.2282+1G>A	p.(?)	Likely pathogenic	c.5482_5485del	p.(Tyr1828Alafs^∗^50)	Likely pathogenic	Solved
USH26	*USH2A*	c.11864G>A	p.(Trp3955^∗^)	Pathogenic	c.9258+1261_9371+1513del	p.(?)	Pathogenic	Solved
USH28	*USH2A*	c.2299del	p.(Glu767Serfs^∗^21)	Pathogenic	c.3251_4627+31194del	p.(?)	Likely pathogenic	Solved
USH29	*USH2A*	c.2610C>A	p.(Cys870^∗^)	Pathogenic	c.4133T>C	p.(Leu1378Pro)	VUS	Possibly solved
USH31	*USH2A*	c.11549-1G>A	p.(?)	Pathogenic	c.2994-3030_14343+488dup	p.(?)	Pathogenic	Solved
USH32	*USH2A*	c.14408T>C	p.(Ile4803Thr)	VUS	c.4885+375A>G^15^	p.Ser1629Valfs^∗^52	Likely pathogenic	Possibly solved
USH33	*USH2A*	c.14791+2T>C	p.(?)	Likely pathogenic	c.9335_9371+8063delinsGAA GACACTCC	p.(?)	Pathogenic	Solved
USH34	*USH2A*	c.10561T>C	p.(Trp3521Arg)	Pathogenic	c.785-6636_1840+208del	p.(?)	Likely pathogenic	Solved
USH35^16^	*ARSG*	c.1326del	p.(Ser443Alafs^∗^12)	Pathogenic	c.1024C>T	p.(Arg342Trp)	Likely pathogenic	Solved
USH37	*USH2A*	c.2299del	p.(Glu767Serfs^∗^21)	Pathogenic	c.9258G>T	p.[Arg3037_Val3087del,Val3049^∗^]	Likely pathogenic	Solvedˆ
USH39	*USH2A*	c.2299del	p.(Glu767Serfs^∗^21)	Pathogenic	c.2311G>T	p.(Glu771^∗^)	Likely pathogenic	Solvedˆ
USH41	*USH2A*	c.14525C>A	p.(Ser4842^∗^)	Likely pathogenic	c.4397-3890A>G	p.Ala1465_Ala1466ins^∗^5	Likely pathogenic	Solvedˆ
USH42^17^	*USH2A*	c.2299del	p.(Glu767Serfs^∗^21)	Pathogenic	g.209815568_215637482inv	p.(?)	Pathogenic	Solved
USH44^17^	*USH2A*	c.9258+2601_9371+1539del	p.(?)	Pathogenic	g.42320825_215677220delins42320846_215677215inv	p.(?)	Pathogenic	Solvedˆ
USH46	*USH2A*	c.9959-3C>G	p.[Met3321Asnfs^∗^22,=,Gly3320_Ser3338del]	VUS	c.1551-504C>T	p.Arg517_Cys518ins^∗^13	Likely pathogenic	Possibly solvedˆ
USH49	*USH2A*	c.2299del	p.(Glu767Serfs^∗^21)	Pathogenic	c.1841-377A>G	p.[Thr613_Gly614ins^∗^9, = ]	VUS	Possibly solved

**Unsolved**								

arRP2		–	–	–	–	–	–	Unsolved
arRP3		c.2299del	p.(Glu767Serfs^∗^21)	Pathogenic	–	–	–	Unsolved
arRP5		c.15433G>A	p.(Val5145Ile)	Benign	–	–	–	Unsolved
arRP7		c.10931C>T	p.(Thr3644Met)	VUS	c.9975G>A	p.(Gly3325=)	VUS	Unsolved
arRP9		c.6240G>T	p.(Lys2080Asn)	Benign	–	–	–	Unsolved
arRP12		c.[2276G>T(;)4618G>A]	p.(Cys759Phe(;)Asp1540Asn)	Pathogenic	**-**	**-**		Unsolved
arRP13		c.3443C>T	p.(Pro1148Leu)	VUS	–	–	–	Unsolved
arRP14		c.2276G>T	p.(Cys759Phe)	Pathogenic	c.11687T>C	p.(Ile3896Thr)	VUS	Unsolved
arRP15		c.11864G>A	p.(Trp3955^∗^)	Pathogenic	–	–	–	Unsolved
arRP16		c.4486C>A	p.(Pro1496Thr)	VUS	c.14664G>A	p.(Thr4888=)	Likely benign	Unsolved
arRP17		c.7939C>T	p.(Pro2647Ser)	VUS	–	–	–	Unsolved
arRP18		c.3309C>A	p.(Tyr1103^∗^)	Pathogenic	–	–	–	Unsolved
arRP23		c.12295-3T>A	p.Thr4099Valfs^∗^2	Pathogenic	–	–	–	Unsolved
arRP25		c.920_923dup	p.(His308Glnfs^∗^16)	Pathogenic	c.6240G>T	p.(Lys2080Asn)	Benign	Unsolved
arRP27		c.2276G>T	p.(Cys759Phe)	Pathogenic	–	–	–	Unsolved
arRP28		c.3664G>A	p.(Ala1222Thr)	VUS	–	–	–	Unsolved
arRP30		c.6001C>T	p.(Arg2001Cys)	Benign	–	–	–	Unsolved
arRP31		c.1439T>C	p.(Val480Ala)	VUS	–	–	–	Unsolved
arRP32		c.4732C>T	p.(Arg1578Cys)	Likely pathogenic	–	–	–	Unsolved
arRP33		c.14333C>A; 15377T>C	p.(Ala4778Asp;Ile5126Thr)	Benign	–	–	–	Unsolved
arRP35		c.5858C>G	p.(Ala1953Gly)	VUS	–	–	–	Unsolved
arRP38		c.12343C>T	p.(Arg4115Cys)	VUS	–	–	–	Unsolved
arRP39		c.2522C>A	p.(Ser841Tyr)	Benign	–	–	–	Unsolved
arRP40		–	–	–	–	–	–	Unsolved
arRP43		c.9258G>T	p.[Arg3037_Val3087del,Val3049^∗^]	Likely pathogenic	–	–	–	Unsolved
arRP44		c.11597C>T	p.(Ala3866Val)	Likely benign	–	–	–	Unsolved
arRP45		–	–	–	–	–	–	Unsolved
arRP46		–	–	–	–	–	–	Unsolved
arRP47		c.8710G>A	p.(Val2904lle)	VUS	–	–	–	Unsolved
CRD1		–	–	–	–	–	–	Unsolved
DFNB1		c.11864G>A	p.(Trp3955^∗^)	Pathogenic	–	–	–	Unsolved
DFNB3		c.11927C>T	p.(Thr3976Met)	VUS	–	–	–	Unsolved
USH1		c.14020A>G	p.(Arg4674Gly)	Likely pathogenic	–	–	–	Unsolved
USH2		c.2920G>A	p.(Asp974Asn)	Likely benign	–	–	–	Unsolved
USH3		c.7594+250G>T	p.(?)	Benign	–	–	–	Unsolved
USH4		c.5780A>G	p.(Tyr1927Cys)	VUS	–	–	–	Unsolved
USH6		c.6670G>T	p.(Gly2224Cys)	VUS	c.1644+7453A>G	p.=	VUS	Unsolved
USH9		c.8167C>T	p.(Arg2723^∗^)	Pathogenic	–	–	–	Unsolved
USH14		**-**	**-**	–	–	–	–	Unsolved
USH15		c.15433G>A	p.(Val5145Ile)	Benign	c.14753C>T	p.(Thr4918Met)	Likely benign	Unsolved
USH18		–	–	–	–	–	–	Unsolved
USH21		–	–	–	–	–	–	Unsolved
USH27		c.1522G>A	p.(Ala508Thr)	VUS	–	–	–	Unsolved
USH30		c.2299del	p.(Glu767Serfs^∗^21)	Pathogenic	–	–	–	Unsolved
USH36		c.11864G>A	p.(Trp3955^∗^)	Pathogenic	–	–	–	Unsolved
USH38		–	–	–	–	–	–	Unsolved
USH40		c.5516T>A	p.(Val1839Glu)	VUS	–	–	–	Unsolved
USH43		–	–	–	–	–	–	Unsolved
USH45		–	–	–	–	–	–	Unsolved
USH47		c.1439T>C	p.(Val480Ala)	VUS	–	–	–	Unsolved
USH48		–	–	–	–	–	–	Unsolved

Underlined variants are newly identified variants with WGS in this study. Probands for which segregation analysis confirmed the variants to be *in trans* are marked with an ˆ in the 'status of the proband'-column*.* Reference sequences (hg19):, *ARSG:* NM_014960.5, *EYS:* NM_001142800.2*,MYO7A:* NM_000260.4, *PEX6:* NM_000287.4*, PQLC2*: NM_001040125.2*, PROM1:* NM_006017.3, *USH2A*: NM_206933.4. arRP, autosomal recessive retinitis pigmentosa; USH, Usher syndrome; VUS, variant of uncertain significance. All variants are *USH2A* variants, unless another causal gene is listed.

WGS was performed as previously described.^8^^,^^18^ Variants in the entire *USH2A* gene (chr1:215796236–216596738), 10 previously determined potential regulatory regions of *USH2A*,^6^ the known retinal modifier of *USH2A*-associated disease *PDZD7,*^19^ and variants in the entire genomic regions of other genes associated with USH (n = 17, Table S2; RetNet; visited May 21, 2021) and arRP (n = 62, Table S2) were extracted and evaluated. Variants with a gnomAD allele frequency (AF) ≤ 1% in the general population and in any subpopulation were prioritized. SNVs were considered potentially pathogenic if they were (1) a stop gain variant, frameshift variant, in-frame insertion, or deletion or a canonical splice site variant; (2) a missense variant with a Grantham score ≥80 (range: 5–215), CADD_PHRED ≥15 (range: 0–99) or PhyloP ≥2.7; or (3) a putative splice-modulating variant with two out of four SpliceAI Δ scores ≥0.1 or one SpliceAI Δ score ≥0.15 (default settings, range 0–1).^20^ Copy number variants (CNVs) were called with control-FREEC and SVs were called with Manta Structural Variant Caller V.1.1.0.^21^^,^^22^ CNVs and SVs were prioritized if they passed the internal quality filter as previously described^8^; had a high quality score (≥100 of 1,000); had an AF ≤ 1% in databases, such as gnomAD, Decipher, and GoNL; and affected at least one exon of *USH2A* or the aforementioned 62 arRP-associated or 17 USH-associated genes. Segregation analysis was performed if DNA samples of relatives were available.

### Minigene splice assays

Variants predicted to have an effect on splicing (two out of four SpliceAI Δ scores ≥0.1 or one SpliceAI Δ score ≥0.15) were selected for a minigene splice assay. Four variants were selected prior to the release of SpliceAI and did not meet these criteria. Two of these variants (c.1644+7453A>G and c.4396+6885T>C) were included because an effect on pre-mRNA splicing was predicted by splice prediction tools incorporated in Alamut Visual Plus. Variant c.8710G>A was included due to its 1 base pair downstream location of c.8709C>T that was shown to have an effect on pre-mRNA splicing in a previous study,^9^ whereas a fourth variant (c.6806-7599C>G) was selected as a unique variant absent from gnomAD in one of the first samples that was analyzed with WGS. Selected variants were either deep-intronic variants, non-canonical splice site variants, missense variants, or variants that affected a “branchpoint A” upstream of an exon. Constructs were generated with Gateway cloning technology (Thermo Fisher Scientific, Carlsbad, CA, USA) and minigene splice assays were performed as published previously.^23^ Primer sequences are listed in Table S3.

### Variant classification

SNVs were classified according to the American College of Medical Genetics (ACMG) guidelines with Franklin and SVs were manually classified according to published ACMG guidelines.^24^^,^^25^ SNVs with an effect on pre-mRNA splicing were classified based on their effect in the minigene splice assay; variants with a full stop gain effect in all transcripts were classified as pathogenic, variants resulting in an in-frame deletion thereby disrupting a predicted functional protein domain were classified as likely pathogenic and variants with remaining conventionally spliced transcript were classified as variant of uncertain significance (VUS). Subjects with two (likely) pathogenic variants were considered genetically solved, subjects with one (likely) pathogenic variant and one VUS were considered “possibly solved.”

### Antisense oligonucleotides

AONs were designed according to previously published guidelines^26^^,^^27^ and purchased from Eurogentec (Seraing, Belgium) with 2′-O-MOE modifications and a complete phosphorothiorate backbone. For each PE, at least two AONs were designed complementary to splice acceptor or donor sites, or exonic splice enhancer regions. Only one AON targeting the PE resulting from c.4885+375A>G could be designed due to low GC-content of the PE sequence. NCBI blast searches were performed to exclude potential off-target binding sites for each designed AON. Moreover, a scrambled oligonucleotide (SON) as well as an AON containing three mismatches compared with the most potent AON for each target (3ntMM AON) were designed and purchased. All AONs were dissolved in sterile phosphate buffered saline (PBS 1x) to a stock concentration of 1 mM and diluted in PBS to concentrations of 0.01 mM, 0.02 mM, 0.05 mM, and 0.1 mM. One microliter of each AON was co-transfected in duplicate with individual minigene splice assay plasmids containing the variant of interest, as described in the minigene splices assays section. Transfected cells were subjected to RNA analyses as described before.^23^

### Generation of photoreceptor precursor cells

Blood was obtained from subjects arRP36 (heterozygous for c.4397-3890A>G), USH46 (heterozygous for c.1551-504C>T), and a healthy control. Peripheral blood mononuclear cells were isolated and reprogrammed into induced pluripotent stem cells (iPSCs) through episomal nucleofection, as described previously,^28^ and subsequently differentiated into photoreceptor precursor cells (PPCs).^17^^,^^29^ iPSC lines were generated at the Stem Cell Technology Center of the Radboudumc (Nijmegen, the Netherlands). AONs or a SON were gymnotically delivered to the PPCs on day 28 by adding them directly to the culture medium. Cycloheximide (CHX, in a final concentration of 100 μg/mL) was added on day 29 and cells were harvested in sterile PBS on day 30. Levels of PE inclusion and reversal after treatment were determined with PCR and RT-qPCR (in duplicate) according to standard protocols (primer sequences in Table S4). AON efficacy was assessed based on the splicing correction capacity at the different AON concentrations.

## Results

### Forty-one probands were (likely) solved having biallelic *USH2A* variants

One hundred unrelated subjects were included for WGS analysis. Forty-seven of these subjects were clinically diagnosed with non-syndromic arRP, 49 with USH, three with DFNB, and one with CRD and hearing loss. The subjects with DFNB had one (likely) pathogenic *USH2A* variant and were still in the first or second decade of life, potentially not (yet) presenting early signs of vision loss.

In total, 6,054 unique SNVs were identified within *USH2A* and predicted regulatory elements. Of these, 2,820 variants had a gnomAD AF ≤1% in the general population. Ninety-seven variants were considered potentially pathogenic, as they met our inclusion criteria for being a stop gain variant, frameshift variant, in-frame insertion or deletion, a canonical splice site variant, or a potentially pathogenic missense variant or putative splice-modulating variant (Table S5). Nineteen of these SNVs were not reported in ClinVar or the Leiden Open (source) Variation Database (LOVD) and were therefore considered novel variants in our study. Twelve *USH2A* SVs on 13 alleles adhered to our criteria, including two duplications, two inversions,^16^ and eight deletions of which one (c.4627+25436_4987+659del) was identified in two probands. The largest SV with both breakpoints within *USH2A* was a duplication from exon 15 to 65 (c.2994-3030_14343+488dup), the shortest SV was a deletion of exon 43 (c.8655_8681+1681del). All SVs were confirmed with PCR and subsequent Sanger sequencing. After assessment of all individual variants from the total cohort, 41 cases were genetically explained with (likely) biallelic *USH2A* variants. An overview of all variants that were classified as (likely) pathogenic or were VUS that were deemed potentially causative is presented in Table 1.

**Figure 1:**
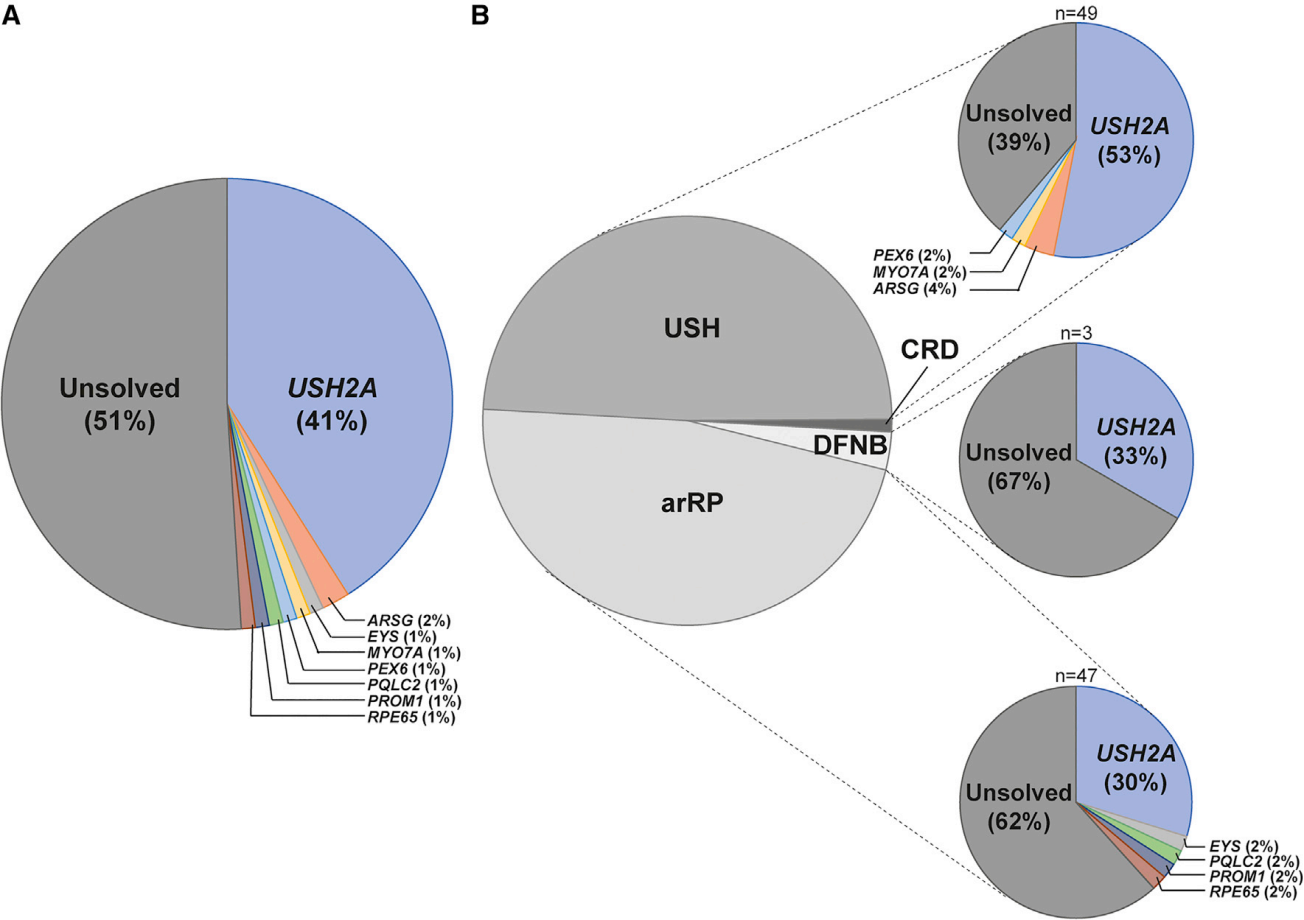
Overview of causative genes in a cohort of 100 USH and arRP-affected individuals (A) In 49 of the 100 probands, (likely) biallelic (likely) pathogenic variants were identified in genes associated with autosomal recessive retinitis pigmentosa (arRP) or Usher syndrome (USH). (B) The solved rate per phenotype (arRP, deafness [DFNB] or USH) is shown. The majority (61%) of USH subjects are genetically explained, compared with 38% of arRP subjects and one out of three DFNB affected individuals. The remaining subject (in dark gray) was diagnosed with cone-rod dystrophy (CRD) with hearing loss and remained unsolved.

All samples were also evaluated for the presence of potentially pathogenic variants in other arRP- or USH-associated genes (Table S2), applying the same criteria to detect biallelic variants in these genes. We identified 133 unique variants in these genes, resulting in eight of the remaining probands being (possibly) solved with biallelic variants in the following genes: *ARSG* (two cases, described in Velde et al.^14^)*, EYS, MYO7A, PEX6, PQCL2* (described in Millo et al.^15^), *PROM1,* and *RPE65* (described in Panneman et al.^30^)*.* An overview of solve rates is visualized in Figure 1.

### Assessment of the effect of variants on splicing using minigene splice assays

**Figure 2:**
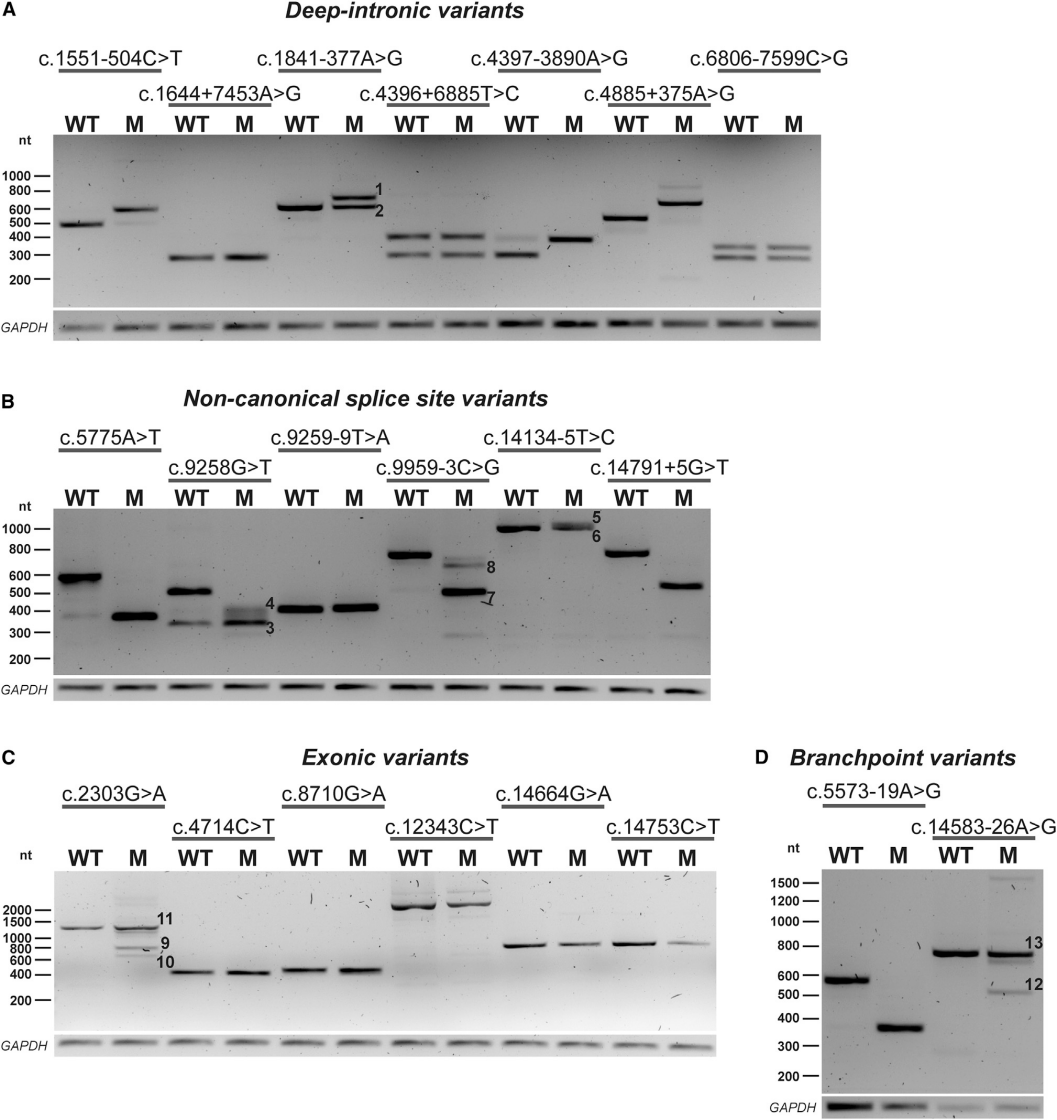
Minigene splice assays for 21 variants with a potential effect on splicing Representative gel images from minigene splice assays for deep-intronic variants, non-canonical splice site variants, exonic variants, and branchpoint variants. The numbers visualized on the gel correspond to the protein effects described below. (A) Three deep-intronic variants (c.1551-504C>T, c.4397-3890A>G, and c.4885+375A>G^32^) resulted in PE inclusion in all amplified transcripts. Variant c.1841-377A>G, resulted in the inclusion of a 94 nt PE (1) and remaining conventionally spliced transcript (2). (B) Two non-canonical splice site variants (c.5775A>T and c.14791+5G>T) resulted in the complete skipping of exon 28 or exon 67 in all detected transcripts, whereas variant c.9259-9T>A resulted in a 7-nt extension. Variant c.9258G>T was shown to have a dual effect: partial skipping of exon 46 (3) and alternative partial skipping of exon 46 (4). Likewise, variant c.14134-5T>C resulted in a 47-nt extension upstream of exon 65 (5) and remaining conventionally spliced transcript (6). Variant c.9959-3C>G induced two alternative pre-mRNA splicing events: skipping of complete exon 51 (7) and partial skipping of exon 51 (8) with incomplete penetrance. (C) Variant c.2303G>A had multiple effects, with part of the transcripts lacking the center region of exon 13 (9), part lacking the 3′ side of exon 13 (10) and part being conventionally spliced mRNA (11). (D) Variant c.5573-19A>G resulted in skipping of exon 28 in all transcripts, whereas variant c.14583-26A>G resulted in skipping of exon 67 (12) and conventionally spliced mRNA (13). Transfections were performed in duplicate, *GAPDH* was used as loading control in all experiments. An overview of the observed effects on pre-mRNA splicing and the consequences on protein level is listed in Table S7. M, variant of interest; nt, nucleotides; WT, wild type.

We identified 36 variants with two out of four SpliceAI Δ scores ≥0.1 or one SpliceAI Δ score ≥0.15 (Table S6).^20^ Stop gain variants (n = 2) and canonical splice site variants (n = 7) were excluded from these analyses as they are generally considered to be pathogenic. For three variants (c.5573-834A>G, c.7595-2144A>G, and c.12295-3T>A) a deleterious effect on *USH2A* pre-mRNA splicing was already previously determined^1^^,^^2^^,^^31^ and they were therefore omitted for testing. We also excluded one variant that was an artifact and confirmed to be absent with Sanger sequencing (c.1551-508C>T), one variant in a proband that would remain mono-allelic after performance of a minigene splice assay (c.4732C>T), and one variant for which gain of canonical splice sites was predicted (c.9975G>A) (Table S6). Minigene splice assays were performed for the remaining 21 variants (Figures 2A–2D, Table S7). Thirteen of these variants showed a(n) (partial) effect on splicing (Figure S1), of which eight variants affected pre-mRNA splicing in all amplified transcripts and these were therefore classified as (likely) pathogenic. The remaining five variants (c.1841-377A>G, c.2303G>A, c.9959-3C>G, c.14134-5T>C, and c.14583-26A>G) only revealed a partial effect on splicing and were therefore classified as VUS.

(a) Deep-intronic variants (n = 7)

Seven of the 21 variants that were evaluated in a minigene splice assay were deep-intronic *USH2A* variants (Figure 2A). Four of seven tested deep-intronic variants were shown to have an effect on pre-mRNA splicing. Variant c.1551-504C>T induced the inclusion of a 118 nt PE (PE8; chr1:216495824–216495941) in intron 8, resulting in premature termination of protein translation (p.Arg517_Cys518ins^∗^13). Variant c.4397-3890A>G resulted in the inclusion of an 87 nt PE (PE20; chr1:216352719–216352805) in intron 20, containing an in-frame stop codon (p.Ala1465_Ala1466ins∗5). Variant c.4885+375A>G was already recently described.^32^ We confirmed inclusion of a 130 nt PE (PE23; chr1:216261985–216262114) in intron 23 (p.Ser1629Valfs∗52). A deep-intronic variant located in intron 10, c.1841-377A>G (in individual USH50), resulted in the inclusion of a 94 nt PE in part of the transcripts (PE10; chr1:216463130–216463223) (p.[Thr613_Gly614ins∗9,=]). Variants c.1644+7453A>G, c.4396+6885T>C and c.6806-7599C>G (identified *in cis* with c.2276G>T) did not reveal an effect on pre-mRNA splicing.

(b) Variants in non-canonical splice site region (n = 6)

Six *USH2A* non-canonical splice site variants were identified (Figure 2B). Two variants resulted in the complete skipping of either exon 28 (c.5775A>T; p.Gly1858_Thr1925del) or exon 67 (c.14791+5G>T; p.Tyr4862Alafs^∗^22) in all detected transcripts, whereas variant c.9259-9T>A resulted in a 7-nt extension upstream of exon 47 (p.Val3087Phefs^∗^4). Variant c.9258G>T was shown to have a dual effect; partial skipping of exon 46 (Δ153 nt) in part of the transcripts combined with an alternative partial skipping of exon 46 (Δ116 nt) in the remaining transcripts, as a consequence of the use of another alternative splice donor site in exon 46 (p.[Arg3037_Val3087del,Val3049^∗^]). Likewise, variant c.14134-5T>C resulted in a 47-nt extension upstream of exon 65 in part of the transcripts (p.[=,Val4712Profs^∗^2]). Variant c.9959-3C>G induced multiple alternative pre-mRNA splicing events; skipping of complete exon 51 and partial skipping of exon 51 (Δ57 nt) (p.[Met3321Asnfs^∗^22,=,Gly3320_Ser3338del]), with incomplete penetrance.

(c) Exonic variants (n = 6)

The potential effect on splicing of six exonic variants outside of canonical splice site regions was evaluated (Figure 2C). Variant c.2303G>A (p.(Cys768Tyr)) had multiple effects on pre-mRNA splicing, with part of the transcripts lacking the center region of exon 13, leaving the canonical splice sites intact and with use of a novel donor splice site 129 nt downstream of the canonical splice acceptor site and a novel splice acceptor site 115 nt upstream of the canonical splice donor site. Other transcripts lacked 513 nt at the 3′ side of exon 13 or were conventionally spliced mRNA (p.[Cys766Tyrfs^∗^3,Glu767_Gly937del,Cys768Tyr]). The other five exonic variants did not show an effect on pre-mRNA splicing using the corresponding minigene splice assays, i.e., c.4714C>T (*in cis* with c.2299del), c.8710G>A, c.12343C>T, c.14664G>A, and c.14753C>T.

(d) Branchpoint variants (n = 2)

Two variants (c.5573-19A>G, c.14583-26A>G, Figure 2D) were predicted to affect the adenine in a branch sequence (“branchpoint A”) upstream of exons 28 and 67, respectively. A minigene splice assay revealed that variant c.5573-19A>G resulted in skipping of exon 28 (p.Gly1858_Thr1925del) in all transcripts, while variant c.14583-26A>G resulted in skipping of exon 67 and conventionally spliced mRNA (p.[=,Tyr4862Alafs^∗^22]).

### Design and evaluation of antisense oligonucleotides to correct aberrant pre-mRNA splicing events

AONs and controls were designed and evaluated for their potential to correct aberrant splicing induced by four deep-intronic variants resulting in PE inclusion (c.1551-504C>T, c.1841-377A>G, c.4397-3890A>G, and c.4885+375A>G). Characteristics of all AONs and their position relative to the targeted PE and exonic splice enhancers are described in Table S8 and Figure S2.

**Figure 3:**
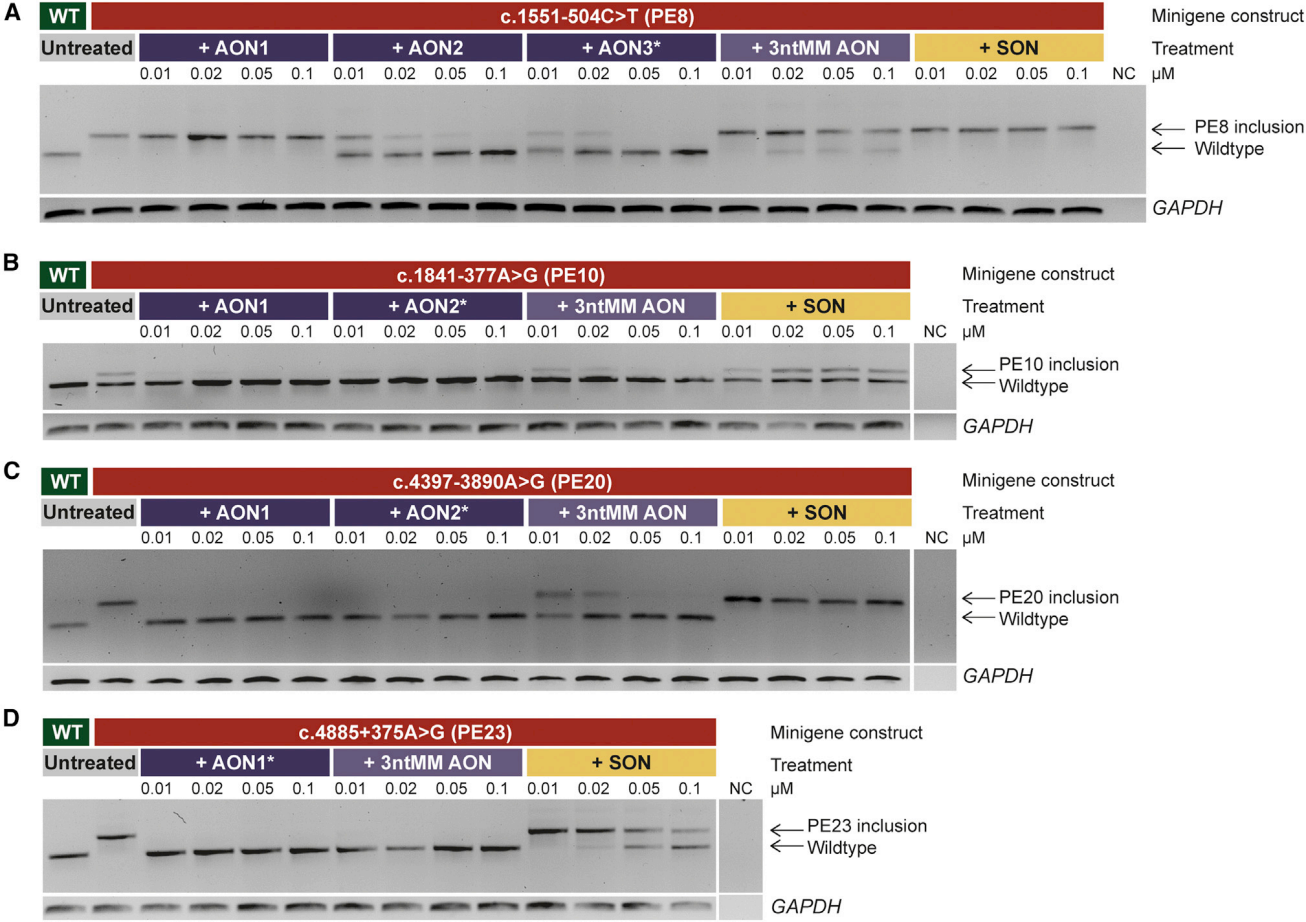
Antisense oligonucleotide (AON) treatment dilution series for four deep-intronic targets Representative gel images from AON treatment studies in minigenes. (A) Two out of three AONs (AON2 and AON3) were effective in reverting a 118-nt pseudoexon (PE) inclusion in intron 8, caused by variant c.1551-504C>T, for all tested concentrations. A three-nucleotide mismatch antisense oligonucleotide (3ntMM AON) of the most potent AON (AON3, marked with an asterisk) and a scrambled oligonucleotide (SON), had minimal to no effect on reverting PE inclusion. (B) Variant c.1841-377A>G resulted in partial inclusion of a 94-nt PE in intron 10. Both AONs efficiently reverted PE inclusion, while a 3ntMM AON of AON2 had reduced effect and an SON was not effective. (C) Variant c.4397-3890A>G caused inclusion of an 87-nt PE in intron 20. Both AONs efficiently reverted PE inclusion, while a 3ntMM AON of AON2 had reduced effect and an SON was not effective. (D) Variant c.4885+375A>G causes inclusion of a 130-nt PE in intron 23. The AON that could be designed for the region was efficient in reverting PE inclusion. A 3ntMM AON was fully effective as well and an SON had reduced effect. NC, negative control; nt, nucleotides; WT, wild type. Transfections were performed in duplicate, *GAPDH* was used as loading control in all experiments.

For all four targets, at least one of the AONs was capable of redirecting aberrant splicing at the lowest concentration (0.01 μM) using the matching minigene splice assay vectors (Figure 3). Transfection with an SON did not show effect for three of the four targets, which indicates that the splicing correction is sequence-specific. To further confirm the sequence-specificity, 3ntMM AONs were also assessed for their potential to redirect splicing in the corresponding minigene splice assays. For c.1551-504C>T and c.4397-3890A>G, these 3ntMM AONs were indeed less effective than the original AONs, even at the highest concentration used (0.1 μM), corroborating the effect of the original AON to be sequence-specific. In contrast, no difference in efficiency of splicing redirection was observed between the original and 3ntMM AONs against c.1841-377A>G (PE10) and c.4885+375A>G (PE23) at all tested concentrations. The SON of PE23 also redirected splicing. Therefore, it is unclear whether or not the effect of the designed AON to correct aberrant PE23 splicing is sequence-specific.

### AONs are effective in patient-derived PPCs

PPCs were generated from iPSCs derived from two subjects (arRP36 and USH46) who were confirmed compound heterozygous for c.4397-3890A>G (PE20) and c.2276G>T (p.(Cys759Phe)) and c.1551-504C>T (PE8) and c.9959-3C>G (p.[Met3321Asnfs^∗^22,=,Gly3320_Ser3338del]), respectively. These PPCs enabled us to confirm the PE inclusion caused by these variants and to test the AONs in a cellular model resembling human photoreceptors within the entire genetic context of the patient. RT-qPCR analyses of several neuronal progenitor, photoreceptor, and retinal pigment epithelium markers indicated that the differentiation of iPSCs into PPCs was successful for both cell lines (Figure S3).

**Figure 4:**
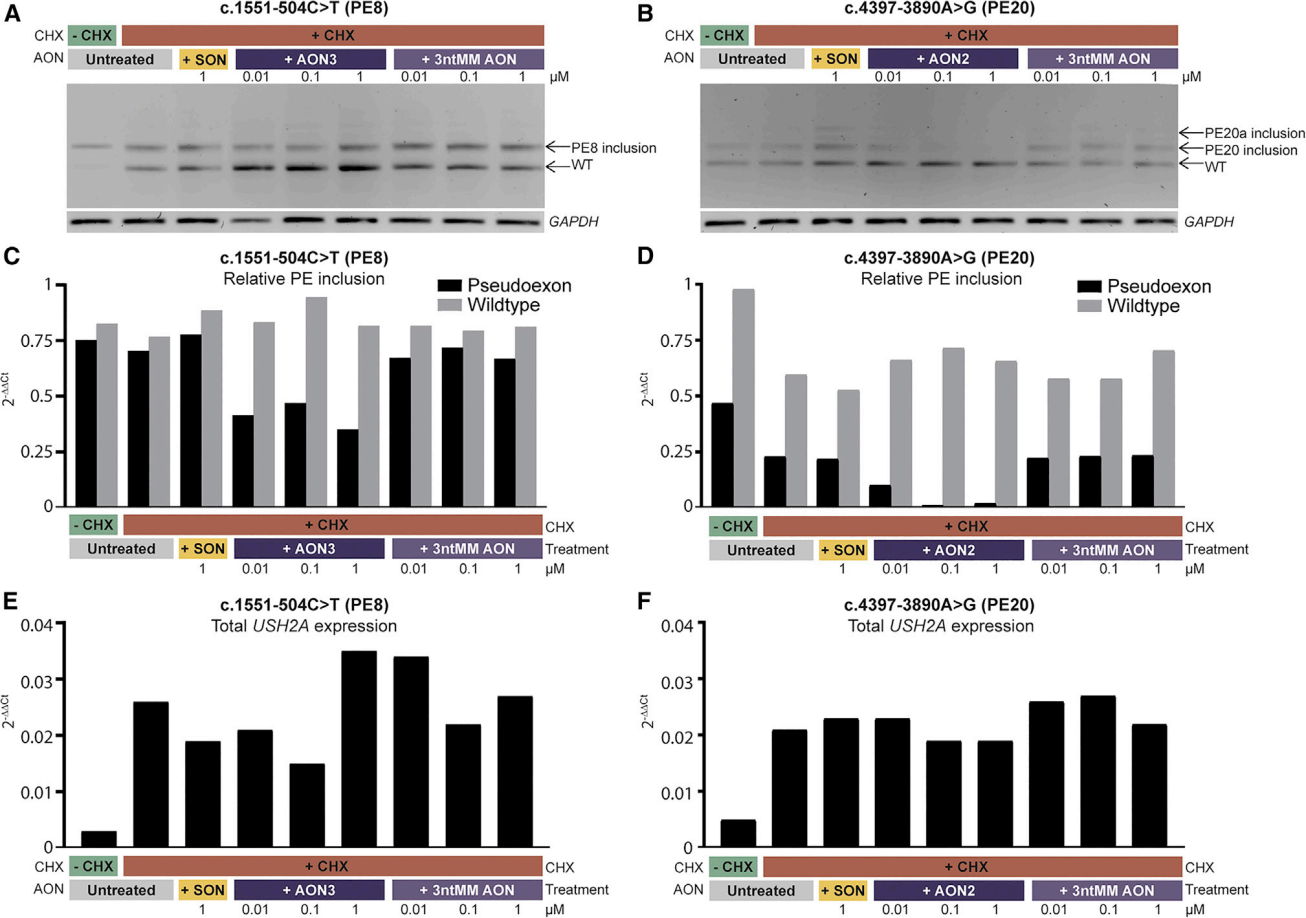
AON treatment of photoreceptor precursor cells (PPCs) differentiated from patient-derived induced pluripotent stem cells (iPSCs) (A) RT-PCR on mRNA of PPC samples that were heterozygous for c.1551-504C>T and subsequent gel electrophoresis showed that ∼50% of transcripts harbor a pseudoexon (PE), while 50% are wild-type (WT) transcripts. Treatment with three different concentrations of antisense oligonucleotide 3 (AON3) against PE8 for 48 h resulted in a reduction, but not complete reversion of PE inclusion. Treatment with a scrambled oligonucleotide (SON) or 3-nucleotide mismatch AON (3ntMM AON) did not result in reduction of PE inclusion. *GAPDH* was used as loading control. (B) In PPCs heterozygous for c.4397-3890A>G, inclusion of PE20 was fully reverted after treatment with AON2 against PE20, while an SON and 3ntMM AON did not have an effect. (C and D) The effect observed with RT-PCR was confirmed with RT-qPCR. Levels of transcripts containing the PE of interest as well as WT transcript was compared with *USH2A* exon 13 expression (normalized against *GUSB*) and confirmed that AON3 against PE8 can partially rescue PE8 inclusion, while AON2 against PE20 has a full effect. (E and F) Expression of *USH2A* (exon 13) compared with *GUSB. USH2A* expression was relatively low in all samples; however, lowest in samples not treated with cycloheximide (CHX).

As expected, PE inclusion was observed in PPCs derived from subjects having the c.1551-504C>T (PE8) or c.4397-3890A>G (PE20) variant (Figure 4). For variant c.4397-3890A>G, AON2 was effective in redirecting aberrant splicing at all three tested concentrations, while the 3ntMM AON and SON did not show any effect. This effect was confirmed with RT-qPCR as a reduction of 49% (after treatment with 0.01 μM AON) to 92% (treated with 0.1 μM AON) of transcripts containing PE20 as compared with the control sample (treated with CHX, but not with AON) was observed. Interestingly, inclusion of an additional PE (PE20a) was observed in the untreated samples and samples that were treated with 3ntMM AON and SON, but not in the samples treated with AON2. This PE20a is possibly a result of variant c.4396+7263del and is assumed to be part of the c.4397-3890A>G haplotype because it was detected with WGS in both subjects having the c.4397-3890A>G variant.

For variant c.1551-504C>T, AON3 redirected aberrant *USH2A* pre-mRNA splicing, albeit with a lower efficiency than was observed in the minigene splice assay, as still remaining PE8-containing transcripts could be detected after AON treatment in all tested concentrations. Compared with the amount of transcript that contains PE8 in the control sample that was not treated with AON, a reduction of 33% (treated with 0.1 μM AON) to 50% (treated with 1 μM AON) of PE8-containing transcript was observed.

## Discussion

Through our study, 49 out of 100 (49%) previously unsolved arRP and (suspected) USH individuals now received a conclusive or possibly conclusive genetic diagnosis. Our analysis confirmed that WGS is a powerful method for solving cases that remained genetically unsolved after screening the *USH2A* exons and flanking intronic regions. Using the combination of WGS and subsequent dedicated minigene splice assays, we identified novel pathogenic deep-intronic variants and SVs and confirmed a splice defect for other exonic, branchpoint and non-canonical splice site variants. WGS followed by testing of splice defects should therefore be considered as a valuable method to increase genetic solve rates, which is especially important in the current era of upcoming genetic therapies and increased focus on personalized medicine. Furthermore, we showed that AONs are a valuable tool to correct aberrant splicing as a consequence of deep-intronic variants in *USH2A.*

To compare the efficacy of our WGS analysis with published WGS studies is complex, due to varying pre-screening conditions and inclusion criteria. However, the solve rate in our study seems in line with other WGS studies that include *USH2A* variants*.* A WGS study from the United Kingdom solved 31% of persons with IRDs that did not receive a complete genetic diagnosis after WES and 55% of cases that were directly screened with WGS.^4^ In another study in which 57 mono-allelic IRD cases from the Netherlands, Ireland, and Israel were included that were previously screened with WES or target-capture sequencing, the solve rate was 21% (12 of 57).^8^ In our study, we solved 49% of cases that underwent prior testing using WES or targeted sequencing, such as molecular inversion probe-based sequencing. We identified several variants that are not (easily) being detected by WES or targeted sequencing, such as pathogenic deep-intronic *USH2A* variants (in seven cases) or SVs (13 alleles in 12 cases). SVs have been previously shown to explain a portion of missing heritability in cases that were screened with targeted sequencing panels.^33^ The detection of 13 (likely) pathogenic *USH2A* SVs in our cohort of 100 subjects, representing 11% of (likely) pathogenic variants identified within *USH2A*, emphasized the value of WGS compared with targeted sequencing panels and WES for a substantial increase in diagnostic yield for IRDs.

Of the solved probands, 22 subjects had SNVs that could have been detected through WES or targeted sequencing. This was likely to be partially caused by lack of detection due to incomplete screening with targeted sequencing. However, nine variants resulted in deviations in pre-mRNA splicing in a minigene splice assay and were located either in an exon or in a non-canonical splice site region. Previous reports estimated that up to a third of disease-causing variants disrupt pre-mRNA splicing, either directly or through mis-regulation of splice-regulating factors.^34^ Our results emphasize that increased knowledge on variant interpretation and functional testing of possible effects on splicing of branchpoint variants, missense variants, synonymous variants, and non-canonical splice site variants will increase diagnostic yield in *USH2A* screening, and in genetic testing for hereditary disorders in general. Re-evaluation of targeted sequencing and WES data of currently still unsolved cases with a particular focus on splice-modulating properties is therefore recommended.

The genetic diagnosis for 13 subjects could be completed after performing a minigene splice assay for variants that were selected based on our criteria for SpliceAI. Eight of these variants displayed an effect on all detected transcripts in the corresponding minigene splice assays (Figure S1 and Table S7). To our knowledge, the c.5573-19A>G variant, reported previously as VUS in both LOVD and ClinVar, is one of the first suggested branchpoint variants within *USH2A* resulting in a pathogenic effect.^35^ The identification of an additional potential branchpoint variant c.14583-26A>G (p.[=,Tyr4862Alafs^∗^22]) stresses the importance of including the evaluation of branchpoint variants in genetic screening efforts. Five variants that were tested in a minigene splice assay showed residual conventionally spliced transcripts along with a deleterious effect on splicing and they were therefore classified as VUS.

Recent studies have been published on specificity and sensitivity of splice prediction tools.^36^^,^^37^ In a study by Rowlands et al.,^36^ SpliceAI (suggested Δ score ≥0.2) outperformed other splice prediction tools, although false positive and false negative calls were observed. In general, our findings are in line with the findings of Rowlands et al. that SpliceAI is able to adequately predict effects on pre-mRNA splicing. Only four of the 17 variants (24%) that met our inclusion criteria (two out of four SpliceAI Δ scores ≥0.1 or one Δ score ≥0.15) did not display an effect on pre-mRNA splicing in HEK293T cells, which were all exonic variants. Four other variants, including one exonic variant, that were tested in this study did not meet our SpliceAI inclusion criteria, but for none of these variants an effect on pre-mRNA splicing was observed. The inclusion criteria in our study were less restrictive compared with those used by Rowlands et al. and Riepe et al. (≥0.2),^36^^,^^37^ yielding three additional variants (c.1841-377A>G, c.5573-19A>G, and c.5775A>T) with a maximum SpliceAI Δ score between 0.1 and 0.2, which did show an effect on pre-mRNA splicing. We would therefore suggest using 0.1 as a criterion for SpliceAI for deep-intronic, non-canonical splice site and branchpoint variants, but this could be adapted for exonic variants as no effect on pre-mRNA splicing was observed for the majority of exonic variants that we tested. A SpliceAI criterion of ≥0.2 for exonic variants outside the non-canonical splice site regions would exclude all exonic variants that did not have an effect on pre-mRNA splicing in our minigene splice assays but would yield the remaining variant (c.2303G>A) that did have an effect. The use of other splice prediction tools in addition to SpliceAI could also be considered for this type of variants.

Screening of variants with a potential effect on pre-mRNA splicing in a broader genetic context, such as larger minigenes or use of patient-derived PPCs, may result in alternative outcomes. In addition, previous research has shown that recognition of splice sites can be cell type specific,^38^ and the splicing patterns observed in our minigene splice assay may deviate from the natural situation in the human retina and inner ear. However, these variants have effect on *USH2A* pre-mRNA splicing and were all identified in arRP and USH subjects with a previously identified mono-allelic pathogenic *USH2A* variant, suggesting that additional functional evidence may determine that these variants are indeed (likely) pathogenic according to ACMG guidelines. Furthermore, similar studies performed for another IRD gene, *ABCA4*, have shown that variants creating new splice sites are recapitulated in patient-derived cells, and only those creating splicing enhancers may show tissue-dependent specificity.^38^ Another important point is that little is known about the minimal amount of remaining wild-type transcript that would be required to retain normal retinal function. Functional data derived from the in-frame skipping of zebrafish *ush2a* exon 13 suggests that as little as ∼20% of functional transcripts could be enough to retain retinal function.^11^ Future research in cellular and animal models will shed light on this question, potentially resulting in the reclassification of variants with an incomplete effect on splicing.

We were able to obtain patient material from two individuals carrying deep-intronic variants that was subsequently used to generate iPSCs to differentiate them to PPCs. Using these cells we could validate our findings in the minigene system. Unexpectedly, we identified an additional PE (PE20a; chr1:216356236–216356337), incorporated in the mRNA derived from cultured PPCs of subject arRP36 with variant c.4397-3890A>G (PE20). PE20a was only identified in samples that were treated with CHX, and treatment with AON against PE20 also appeared to revert inclusion of PE20a. The presence of an additional PE could be an artifact of our assay; however, this stresses the importance of considering an entire haplotype when designing a therapeutic strategy and the importance of using large sequence parts flanking the variant of interest, preferably including flanking exons, in dedicated minigene splice assays.

We also identified variants and splicing defects in *USH2A* exon 13. Currently a phase II/III clinical trial to induce exon 13 skipping is ongoing. Therefore, this information is very valuable for patients, as they might be eligible to receive future treatments. Although mutations in exon 13 are relatively recurrent, this is also important for individuals with ultra-rare or even unique pathogenic variants. The impact of Milasen, an AON customized for a single subject suffering from Batten disease, has led to (inter)national initiatives to develop treatments for those ultra-rare cases with no commercial interest.^39^ Highlighting once again the importance of having a concise genetic diagnosis in order to become eligible for future therapeutic genetic interventions.

Unfortunately, we were unable to provide 51 of 100 probands a solid genetic diagnosis. In most of these cases, one (likely) pathogenic *USH2A* variant is present, and therefore another pathogenic *USH2A* variant might well reside *in trans*. However, in the general population, approximately 36% of individuals are healthy carriers of a pathogenic variant in an IRD-associated gene.^40^ While we screened for causative coding and non-coding variants in other genes associated with arRP or USH, by chance, subjects in our cohort may have a pathogenic variant in *USH2A*, with the underlying genetic cause of their disease elsewhere. In fact, eight cases in our cohort were solved after identification of biallelic variants in IRD genes other than *USH2A*. Furthermore, SVs could remain undetected or unrecognized, due to the nature of short-read sequencing techniques.^16^^,^^41^^,^^42^ Pathogenic variants may also reside in low coverage regions, in genes associated with disorders that are phenotypically overlapping, or in genes not yet associated with IRDs. We did not observe potentially pathogenic variants in 10 potential regulatory regions of the *USH2A* gene^6^ nor in the known retinal disease modifier *PDZD7.*^19^ Improved knowledge on *USH2A* regulatory elements*,* potential modifiers, being either protective or negative, or potentially digenic inheritance patterns is likely to result in more precise classification of variants in the future.

In conclusion, we confirmed that our strategy, with elaborate WGS screening and thorough variant interpretation and thorough assessment of splice defects associated with all types of SNVs, is an effective method to significantly improve diagnostic solve rates. As a result of our strategy, we (possibly) solved 49% (49 of 100) of genetically unexplained probands with suspected *USH2A*-associated disease. In 21 subjects, we identified (likely) pathogenic variants in *USH2A* exon 13 of whom 14 subjects received a definite genetic diagnosis and these are now considered eligible for receiving *Ultevursen*/QR-421a-mediated exon 13 skipping therapy when available.^11^ Moreover, we identified three novel pathogenic deep-intronic variants that result in the inclusion of a PE and we were able to develop effective AONs that redirected aberrant pre-mRNA splicing. Collectively, our experimental approach has been effective in the identification of novel, potentially treatable, disease-causing variants in *USH2A* and can be applied to the diagnostic pipelines for all inherited disorders known so far.

## Data Availability

Data are available upon request. Pathogenic variant data are uploaded to the Leiden Open (source) Variation Database (LOVD: https://databases.lovd.nl/shared/genes/USH2A). All other whole genome sequencing data are subject to controlled access because these may compromise research participant privacy. These data may become available upon a data transfer agreement approved by the local ethics committee and can be obtained from corresponding author S.R. upon reasonable request.

## Web resources

ClinVar: https://www.ncbi.nlm.nih.gov/clinvar/

DECIPHER: https://www.deciphergenomics.org/

Franklin: https://franklin.genoox.com/clinical-db/home

gnomAD SV: https://gnomad.broadinstitute.org/gene/ENSG00000042781?dataset=gnomad_sv_r2_1

gnomAD SNV: https://gnomad.broadinstitute.org/gene/ENSG00000042781?dataset=gnomad_r3

GoNL: https://www.nlgenome.nl/

LOVD: https://databases.lovd.nl/shared/genes/USH2A

RetNet: https://sph.uth.edu/retnet/disease.htm

## Supplemental information

Document S1. Figures S1–S3 and Tables S1–S4, S7, and S8

Table S5. All USH2A variants that met our variant inclusion criteria in 100 cases

Table S6. All USH2A variants with a predicted effect on splicing

Document S2. Article plus supplemental information
