# Supplementary material for: Whole genome sequencing for USH2A-associated disease reveals several pathogenic deep-intronic variants that are amenable to splice correction
Source: HGG Adv. 2023 Jan 18;4(2):100181. doi: 10.1016/j.xhgg.2023.100181 (PMC9918427; doi:10.1016/j.xhgg.2023.100181)
Supplement: Document S1. Figures S1–S3 and Tables S1–S4, S7, and S8 [file mmc1.pdf]

HGGA, Volume *4*

Supplemental information

Whole genome sequencing for *USH2A*-associated disease reveals several pathogenic deep-intronic variants that are amenable to splice correction

Janine Reurink, Nicole Weisschuh, Alejandro Garanto, Adrian Dockery, L. Ingeborgh van den Born, Isabelle Fajardy, Lonneke Haer-Wigman, Susanne Kohl, Bernd Wissinger, G. Jane Farrar, Tamar Ben-Yosef, Fatma Kivrak Pfiffner, Wolfgang Berger, Marianna E. Weener, Lubica Dudakova, Petra Liskova, Dror Sharon, Manar Salameh, Ashley Offenheim, Elise Heon, Giorgia Girotto, Paolo Gasparini, Anna Morgan, Arthur A. Bergen, Jacoline B. ten Brink, Caroline C.W. Klaver, Lisbeth Tranebjærg, Nanna D. Rendtorff, Sascha Vermeer, Jeroen J. Smits, Ronald J.E. Pennings, Marco Aben, Jaap Oostrik, Galuh D.N. Astuti, Jordi Corominas Galbany, Hester Y. Kroes, Milan Phan, Wendy A.G. van Zelst-Stams, Alberta A.H.J. Thiadens, Joke B.G.M. Verheij, Mary J. van Schooneveld, Suzanne E. de Bruijn, Catherina H.Z. Li, Carel B. Hoyng, Christian Gilissen, Lisenka E.L.M. Vissers, Frans P.M. Cremers, Hannie Kremer, Erwin van Wijk, and Susanne Roosing

Table of Contents

Figure S1: Effects on pre-mRNA splicing observed in minigene splice assays for thirteen variants....... 2

Figure S2: Position of antisense oligonucleotides (AONs) and 3 nucleotide mismatch AONs (3ntMM AONs) relative to their targets. 4

Figure S3: Differentiation efficiency of patient-derived photoreceptor precursor cells (PPCs). 5

Table S1: Pre-screening methods of all samples 6

Table S2: All genes associated with Usher(-like) syndrome and autosomal recessive retinitis pigmentosa that were assessed in genome sequencing data of 100 cases. 9

Table S3: Sequences of primers used to generate constructs for the minigene splice assays. 10

Table S4: Sequences of primers used in qPCR. 11

Table S5*: All *USH2A* variants that met our variant inclusion criteria in 100 cases. 12

Table S6*: All *USH2A* variants with a predicted effect on splicing.. 13

Table S7: Overview of observed effects on pre-mRNA splicing and consequences on protein level of 21 variants that were tested with minigene splice assays. 14

Table S8: Characteristics of all oligonucleotides designed in this study 15

Supplemental references 16

* Table S5 and Table S6 are separate excel files

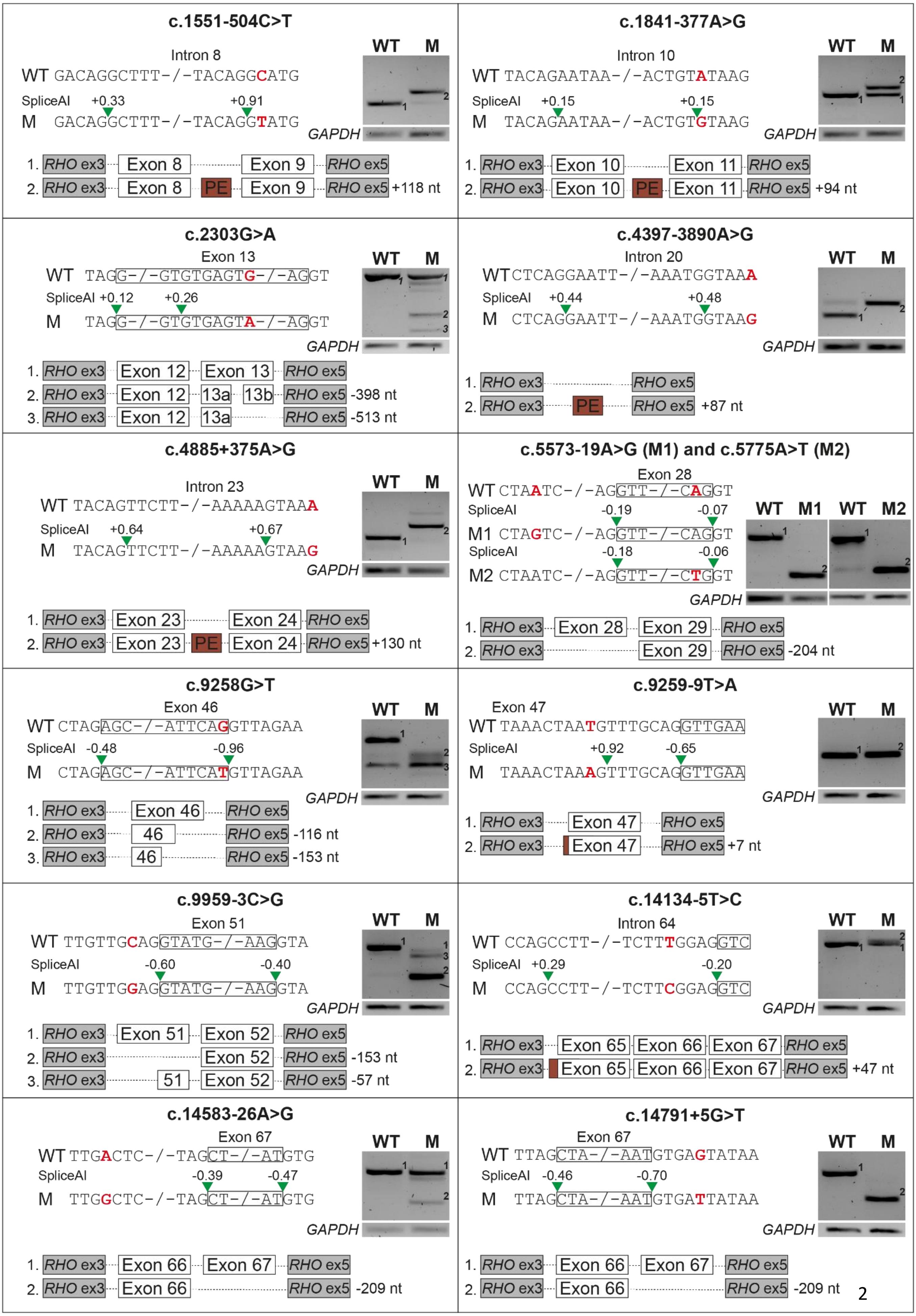
c.1551-504C>T
Intron 8
WT GACAGGCTTT-/-TACAGGCATG
SpliceAI +0.33 +0.91
M GACAGGCTTT-/-TACAGGTATG
WT M
GAPDH
1. RHO ex3 Exon 8 Exon 9 RHO ex5
2. RHO ex3 Exon 8 PE Exon 9 RHO ex5 +118 nt
c.1841-377A>G
Intron 10
WT TACAGAATAA-/-ACTGTATAAG
SpliceAI +0.15 +0.15
M TACAGAATAA-/-ACTGTGTAAG
1. RHO ex3 Exon 10 Exon 11 RHO ex5
2. RHO ex3 Exon 10 PE Exon 11 RHO ex5 +94 nt
c.2303G>A
Exon 13
WT TAGG-/-GTGTGAGTG-/-AGGT
SpliceAI +0.12 +0.26
M TAGG-/-GTGTGAGTA-/-AGGT
1. RHO ex3 Exon 12 Exon 13 RHO ex5
2. RHO ex3 Exon 12 13a 13b RHO ex5 -398 nt
3. RHO ex3 Exon 12 13a RHO ex5 -513 nt
c.4397-3890A>G
Intron 20
WT CTCAGGAATT-/-AAATGGTAAA
SpliceAI +0.44 +0.48
M CTCAGGAATT-/-AAATGGTAAG
1. RHO ex3 RHO ex5
2. RHO ex3 PE RHO ex5 +87 nt
c.4885+375A>G
Intron 23
WT TACAGTTCTT-/-AAAAAGTAAA
SpliceAI +0.64 +0.67
M TACAGTTCTT-/-AAAAAGTAAG
1. RHO ex3 Exon 23 Exon 24 RHO ex5
2. RHO ex3 Exon 23 PE Exon 24 RHO ex5 +130 nt
c.5573-19A>G (M1) and c.5775A>T (M2)
Exon 28
WT CTAATC-/-AGGTT-/-CAGGT
SpliceAI -0.19 -0.07
M1 CTAGTC-/-AGGTT-/-CAGGT
SpliceAI -0.18 -0.06
M2 CTAATC-/-AGGTT-/-CTGGT
WT M1 WT M2
1. RHO ex3 Exon 28 Exon 29 RHO ex5
2. RHO ex3 Exon 29 RHO ex5 -204 nt
c.9258G>T
Exon 46
WT CTAGAGC-/-ATTCAGGTTAGAA
SpliceAI -0.48 -0.96
M CTAGAGC-/-ATTCATGTTAGAA
1. RHO ex3 Exon 46 RHO ex5
2. RHO ex3 46 RHO ex5 -116 nt
3. RHO ex3 46 RHO ex5 -153 nt
c.9259-9T>A
Exon 47
WT TAAACTAATGTTTGCAGGTTGAA
SpliceAI +0.92 -0.65
M TAAACTAAAGTTTGCAGGTTGAA
1. RHO ex3 Exon 47 RHO ex5
2. RHO ex3 Exon 47 RHO ex5 +7 nt
c.9959-3C>G
Exon 51
WT TTGTTGCAGGTATG-/-AAGGTA
SpliceAI -0.60 -0.40
M TTGTTGGAGGTATG-/-AAGGTA
1. RHO ex3 Exon 51 Exon 52 RHO ex5
2. RHO ex3 Exon 52 RHO ex5 -153 nt
3. RHO ex3 51 Exon 52 RHO ex5 -57 nt
c.14134-5T>C
Intron 64
WT CCAGCCTT-/-TCTTTGGAGGTC
SpliceAI +0.29 -0.20
M CCAGCCTT-/-TCTTCGGAGGTC
1. RHO ex3 Exon 65 Exon 66 Exon 67 RHO ex5
2. RHO ex3 Exon 65 Exon 66 Exon 67 RHO ex5 +47 nt
c.14583-26A>G
Exon 67
WT TTGACTC-/-TAGCT-/-ATGTG
SpliceAI -0.39 -0.47
M TTGGCTC-/-TAGCT-/-ATGTG
1. RHO ex3 Exon 66 Exon 67 RHO ex5
2. RHO ex3 Exon 66 RHO ex5 -209 nt
c.14791+5G>T
Exon 67
WT TTAGCTA-/-AATGTGAGTATAA
SpliceAI -0.46 -0.70
M TTAGCTA-/-AATGTGATTATAA
1. RHO ex3 Exon 66 Exon 67 RHO ex5
2. RHO ex3 Exon 66 RHO ex5 -209 nt

Figure S1: Effects on pre-mRNA splicing observed in minigene splice assays for thirteen variants. Thirteen of the 21 tested variants revealed differences in pre-mRNA splicing between mutant (M) and wildtype (WT) constructs in our minigene splice assays. The effects observed after RT-PCR are shown as well as a schematic representation of these effects including the *RHO* exons 3 and 5 that are flanking the *USH2A* genomic region cloned in the minigene. Splice predictions were obtained with SpliceAI. nt: nucleotides PE: pseudoexon

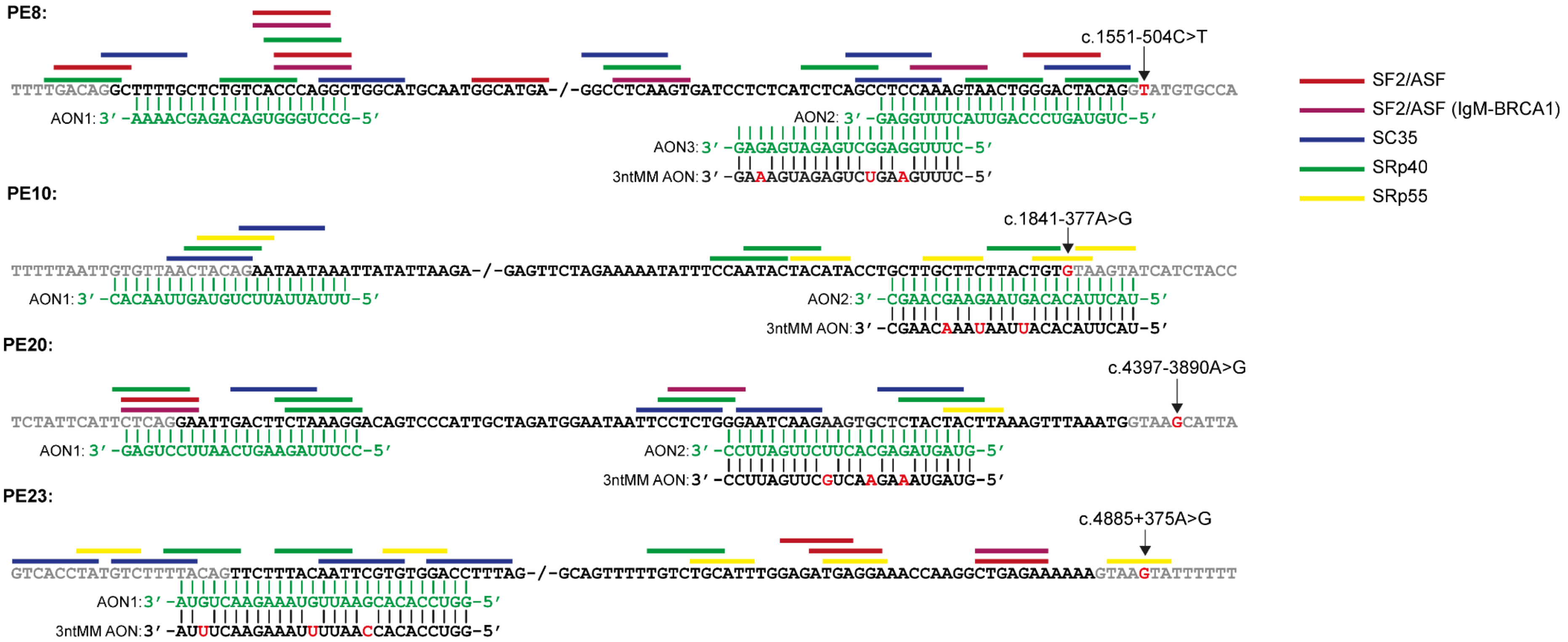

Figure S2: Position of antisense oligonucleotides (AONs) and 3 nucleotide mismatch AONs (3ntMM AONs) relative to their targets.
AONs were designed complementary to the splice sites or exonic splice enhancers of each specific pseudoexon (PE). The sequence of each AON is shown in green, 3ntMM AONs are in black with mismatches in red. The PE sequences that are incorporated in the mature *USH2A* transcript as a consequence of the identified deep-intronic variants are depicted in black, intronic sequences are in grey and the identified genetic variants are indicated in red. The bars above the sequences represent different types of putative exonic splice enhancers based on predictions from the ESEfinder tool[1] accessed from Alamut Visual Plus v1.4.

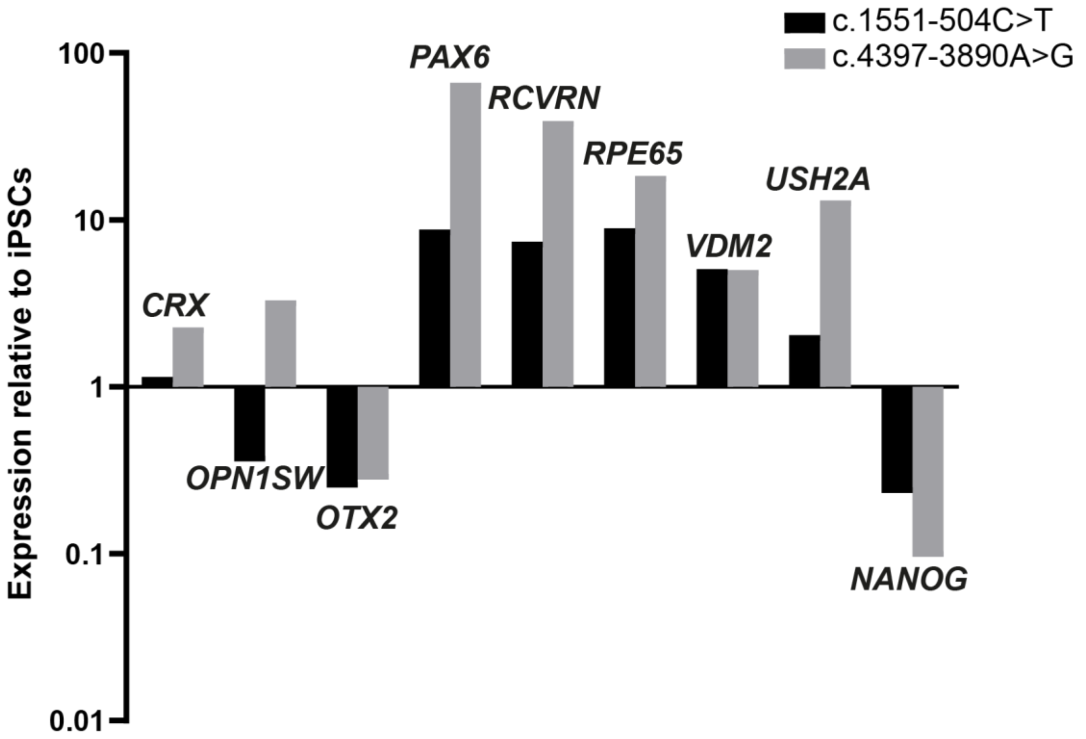

Figure S3: Differentiation efficiency of patient-derived photoreceptor precursor cells (PPCs). Expression of several neuronal progenitor, photoreceptor and retinal pigment epithelium markers (*CRX, OPN1SW, OTX2, PAX6, RCVRN, RPE65, VDM2*) as well as expression of *USH2A* and a pluripotency marker (*NANOG*) were determined with RT-qPCR in induced pluripotent stem cells (iPSCs) and compared PPCs after 30 days of differentiation. Expression was normalized against *GUSB* and compared to the corresponding iPSC line at the start of differentiation (day 0) using "delta delta Ct". As expected, pluripotency markers decreased while most of the other markers showed an increased. These data indicate that the differentiation of iPSCs towards PPCs was successful for both cell lines.

Table S1: Pre-screening methods of all samples

Study ID	Causal gene	Status of proband	Pre-screening
arRP1	*USH2A*	Possibly solved	Molecular inversion probe based sequencing
arRP2	-	Unsolved	Molecular inversion probe based sequencing
arRP3	-	Unsolved	Molecular inversion probe based sequencing
arRP4	*PQLC2*	Solved	Molecular inversion probe based sequencing
arRP5	-	Unsolved	Whole exome sequencing
arRP6	*EYS*	Possibly solved	Whole exome sequencing
arRP7	-	Unsolved	Whole exome sequencing
arRP8	*USH2A*	Possibly solved	Whole exome sequencing
arRP9	-	Unsolved	Whole exome sequencing
arRP10	*USH2A*	Possibly solved	Whole exome sequencing
arRP11	*USH2A*	Solved	Target 5000 sequencing
arRP12	-	Unsolved	Target 5000 sequencing
arRP13	-	Unsolved	Target 5000 sequencing
arRP14	-	Unsolved	Target 5000 sequencing
arRP15	-	Unsolved	Whole exome sequencing
arRP16	-	Unsolved	Whole exome sequencing
arRP17	-	Unsolved	Targeted sequencing
arRP18	-	Unsolved	Target 5000 sequencing
arRP19	*USH2A*	Solved	Molecular inversion probe based sequencing
arRP20	*USH2A*	Solved	Molecular inversion probe based sequencing
arRP21	*USH2A*	Possibly solved	Molecular inversion probe based sequencing
arRP22	*USH2A*	Solved	Molecular inversion probe based sequencing
arRP23	-	Unsolved	Molecular inversion probe based sequencing
arRP24	*USH2A*	Possibly solved	Molecular inversion probe based sequencing
arRP25	-	Unsolved	Molecular inversion probe based sequencing
arRP26	*USH2A*	Solved	Molecular inversion probe based sequencing
arRP27	-	Unsolved	Molecular inversion probe based sequencing
arRP28	-	Unsolved	Cegat panel sequencing
arRP29	*PROM1*	Possibly solved	Cegat panel sequencing
arRP30	-	Unsolved	Cegat panel sequencing
arRP31	-	Unsolved	Cegat panel sequencing
arRP32	-	Unsolved	Molecular inversion probe based sequencing
arRP33	-	Unsolved	Sanger sequencing
arRP34	*USH2A*	Solved	Micro array and Sanger sequencing
arRP35	-	Unsolved	Whole exome sequencing
arRP36	*USH2A*	Solved	Whole exome sequencing
arRP37	*RPE65*	Solved	Whole exome sequencing
arRP38	-	Unsolved	Whole exome sequencing
arRP39	-	Unsolved	Whole exome sequencing
arRP40	-	Unsolved	Whole exome sequencing
arRP41	*USH2A*	Solved	Whole exome sequencing

arRP42	*USH2A*	Solved	Whole exome sequencing
arRP43	-	Unsolved	Whole exome sequencing
arRP44	-	Unsolved	Whole exome sequencing
arRP45	-	Unsolved	Whole exome sequencing
arRP46	-	Unsolved	Whole exome sequencing
arRP47	-	Unsolved	Whole exome sequencing
CRD1	-	Unsolved	Molecular inversion probe based sequencing
DFNB1	-	Unsolved	Targeted sequencing
DFNB2	*USH2A*	Solved	Targeted sequencing
DFNB3	-	Unsolved	Targeted sequencing
USH1	-	Unsolved	Molecular inversion probe based sequencing and multiplex ligation-dependent probe amplification
USH2	-	Unsolved	Whole exome sequencing
USH3	-	Unsolved	Whole exome sequencing
USH4	-	Unsolved	Molecular inversion probe based sequencing and whole exome sequencing
USH5	*USH2A*	Solved	Targeted sequencing
USH6	-	Unsolved	Targeted sequencing
USH7	*USH2A*	Solved	Targeted sequencing
USH8	*USH2A*	Solved	Targeted sequencing
USH9	-	Unsolved	Targeted sequencing
USH10	*USH2A*	Solved	Molecular inversion probe based sequencing
USH11	*PEX6*	Solved	Molecular inversion probe based sequencing
USH12	*USH2A*	Solved	Targeted resequencing panel of 10 USH genes
USH13	*USH2A*	Solved	Targeted resequencing panel of 10 USH genes
USH14	-	Unsolved	Whole exome sequencing
USH15	-	Unsolved	Whole exome sequencing
USH16	*USH2A*	Solved	Whole exome sequencing
USH17	*USH2A*	Solved	Target 5000 sequencing
USH18	-	Unsolved	Target 5000 sequencing
USH19	*USH2A*	Solved	Target 5000 sequencing
USH20	*ARSG*	Solved	Target 5000 sequencing
USH21	-	Unsolved	Target 5000 sequencing
USH22	*USH2A*	Solved	Target 5000 sequencing
USH23	*USH2A*	Possibly solved	Target 5000 sequencing
USH24	*USH2A*	Solved	Targeted sequencing of 13 USH genes and multiplex ligation-dependent probe amplification
USH25	*MYO7A*	Solved	Whole exome sequencing
USH26	*USH2A*	Solved	Whole exome sequencing
USH27	-	Unsolved	Targeted sequencing
USH28	*USH2A*	Solved	Treatrush sequencing
USH29	*USH2A*	Possibly solved	Treatrush sequencing
USH30	-	Unsolved	Treatrush sequencing

USH31	*USH2A*	Solved	Treatrush sequencing
USH32	*USH2A*	Possibly solved	Cegat panel sequencing
USH33	*USH2A*	Solved	Treatrush sequencing
USH34	*USH2A*	Solved	Treatrush sequencing
USH35	*ARSG*	Solved	Cegat panel sequencing
USH36	-	Unsolved	Cegat panel sequencing
USH37	*USH2A*	Solved	Sanger sequencing
USH38	-	Unsolved	Whole exome sequencing
USH39	*USH2A*	Solved	Micro array and Sanger sequencing
USH40	-	Unsolved	Whole exome sequencing
USH41	*USH2A*	Solved	Micro array and Sanger sequencing
USH42	*USH2A*	Solved	Molecular inversion probe based sequencing
USH43	-	Unsolved	Whole exome sequencing
USH44	*USH2A*	Solved	Whole exome sequencing
USH45	-	Unsolved	Whole exome sequencing
USH46	*USH2A*	Possibly solved	Whole exome sequencing
USH47	-	Unsolved	Whole exome sequencing
USH48	-	Unsolved	Whole exome sequencing
USH49	*USH2A*	Possibly solved	Targeted sequencing and multiplex ligation-dependent probe amplification

Table S2: All genes associated with Usher(-like) syndrome and autosomal recessive retinitis pigmentosa that were assessed in genome sequencing data of 100 cases.

**Autosomal recessive retinitis pigmentosa**	*ABCA4*	*AGBL5*	*AHR*	*ARHGEF18*	*ARL2BP*
	ARL6	*BBS1*	*BBS2*	*BEST1*	*C2orf71*
	C8orf37	*CERKL*	*CLCC1*	*CLRN1*	*CNGA1*
	CNGB1	*CRB1*	*CYP4V2*	*DHDDS*	*DHX38*
	EMC1	*EYS*	*FAM161A*	*GPR125 (ADGRA3)*	*HGSNAT*
	IDH3B	*IFT140*	*IFT172*	*IMPG2*	*KIAA1549*
	KIZ	*LRAT*	*MAK*	*MERTK*	*MVK*
	NEK2	*NEUROD1*	*NR2E3*	*NRL*	*PDE6A*
	PDE6B	*PDE6G*	*POMGNT1*	*PRCD*	*PROM1*
	RBP3	*REEP6*	*RGR*	*RHO*	*RLBP1*
	RP1	*RP1L1*	*RPE65*	*SAG*	*SAMD11*
	SLC7A14	*SPATA7*	*TRNT1*	*TTC8*	*TULP1*
	ZNF408	*ZNF513*			
**Usher syndrome**	*ABHD12*	*ADGRV1*	*ARSG*	*CDH23*	*CEP250*
	CEP78	*CIB2*	*CLRN1*	*ESPN*	*HARS1*
	MYO7A	*PCDH15*	*PEX1*	*PEX6*	*USH1C*
	USH1G	*WHRN*			
**Retinal modifier of Usher syndrome**	*PDZD7*				

Table S3: Sequences of primers used to generate constructs for the minigene splice assays.

**Target variant (NM_206933.2)**	**Forward primer (5'>3')**	**Reverse primer (5'>3')**
c.1551-504C>T	GGGGACAAGTTTGTACAAAAAAGCAGGCTTCtcccttcaccaccaacttcc	GGGGACCACTTTGTACAAGAAAGCTGGGTGagtgccatgctatccaaacac
c.1644+7453A>G	GGGGACAAGTTTGTACAAAAAAGCAGGCTTCtaagaggccccaatgtgtgt	GGGGACCACTTTGTACAAGAAAGCTGGGTGtggggcggaagagttaacat
c.1841-377A>G	GGGGACAAGTTTGTACAAAAAAGCAGGCTTCggagatcgagaccatgctgg	GGGGACCACTTTGTACAAGAAAGCTGGGTGatgcaaaggaccaccgaact
c.2303G>A	GGGGACAAGTTTGTACAAAAAAGCAGGCTTCtgcattcttttcaaaccagatgc	GGGGACCACTTTGTACAAGAAAGCTGGGTGtttcaggggacatagggtgg
c.4396+6885T>C	GGGGACAAGTTTGTACAAAAAAGCAGGCTTCacatcaatggaaacgagtgacc	GGGGACCACTTTGTACAAGAAAGCTGGGTGtggaaaggagaaaatgtaggctc
c.4397-3890A>G	GGGGACAAGTTTGTACAAAAAAGCAGGCTTCttctggcctagcagtgtttg	GGGGACCACTTTGTACAAGAAAGCTGGGTGcagggaagcaatggagaacc
c.4714C>T	GGGGACAAGTTTGTACAAAAAAGCAGGCTTCacaattcccgccaaatccttc	GGGGACCACTTTGTACAAGAAAGCTGGGTGtcttcctttccctctggctg
c.4885+375A>G	GGGGACAAGTTTGTACAAAAAAGCAGGCTTCtgtcatacatgttgtcgagctc	GGGGACCACTTTGTACAAGAAAGCTGGGTGaagaggaggagaaggtgcac
c.5573-19A>G	GGGGACAAGTTTGTACAAAAAAGCAGGCTTCtcaccaccatccctctgaag	GGGGACCACTTTGTACAAGAAAGCTGGGTGcaggccacaagatcgagttg
c.5775A>T	GGGGACAAGTTTGTACAAAAAAGCAGGCTTCtcaccaccatccctctgaag	GGGGACCACTTTGTACAAGAAAGCTGGGTGcaggccacaagatcgagttg
c.6806-7599C>G	GGGGACAAGTTTGTACAAAAAAGCAGGCTTCcacatccctgcctcattcac	GGGGACCACTTTGTACAAGAAAGCTGGGTGcactgtctttgctacatcccag
c.8710G>A	GGGGACAAGTTTGTACAAAAAAGCAGGCTTCccatgccacaccacctttg	GGGGACCACTTTGTACAAGAAAGCTGGGTGgctgtaaccaaattcaaggctg
c.9258G>T	GGGGACAAGTTTGTACAAAAAAGCAGGCTTCccaagcaagtgttccaggtc	GGGGACCACTTTGTACAAGAAAGCTGGGTGgatgggttgttaggtgcagc
c.9259-9T>A	GGGGACAAGTTTGTACAAAAAAGCAGGCTTCcagaatgtaggcccttgatagtg	GGGGACCACTTTGTACAAGAAAGCTGGGTGtctcctgagcttgtgatccg
c.9959-3C>G	GGGGACAAGTTTGTACAAAAAAGCAGGCTTCaaacaattcaggaccccagg	GGGGACCACTTTGTACAAGAAAGCTGGGTGgcttgaagtgcatttggagc
c.12343C>T	GGGGACAAGTTTGTACAAAAAAGCAGGCTTCagatatggcagtccccttcc	GGGGACCACTTTGTACAAGAAAGCTGGGTGtgttccctgtattcactgtactc
c.14134-5T>C	GGGGACAAGTTTGTACAAAAAAGCAGGCTTCtgctagctccttacttccctg	GGGGACCACTTTGTACAAGAAAGCTGGGTGtgcagaaatgatggtggttcc
c.14583-26A>G	GGGGACAAGTTTGTACAAAAAAGCAGGCTTCcgttaacacgtttgaggcac	GGGGACCACTTTGTACAAGAAAGCTGGGTGgccacgggaaatgcaaatac
c.14664G>A	GGGGACAAGTTTGTACAAAAAAGCAGGCTTCcgttaacacgtttgaggcac	GGGGACCACTTTGTACAAGAAAGCTGGGTGgccacgggaaatgcaaatac
c.14753C>T	GGGGACAAGTTTGTACAAAAAAGCAGGCTTCcgttaacacgtttgaggcac	GGGGACCACTTTGTACAAGAAAGCTGGGTGgccacgggaaatgcaaatac
c.14791+5G>T	GGGGACAAGTTTGTACAAAAAAGCAGGCTTCccagcacgatgaagactcttg	GGGGACCACTTTGTACAAGAAAGCTGGGTGtctgggtggaggggtataca
RHO exons 3 to 5 (RT-PCR)	cggaggtcaacaacgagtct	aggtgtaggggatgggagac
GAPDH control (RT-PCR)	ctgcaccaccaactgcttag	agctcagggatgaccttgc

The sequence of the Gateway® attB site is depicted in upper case, the *USH2A*-specific sequence in lower case letters.

Table S4: Sequences of primers used in qPCR.

Target	Remark	Forward primer (5'>3')	Reverse primer (5'>3')
GUSB	Housekeeping gene	AGAGTGGTGCTGAGGATTGG	CCCTCATGCTCTAGCGTGTC
CRX		CCCCAGTGTGGATCTGATG	CAAACAGTGCCTCCAGCTC
NANOG		CCTGTGATTTGTGGGCCTG	CAGTCTCCGTGTGAGGCAT
OPN1SW		TTCTTCTCCAAGAGTGCTTGC	CCTTCCCACACACCATCTTC
OTX2		TATCTTAAGCAACCGCCTTACG	GGAGGGGTGCAGCAAGTC
PAX6		GCTGCAAAGAAATAGAACATCC	TTGGCTGCTAGTCTTTCTCG
RCVRN		ACACCAAGTTCTCGGAGGAG	ACTTGGCGTAGATGCTCTGG
RPE65		TTACTACGCTTGCACAGAGACC	GCCCCATTGACAGAGACATAG
VDM2		TCAGTGTGGACACCTGTATGC	AAGCTGTACACCGCCACAG
USH2A Exon 8-9	Specific for wildtype transcript	ACAACTGAGACTGCTGTTAACC	GACCATGGCACTGACATCTC
USH2A exon 8-PE8	Specific for transcript with PE	ACAACTGAGACTGCTGTTAACC	CAGAGCAAAAGCCTCCCAC
USH2A exon 12-13	Reference for expression levels	GCAAAGCAAACGTTATTGGGCT	TACACTGGCAGGGCTCACAT
USH2A exon 20-21	Specific for wildtype transcript	ACTTTAGCAGCAGCACCAGC	GGAGAGGGTCCATTCAGTTC
USH2A PE20-exon 21	Specific for transcript with PE	GGACAGTCCCATTGCTAGATG	AGTTCTTGGGATTTAGCAGTGTG

PE: pseudoexon

Table S5: All *USH2A* variants that met our variant inclusion criteria in 100 cases.

See separate excel sheet

Table S6: All *USH2A* variants with a predicted effect on splicing.

See separate excel sheet

Table S7: Overview of observed effects on pre-mRNA splicing and consequences on protein level of 21 variants that were tested with minigene splice assays.

Variant	Effect on splicing	Protein effect
**Deep-intronic variants**		
c.1551-504C>T	PE inclusion (Δ118 nt)	p.Arg517_Cys518ins*13
c.1644+7453A>G	No effect	p.=
c.1841-377A>G	1. PE inclusion (Δ94 nt), 2. No effect	p.[Gly614Aspfs*9,=]
c.4396+6885T>C	No effect	p.=
c.4397-3890A>G	PE inclusion (Δ87 nt)	p.Ala1465_Ala1466ins*5
c.4885+375A>G[2]	PE inclusion (Δ130 nt)	p.Ser1629Valfs*52
c.6806-7599C>G	No effect	p.=
**Non-canonical splice site variants**		
c.5775A>T	Exon 28 skipping (Δ204 nt)	p.Gly1858_Thr1925del
c.9258G>T	1. Partial exon 46 skipping (Δ153 nt), 2. Partial exon 46 skipping (Δ116 nt)	p.[Arg3037_Val3087del,Val3049*]
c.9259-9T>A	Inclusion of last 7 nt of intron 46 to exon 47	p.Val3087Phefs*4
c.9959-3C>G	1. Exon 51 skipping (Δ224 nt), 2. Partial exon 51 skipping (Δ57 nt)	p.[Met3321Asnfs*22,=,Gly3320_Ser3338del]
c.14134-5T>C	1. No effect, 2. Intron 64 inclusion (Δ47 nt)	p.[=,Val4712Profs*2]
c.14791+5G>T	Exon 67 skipping (Δ209 nt)	p.Tyr4862Alafs*22
**Exonic variants**		
c.2303G>A	1. No effect, 2. Partial skipping of center exon 13 (Δ398 nt), 3. Partial exon 13 skipping (Δ513 nt)	p.[Cys768Tyr,Cys766Tyrfs*3,Glu767_Gly937del]
c.4714C>T	No effect	p.(Leu1572Phe)
c.8710G>A	No effect	p.(Val2904Ile)
c.12343C>T	No effect	p.(Arg4115Cys)
c.14664G>A	No effect	p.(Thr4888=)
c.14753C>T	No effect	p.(Thr4918Met)
**Branchpoint variants**		
c.5573-19A>G	Exon 28 skipping (Δ204 nt)	p.Gly1858_Thr1925del
c.14583-26A>G	1. No effect, 2. Exon 67 skipping (Δ209 nt)	p.[=,Tyr4862Alafs*22]

nt: nucleotide; PE: pseudoexon

Table S8: Characteristics of all oligonucleotides designed in this study

Oligonucleotide	Sequence (3’>5’)	Target variant	Coordinates of pseudoexon (hg19)	Length (nt)	GC-content (%)
AON1 USH2A PE8	GCCUGGGUGACAGAGCAAAA	c.1551-504C>T (p.Arg517_Cys518ins*13)	chr1:216495824-216495941	20	55
AON2 USH2A PE8^	CUGUAGUCCCAGUUACUUUGGAG			23	48
AON3 USH2A PE8	CUUUGGAGGCUGAGAUGAGAG			21	52
SON USH2A PE8	CUCUCAUCUCAGCCUCCAAAG			21	52
AON3 3ntMM USH2A PE8	CUUUGaAGuCUGAGAUGAaAG			21	38
AON1 USH2A PE10	UUUAUUAUUCUGUAGUUAACAC	c.1841-377A>G (p.[Thr613_Gly614ins*9,=])	chr1:216463130-216463223	22	23
AON2 USH2A PE10	UACUUACACAGUAAGAAGCAAGC			23	39
AON2 3ntMM USH2A PE10	UACUUACACAuUAAuAAaCAAGC			23	26
SON USH2A PE10*	GUAGUAAAGAACUGCUUGAUUCC			23	39
AON1 USH2A PE20	CCUUUAGAAGUCAAUUCCUGAG	c.4397-3890A>G (p.Ala1465_Ala1466ins*5)	chr1:216352719-216352805	22	41
AON2 USH2A PE20	GUAGUAGAGCACUUCUUGAUUCC			23	43
SON USH2A PE20	GGAAUCAAGAAGUGCUCUACUAC			23	43
AON2 3ntMM USH2A PE20*	GUAGUAaAGaACUgCUUGAUUCC			23	39
AON1 USH2A PE23	GGUCCACACGAAUUGUAAAGAACUGUA	c.4885+375A>G[2] (p.Ser1629Valfs*52)	chr1:216261985-216262114	27	41
SON USH2A PE23*	GUAGUAAAGAACUGCUUGAUUCC			27	33
AON1 3ntMM USH2A PE23	GGUCCACACcAAUUuUAAAGAACUuUA			23	39

Oligonucleotides labeled with an asterisk are the same. For 3 nucleotide mismatch antisense oligonucleotides (3ntMM AONs), lowercase nucleotides are mismatches. PE: pseudoexon; SON: sense oligonucleotide; ^: many off-target binding regions predicted for this oligonucleotide

Supplemental references

1. Cartegni, L., Wang, J., Zhu, Z., Zhang, M. Q. & Krainer, A. R. ESEfinder: A web resource to identify exonic splicing enhancers. Nucleic Acids Res 31, 3568-3571, doi:10.1093/nar/gkg616 (2003).
2. Mansard, L. et al. The Study of a 231 French Patient Cohort Significantly Extends the Mutational Spectrum of the Two Major Usher Genes MYO7A and USH2A. Int J Mol Sci 22, doi:10.3390/ijms222413294 (2021).
3. Liquori, A. et al. Whole USH2A Gene Sequencing Identifies Several New Deep Intronic Mutations. Hum Mutat 37, 184-193, doi:10.1002/humu.22926 (2016).
4. Vaché, C. *et al.* Usher syndrome type 2 caused by activation of an USH2A pseudoexon: implications for diagnosis and therapy. *Hum Mutat* **33**, 104-108, doi:10.1002/humu.21634 (2012).
5. Lenassi, E. et al. A detailed clinical and molecular survey of subjects with nonsyndromic USH2A retinopathy reveals an allelic hierarchy of disease-causing variants. Eur J Hum Genet 23, 1318-1327, doi:10.1038/ejhg.2014.283 (2015).
