# Supplementary material for: Whole genome sequencing for USH2A-associated disease reveals several pathogenic deep-intronic variants that are amenable to splice correction
Source: HGG Adv. 2023 Jan 18;4(2):100181. doi: 10.1016/j.xhgg.2023.100181 (PMC9918427; doi:10.1016/j.xhgg.2023.100181)
Supplement: Document S2. Article plus supplemental information [file mmc4.pdf]

HGG Advances

ARTICLE

Whole genome sequencing for *USH2A*-associated disease reveals several pathogenic deep-intronic variants that are amenable to splice correction

Janine Reurink,[1,2] Nicole Weisschuh,[3] Alejandro Garanto,[1,4,5] Adrian Dockery,[6] L. Ingeborgh van den Born,[7] Isabelle Fajardy,[8] Lonneke Haer-Wigman,[1,2] Susanne Kohl,[3] Bernd Wissinger,[3] G. Jane Farrar,[6] Tamar Ben-Yosef,[9] Fatma Kivrak Pfiffner,[10] Wolfgang Berger,[10,11,12] Marianna E. Weener,[13] Lubica Dudakova,[14] Petra Liskova,[14,15] Dror Sharon,[16] Manar Salameh,[16] Ashley Offenheim,[16] Elise Heon,[17] Giorgia Girotto,[18,19] Paolo Gasparini,[18,19] Anna Morgan,[18] Arthur A. Bergen,[20,21] Jacoline B. ten Brink,[20] Caroline C.W. Klaver,[22,23,24] Lisbeth Tranebjærg,[25,26] Nanna D. Rendtorff,[25] Sascha Vermeer,[27] Jeroen J. Smits,[2,28,29] Ronald J.E. Pennings,[28] Marco Aben,[1] Jaap Oostrik,[28] Galuh D.N. Astuti,[1,30] Jordi Corominas Galbany,[1] Hester Y. Kroes,[29] Milan Phan,[22,31] Wendy A.G. van Zelst-Stams,[1] Alberta A.H.J. Thiadens,[23] Joke B.G.M. Verheij,[32] Mary J. van Schooneveld,[21] Suzanne E. de Bruijn,[1,2] Catherina H.Z. Li,[2,22] Carel B. Hoyng,[2,22] Christian Gilissen,[1,5] Lisenka E.L.M. Vissers,[1,2] Frans P.M. Cremers,[1,2] Hannie Kremer,[1,2,28] Erwin van Wijk,[2,28,*] and Susanne Roosing[1,2,33,*]

Summary

A significant number of individuals with a rare disorder such as Usher syndrome (USH) and (non-)syndromic autosomal recessive retinitis pigmentosa (arRP) remain genetically unexplained. Therefore, we assessed subjects suspected of *USH2A*-associated disease and no or mono-allelic *USH2A* variants using whole genome sequencing (WGS) followed by an improved pipeline for variant interpretation to provide a conclusive diagnosis.

One hundred subjects were screened using WGS to identify causative variants in *USH2A* or other USH/arRP-associated genes. In addition to the existing variant interpretation pipeline, a particular focus was put on assessing splice-affecting properties of variants, both *in silico* and *in vitro*. Also structural variants were extensively addressed. For variants resulting in pseudoexon inclusion, we designed and evaluated antisense oligonucleotides (AONs) using minigene splice assays and patient-derived photoreceptor precursor cells.

Biallelic variants were identified in 49 of 100 subjects, including novel splice-affecting variants and structural variants, in *USH2A* or arRP/USH-associated genes. Thirteen variants were shown to affect *USH2A* pre-mRNA splicing, including four deep-intronic *USH2A* variants resulting in pseudoexon inclusion, which could be corrected upon AON treatment.

We have shown that WGS, combined with a thorough variant interpretation pipeline focused on assessing pre-mRNA splicing defects and structural variants, is a powerful method to provide subjects with a rare genetic condition, a (likely) conclusive genetic diagnosis. This is essential for the development of future personalized treatments and for patients to be eligible for such treatments.

[1]Department of Human Genetics, Radboud University Medical Center, Nijmegen, the Netherlands; [2]Donders Institute for Brain Cognition and Behaviour, Radboud University Medical Center, Nijmegen, the Netherlands; [3]Molecular Genetics Laboratory, Institute for Ophthalmic Research, Centre for Ophthalmology, University of Tübingen, Tübingen, Germany; [4]Department of Pediatrics, Amalia's Children Hospital, Radboud University Medical Center, Nijmegen, The Netherlands; [5]Radboud Institute of Molecular Life Sciences, Radboud University Medical Center, Nijmegen, the Netherlands; [6]The School of Genetics & Microbiology, Smurfit Institute of Genetics, Trinity College Dublin, Dublin 2, Ireland; [7]The Rotterdam Eye Hospital, Rotterdam, the Netherlands; [8]Centre de Biologie Pathologie Génétique, CHU de Lille, Lille, France; [9]The Rappaport Faculty of Medicine, Technion-Israel Institute of Technology, Haifa, Israel; [10]Institute of Medical Molecular Genetics, University of Zurich, Schlieren, Switzerland; [11]Neuroscience Center Zurich, University and ETH Zurich, Zurich, Switzerland; [12]Center for Integrative Human Physiology, University of Zurich, Zurich, Switzerland; [13]CRO Oftalmic, Moscow, Russia; [14]Department of Paediatrics and Inherited Metabolic Disorders, First Faculty of Medicine, Charles University and General University Hospital in Prague, Prague, Czech Republic; [15]Department of Ophthalmology, First Faculty of Medicine, Charles University and General University Hospital in Prague, Prague, Czech Republic; [16]Division of Ophthalmology, Hadassah University Medical Center, Faculty of Medicine, The Hebrew University of Jerusalem, Jerusalem, Israel; [17]Departments of Ophthalmology and Vision Sciences, The Hospital for Sick Children, The University of Toronto, Toronto, ON, Canada; [18]Institute for Maternal and Child Health—I.R.C.C.S. "Burlo Garofolo", 34137 Trieste, Italy; [19]Department of Medicine, Surgery and Health Sciences, University of Trieste, 34149 Trieste, Italy; [20]Department of Clinical Genetics, Amsterdam UMC, University of Amsterdam, 1105 Amsterdam, the Netherlands; [21]Department of Ophthalmology, Amsterdam UMC, University of Amsterdam, 1105 Amsterdam, the Netherlands; [22]Department of Ophthalmology, Radboud University Medical Center, Nijmegen, the Netherlands; [23]Department of Ophthalmology, Erasmus Medical Center, Rotterdam, the Netherlands; [24]Department of Epidemiology, Erasmus Medical Center, Rotterdam, the Netherlands; [25]Department of Clinical Genetics, The Kennedy Center, Copenhagen University Hospital, 2600 Glostrup, Denmark; [26]Institute of Clinical Medicine, University of Copenhagen, 2200 Copenhagen, Denmark; [27]Center for Human Genetics, University Hospitals Leuven, Leuven, Belgium; [28]Hearing & Genes, Department of Otorhinolaryngology, Radboud University Medical Center, Nijmegen, the Netherlands; [29]Division Laboratories, Pharmacy and Biomedical Genetics, Department of Genetics, University Medical Center of Utrecht, Utrecht, the Netherlands; [30]Division of Human Genetics, Center for Biomedical Research (CEBIOR), Faculty of Medicine, Diponegoro University, Semarang, Indonesia; [31]Department of Ophthalmology, Rijnstate Hospital, Arnhem, the Netherlands; [32]Department of Medical Genetics, University Medical Center Groningen, University of Groningen, Groningen, the Netherlands

[33]Lead contact

*Correspondence: erwin.vanwyk@radboudumc.nl (E.v.W.), susanne.roosing@radboudumc.nl (S.R.)

https://doi.org/10.1016/j.xhgg.2023.100181.

© 2023 The Author(s). This is an open access article under the CC BY license (http://creativecommons.org/licenses/by/4.0/).

Introduction

Whole genome sequencing (WGS) is increasingly recognized as a vital technique in (diagnostic) genetic screening of disease, including inherited retinal diseases (IRDs), such as *USH2A*-associated Usher syndrome type 2 (USH2, MIM: #276901) and autosomal recessive retinitis pigmentosa (arRP, MIM: #613809). The added value compared with whole exome sequencing (WES) and most targeted sequencing panels is that it detects (novel) deep-intronic variants,[1–3] structural variants (SVs),[4,5] and variants in regulatory regions,[6] resulting in an increased diagnostic yield in IRD cases.[4,7,8]

In previous studies, WES and targeted sequencing panels solved 36% to 63% of persons with non-syndromic arRP and 84% to 90% of Usher syndrome (USH) cases.[4,9] We hypothesized that a substantial portion of the remaining unsolved cases has an SV or a deleterious deep-intronic variant that potentially results in inclusion of a pseudoexon (PE). To date, 80 (likely) pathogenic SVs (larger than 50 nucleotides) have already been submitted to ClinVar (March 29, 2022) and several deep-intronic variants resulting in PE inclusion have been reported for *USH2A*.[1–3] Therefore, it is plausible that novel SVs and deep-intronic variants that cause PE inclusion will be identified when genetically unexplained cases are screened with WGS.

The *USH2A* gene is one of the most frequently mutated IRD-associated genes, implicated in both USH2 and arRP, as pathogenic variants are identified in 57% to 79% and 12% to 25% of affected individuals, respectively.[10] *USH2A*-associated IRD is still considered untreatable. However, several gene-specific treatment options are currently in different stages of (pre-)clinical development, including splice-modulation using antisense oligonucleotides (AONs),[11] CRISPR-Cas9-based gene correction,[12] and exon-excision.[13] *Ultevursen*, an AON that induces the skipping of *USH2A* exon 13, is currently being evaluated in a phase II/III clinical trial (ClinicalTrials.gov Identifier: NCT05158296). These developments further stress the importance for subjects to obtain a conclusive genetic diagnosis in order to be eligible for future personalized therapies.

In this study, we aimed to identify missing variants in *USH2A* in a cohort of 100 largely mono-allelic arRP and USH2 subjects through WGS, assess an effect on splicing of all detected single nucleotide variants (SNVs), and to develop an AON-based splice correction strategy for specific deep-intronic variants. In addition, our approach could serve as a future framework for detection and treatment of variants in genes associated with other hereditary disorders.

Material and methods

Ethics declaration

Written informed consent was obtained by the corresponding centers, adherent to the tenets of the declaration of Helsinki and as approved by the local ethics committee of the Radboud University Medical Center Nijmegen, as an amendment to the approval by the local ethics committee of the Rotterdam Eye Hospital (MEC-2010-359; OZR protocol no. 2009-32).

Whole genome sequencing

DNA of unrelated probands was collected from subjects from the Netherlands, Germany, Ireland, Northern Ireland, Israel, Russia, Czech Republic, Switzerland, France, Italy, Denmark, Canada, and Belgium. Subjects were included if they were clinically diagnosed with arRP (n = 47), USH2 (n = 49), or autosomal recessive hearing impairment (DFNB, n = 3) in those who were still in the first or second decade of life. One subject with cone-rod dystrophy (CRD) and hearing loss was included. In addition, we required that pre-screening of the (majority of) *USH2A* exons was performed and only one (potentially or likely) pathogenic variant in *USH2A* was identified or no *USH2A* variants were identified. In 67 subjects, a (potentially or likely) pathogenic variant in *USH2A* was reported prior to WGS screening (34 for RP, 30 for USH, and three for DFNB). *USH2A* variants that were identified in probands prior to their inclusion in our WGS analysis pipeline are shown in Table 1 and screening methods that were used prior to WGS are listed in Table S1.

WGS was performed as previously described.[8,18] Variants in the entire *USH2A* gene (chr1:215796236–216596738), 10 previously determined potential regulatory regions of *USH2A*,[6] the known retinal modifier of *USH2A*-associated disease *PDZD7*,[19] and variants in the entire genomic regions of other genes associated with USH (n = 17, Table S2; RetNet; visited May 21, 2021) and arRP (n = 62, Table S2) were extracted and evaluated. Variants with a gnomAD allele frequency (AF) $\leq$ 1% in the general population and in any subpopulation were prioritized. SNVs were considered potentially pathogenic if they were (1) a stop gain variant, frameshift variant, in-frame insertion, or deletion or a canonical splice site variant; (2) a missense variant with a Grantham score $\geq$80 (range: 5–215), CADD_PHRED $\geq$15 (range: 0–99) or PhyloP $\geq$2.7; or (3) a putative splice-modulating variant with two out of four SpliceAI Δ scores $\geq$0.1 or one SpliceAI Δ score $\geq$0.15 (default settings, range 0–1).[20] Copy number variants (CNVs) were called with control-FREEC and SVs were called with Manta Structural Variant Caller V.1.1.0.[21,22] CNVs and SVs were prioritized if they passed the internal quality filter as previously described[8]; had a high quality score ($\geq$100 of 1,000); had an AF $\leq$ 1% in databases, such as gnomAD, Decipher, and GoNL; and affected at least one exon of *USH2A* or the aforementioned 62 arRP-associated or 17 USH-associated genes. Segregation analysis was performed if DNA samples of relatives were available.

Minigene splice assays

Variants predicted to have an effect on splicing (two out of four SpliceAI Δ scores $\geq$0.1 or one SpliceAI Δ score $\geq$0.15) were selected for a minigene splice assay. Four variants were selected prior to the release of SpliceAI and did not meet these criteria. Two of these variants (c.1644+7453A>G and c.4396+6885T>C) were included because an effect on pre-mRNA splicing was predicted by splice prediction tools incorporated in Alamut Visual Plus. Variant c.8710G>A was included due to its 1 base pair downstream location of c.8709C>T that was shown to have an effect on pre-mRNA splicing in a previous study,[9] whereas a fourth variant (c.6806-7599C>G) was selected as a unique variant absent from gnomAD in one of the first samples that was analyzed with

Table 1. An overview of all samples including identified potentially pathogenic *USH2A* variants

Study ID	Causal gene	Variant 1 cDNA	Protein effect variant 1	Variant 1 ACMG classification	Variant 2 cDNA	Protein effect variant 2	Variant 2 ACMG classification	Status of proband
**Solved and possibly solved**								
arRP1	*USH2A*	c.2299del	p.(Glu767Serfs*21)	Pathogenic	c.14134-5T>C	p.[=,Val4712Profs*2]	VUS	Possibly solved
arRP4[14]	*PQLC2*	c.596G>A	p.(Arg199Gln)	Likely pathogenic	c.596G>A	p.(Arg199Gln)	Likely pathogenic	Solved
arRP6	*EYS*	c.2527G>A	p.(Gly843Arg)	Likely pathogenic	c.-12233_-447-18280del	p.(?)	VUS	Possibly solved
arRP8	*USH2A*	c.11864G>A	p.(Trp3955*)	Pathogenic	c.9949C>T	p.(Arg3317Cys)	VUS	Possibly solved
arRP10	*USH2A*	c.10817T>C	p.(Leu3606Pro)	VUS	c.5573-19A>G	p.Gly1858_Thr1925del	Likely pathogenic	Possibly solved
arRP11	*USH2A*	c.1679del	p.(Pro560Leufs*31)	Pathogenic	c.9371+1G>C	p.(?)	Pathogenic	Solved
arRP19	*USH2A*	c.8559-2A>G	p.(?)	Pathogenic	c.13335_13343del	p.(Glu4445_Met4447del)	Likely pathogenic	Solved
arRP20	*USH2A*	c.12574C>T	p.(Arg4192Cys)	Likely pathogenic	c.4627+25436_4987+659del	p.(?)	Likely pathogenic	Solved
arRP21	*USH2A*	c.5573-1G>T	p.(?)	Likely pathogenic	c.2303G>A	p.[Cys768Tyr,Cys766Tyrfs*3, Glu767_Gly937del]	VUS	Possibly solved
arRP22	*USH2A*	c.10712C>T	p.(Thr3571Met)	Likely pathogenic	c.12575G>A	p.(Arg4192His)	Pathogenic	Solved
arRP24	*USH2A*	c.11105G>A	p.(Trp3702*)	Pathogenic	c.9433C>T	p.(Leu3145Phe)	VUS	Possibly solved
arRP26	*USH2A*	c.14803C>T	p.(Arg4935*)	Pathogenic	c.12575G>A	p.(Arg4192His)	Pathogenic	Solved
arRP29	*PROM1*	c.1301+2T>C	p.(?)	Pathogenic	c.2131-695A>C	p.(?)	VUS	Possibly solved
arRP34[6]	*USH2A*	c.2276G>T	p.(Cys759Phe)	Pathogenic	c.2276G>T	p.(Cys759Phe)	Pathogenic	Solved
arRP36	*USH2A*	c.2276G>T	p.(Cys759Phe)	Pathogenic	c.4397-3890A>G	p.Ala1465_ Ala1466ins*5	Likely pathogenic	Solved^
arRP37[15]	*RPE65*	c.886dup	p.(Arg296Lysfs*7)	Pathogenic	c.675C>A	p.(Asp215Valfs*4)	Pathogenic	Solved^
arRP41[6]	*USH2A*	c.2276G>T	p.(Cys759Phe)	Pathogenic	c.2276G>T	p.(Cys759Phe)	Pathogenic	Solved
arRP42[6]	*USH2A*	c.2276G>T	p.(Cys759Phe)	Pathogenic	c.2276G>T	p.(Cys759Phe)	Pathogenic	Solved
DFNB2	*USH2A*	c.653T>A	p.(Val218Glu)	Pathogenic	c.4627+25436_4987+659del	p.(?)	Likely pathogenic	Solved^
USH5	*USH2A*	c.1267G>T	p.(Gly423*)	Likely pathogenic	c.14791+5G>T	p.Tyr4862Alafs*22	Likely pathogenic	Solved
USH7	*USH2A*	c.13245_13246del	p.(Gly4416Valfs*2)	Likely pathogenic	c.652-23899_2809+1417dup	p.(?)	Pathogenic	Solved
USH8	*USH2A*	c.1823G>A	p.(Cys608Tyr)	Likely pathogenic	c.8655_8681+1681del	p.(?)	Likely pathogenic	Solved
USH10	*USH2A*	c.12067-2A>G	p.(?)	Pathogenic	c.4628-22994_2652del	p.(?)	Pathogenic	Solved
USH11	*PEX6*	c.2245G>A	p.(Gly749Ser)	Likely pathogenic	c.1802G>A	p.(Arg601Gln)	Likely pathogenic	Solved^
USH12	*USH2A*	c.2299del	p.(Glu767Serfs*21)	Pathogenic	c.9259-9T>A	p.Val3087Phefs*4	Likely pathogenic	Solved^
USH13	*USH2A*	c.11864G>A	p.(Trp3955*)	Pathogenic	c.5775A>T	p.Gly1858_Thr1925del	Likely pathogenic	Solved^

(*Continued on next page*)

Table 1. ***Continued***

Study ID	Causal gene	Variant 1 cDNA	Protein effect variant 1	Variant 1 ACMG classification	Variant 2 cDNA	Protein effect variant 2	Variant 2 ACMG classification	Status of proband
USH16	*USH2A*	c.7244C>G	p.(Ser2415*)	Pathogenic	c.7595-2144A>G	p.Lys2532Thrfs*56	Pathogenic	Solved
USH17	*USH2A*	c.2299del	p.(Glu767Serfs*21)	Pathogenic	c.15063_15081delinsGC	p.(Thr5022Glnfs*150)	Pathogenic	Solved
USH19	*USH2A*	c.8740C>T	p.(Arg2914*)	Pathogenic	c.9371+1G>C	p.(?)	Pathogenic	Solved
USH20[16]	*ARSG*	c.588C>A	p.(Tyr196*)	Pathogenic	c.705-3940_982+2952del	p.(Ser235Argfs*29)	Pathogenic	Solvedˆ
USH22	*USH2A*	c.3187_3188del	p.(Gln1063Serfs*15)	Pathogenic	c.1841-2A>G	p.(?)	Pathogenic	Solved
USH23	*USH2A*	c.802G>C	p.(Gly268Arg)	Likely pathogenic	c.14583-26A>G	p.[=,Tyr4862Alafs*22]	VUS	Possibly solved
USH24	*USH2A*	c.15089C>A	p.(Ser5030*)	Pathogenic	c.5573-834A>G	p.(?)	Likely pathogenic	Solved
USH25	*MYO7A*	c.2282+1G>A	p.(?)	Likely pathogenic	c.5482_5485del	p.(Tyr1828Alafs*50)	Likely pathogenic	Solved
USH26	*USH2A*	c.11864G>A	p.(Trp3955*)	Pathogenic	c.9258+1261_9371+1513del	p.(?)	Pathogenic	Solved
USH28	*USH2A*	c.2299del	p.(Glu767Serfs*21)	Pathogenic	c.3251_4627+31194del	p.(?)	Likely pathogenic	Solved
USH29	*USH2A*	c.2610C>A	p.(Cys870*)	Pathogenic	c.4133T>C	p.(Leu1378Pro)	VUS	Possibly solved
USH31	*USH2A*	c.11549-1G>A	p.(?)	Pathogenic	c.2994-3030_14343+488dup	p.(?)	Pathogenic	Solved
USH32	*USH2A*	c.14408T>C	p.(Ile4803Thr)	VUS	c.4885+375A>G[15]	p.Ser1629Valfs*52	Likely pathogenic	Possibly solved
USH33	*USH2A*	c.14791+2T>C	p.(?)	Likely pathogenic	c.9335_9371+8063delinsGAAGACACTCC	p.(?)	Pathogenic	Solved
USH34	*USH2A*	c.10561T>C	p.(Trp3521Arg)	Pathogenic	c.785-6636_1840+208del	p.(?)	Likely pathogenic	Solved
USH35[16]	*ARSG*	c.1326del	p.(Ser443Alafs*12)	Pathogenic	c.1024C>T	p.(Arg342Trp)	Likely pathogenic	Solved
USH37	*USH2A*	c.2299del	p.(Glu767Serfs*21)	Pathogenic	c.9258G>T	p.[Arg3037_Val3087del, Val3049*]	Likely pathogenic	Solvedˆ
USH39	*USH2A*	c.2299del	p.(Glu767Serfs*21)	Pathogenic	c.2311G>T	p.(Glu771*)	Likely pathogenic	Solvedˆ
USH41	*USH2A*	c.14525C>A	p.(Ser4842*)	Likely pathogenic	c.4397-3890A>G	p.Ala1465_Ala1466ins*5	Likely pathogenic	Solvedˆ
USH42[17]	*USH2A*	c.2299del	p.(Glu767Serfs*21)	Pathogenic	g.209815568_215637482inv	p.(?)	Pathogenic	Solved
USH44[17]	*USH2A*	c.9258+2601_9371+1539del	p.(?)	Pathogenic	g.42320825_215677220delins42320846_215677215inv	p.(?)	Pathogenic	Solvedˆ
USH46	*USH2A*	c.9959-3C>G	p.[Met3321Asnfs*22, =,Gly3320_Ser3338del]	VUS	c.1551-504C>T	p.Arg517_Cys518ins*13	Likely pathogenic	Possibly solvedˆ
USH49	*USH2A*	c.2299del	p.(Glu767Serfs*21)	Pathogenic	c.1841-377A>G	p.[Thr613_Gly614ins*9, =]	VUS	Possibly solved

(*Continued on next page*)

Table 1. *Continued*

Study ID	Causal gene	Variant 1 cDNA	Protein effect variant 1	Variant 1 ACMG classification	Variant 2 cDNA	Protein effect variant 2	Variant 2 ACMG classification	Status of proband
**Unsolved**								
arRP2		–	–	–	–	–	–	Unsolved
arRP3		c.2299del	p.(Glu767Serfs*21)	Pathogenic	–	–	–	Unsolved
arRP5		c.15433G>A	p.(Val5145Ile)	Benign	–	–	–	Unsolved
arRP7		c.10931C>T	p.(Thr3644Met)	VUS	c.9975G>A	p.(Gly3325=)	VUS	Unsolved
arRP9		c.6240G>T	p.(Lys2080Asn)	Benign	–	–	–	Unsolved
arRP12		c.[2276G>T(;)4618G>A]	p.(Cys759Phe(;)Asp1540Asn)	Pathogenic	-	-		Unsolved
arRP13		c.3443C>T	p.(Pro1148Leu)	VUS	–	–	–	Unsolved
arRP14		c.2276G>T	p.(Cys759Phe)	Pathogenic	c.11687T>C	p.(Ile3896Thr)	VUS	Unsolved
arRP15		c.11864G>A	p.(Trp3955*)	Pathogenic	–	–	–	Unsolved
arRP16		c.4486C>A	p.(Pro1496Thr)	VUS	c.14664G>A	p.(Thr4888=)	Likely benign	Unsolved
arRP17		c.7939C>T	p.(Pro2647Ser)	VUS	–	–	–	Unsolved
arRP18		c.3309C>A	p.(Tyr1103*)	Pathogenic	–	–	–	Unsolved
arRP23		c.12295-3T>A	p.Thr4099Valfs*2	Pathogenic	–	–	–	Unsolved
arRP25		c.920_923dup	p.(His308Glnfs*16)	Pathogenic	c.6240G>T	p.(Lys2080Asn)	Benign	Unsolved
arRP27		c.2276G>T	p.(Cys759Phe)	Pathogenic	–	–	–	Unsolved
arRP28		c.3664G>A	p.(Ala1222Thr)	VUS	–	–	–	Unsolved
arRP30		c.6001C>T	p.(Arg2001Cys)	Benign	–	–	–	Unsolved
arRP31		c.1439T>C	p.(Val480Ala)	VUS	–	–	–	Unsolved
arRP32		c.4732C>T	p.(Arg1578Cys)	Likely pathogenic	–	–	–	Unsolved
arRP33		c.14333C>A; 15377T>C	p.(Ala4778Asp;Ile5126Thr)	Benign	–	–	–	Unsolved
arRP35		c.5858C>G	p.(Ala1953Gly)	VUS	–	–	–	Unsolved
arRP38		c.12343C>T	p.(Arg4115Cys)	VUS	–	–	–	Unsolved
arRP39		c.2522C>A	p.(Ser841Tyr)	Benign	–	–	–	Unsolved
arRP40		–	–	–	–	–	–	Unsolved
arRP43		c.9258G>T	p.[Arg3037_Val3087del, Val3049*]	Likely pathogenic	–	–	–	Unsolved
arRP44		c.11597C>T	p.(Ala3866Val)	Likely benign	–	–	–	Unsolved
arRP45		–	–	–	–	–	–	Unsolved

(*Continued on next page*)

Table 1. ***Continued***

Study ID	Causal gene	Variant 1 cDNA	Protein effect variant 1	Variant 1 ACMG classification	Variant 2 cDNA	Protein effect variant 2	Variant 2 ACMG classification	Status of proband
arRP46		–	–	–	–	–	–	Unsolved
arRP47		c.8710G>A	p.(Val2904Ile)	VUS	–	–	–	Unsolved
CRD1		–	–	–	–	–	–	Unsolved
DFNB1		c.11864G>A	p.(Trp3955*)	Pathogenic	–	–	–	Unsolved
DFNB3		c.11927C>T	p.(Thr3976Met)	VUS	–	–	–	Unsolved
USH1		c.14020A>G	p.(Arg4674Gly)	Likely pathogenic	–	–	–	Unsolved
USH2		c.2920G>A	p.(Asp974Asn)	Likely benign	–	–	–	Unsolved
USH3		c.7594+250G>T	p.(?)	Benign	–	–	–	Unsolved
USH4		c.5780A>G	p.(Tyr1927Cys)	VUS	–	–	–	Unsolved
USH6		c.6670G>T	p.(Gly2224Cys)	VUS	c.1644+7453A>G	p.=	VUS	Unsolved
USH9		c.8167C>T	p.(Arg2723*)	Pathogenic	–	–	–	Unsolved
USH14		-	-	–	–	–	–	Unsolved
USH15		c.15433G>A	p.(Val5145Ile)	Benign	c.14753C>T	p.(Thr4918Met)	Likely benign	Unsolved
USH18		–	–	–	–	–	–	Unsolved
USH21		–	–	–	–	–	–	Unsolved
USH27		c.1522G>A	p.(Ala508Thr)	VUS	–	–	–	Unsolved
USH30		c.2299del	p.(Glu767Serfs*21)	Pathogenic	–	–	–	Unsolved
USH36		c.11864G>A	p.(Trp3955*)	Pathogenic	–	–	–	Unsolved
USH38		–	–	–	–	–	–	Unsolved
USH40		c.5516T>A	p.(Val1839Glu)	VUS	–	–	–	Unsolved
USH43		–	–	–	–	–	–	Unsolved
USH45		–	–	–	–	–	–	Unsolved
USH47		c.1439T>C	p.(Val480Ala)	VUS	–	–	–	Unsolved
USH48		–	–	–	–	–	–	Unsolved

Underlined variants are newly identified variants with WGS in this study. Probands for which segregation analysis confirmed the variants to be *in trans* are marked with an ˆ in the ’status of the proband’-column. Reference sequences (hg19):, *ARSG:* NM_014960.5, *EYS:* NM_001142800.2,*MYO7A:* NM_000260.4, *PEX6:* NM_000287.4, *PQLC2*: NM_001040125.2, *PROM1:* NM_006017.3, *USH2A*: NM_206933.4. arRP, autosomal recessive retinitis pigmentosa; USH, Usher syndrome; VUS, variant of uncertain significance. All variants are *USH2A* variants, unless another causal gene is listed.

WGS. Selected variants were either deep-intronic variants, non-canonical splice site variants, missense variants, or variants that affected a "branchpoint A" upstream of an exon. Constructs were generated with Gateway cloning technology (Thermo Fisher Scientific, Carlsbad, CA, USA) and minigene splice assays were performed as published previously.[23] Primer sequences are listed in Table S3.

Variant classification

SNVs were classified according to the American College of Medical Genetics (ACMG) guidelines with Franklin and SVs were manually classified according to published ACMG guidelines.[24,25] SNVs with an effect on pre-mRNA splicing were classified based on their effect in the minigene splice assay; variants with a full stop gain effect in all transcripts were classified as pathogenic, variants resulting in an in-frame deletion thereby disrupting a predicted functional protein domain were classified as likely pathogenic and variants with remaining conventionally spliced transcript were classified as variant of uncertain significance (VUS). Subjects with two (likely) pathogenic variants were considered genetically solved, subjects with one (likely) pathogenic variant and one VUS were considered "possibly solved."

Antisense oligonucleotides

AONs were designed according to previously published guidelines[26,27] and purchased from Eurogentec (Seraing, Belgium) with 2′-O-MOE modifications and a complete phosphorothioate backbone. For each PE, at least two AONs were designed complementary to splice acceptor or donor sites, or exonic splice enhancer regions. Only one AON targeting the PE resulting from c.4885+375A>G could be designed due to low GC-content of the PE sequence. NCBI blast searches were performed to exclude potential off-target binding sites for each designed AON. Moreover, a scrambled oligonucleotide (SON) as well as an AON containing three mismatches compared with the most potent AON for each target (3ntMM AON) were designed and purchased. All AONs were dissolved in sterile phosphate buffered saline (PBS 1x) to a stock concentration of 1 mM and diluted in PBS to concentrations of 0.01 mM, 0.02 mM, 0.05 mM, and 0.1 mM. One microliter of each AON was co-transfected in duplicate with individual minigene splice assay plasmids containing the variant of interest, as described in the minigene splices assays section. Transfected cells were subjected to RNA analyses as described before.[23]

Generation of photoreceptor precursor cells

Blood was obtained from subjects arRP36 (heterozygous for c.4397-3890A>G), USH46 (heterozygous for c.1551-504C>T), and a healthy control. Peripheral blood mononuclear cells were isolated and reprogrammed into induced pluripotent stem cells (iPSCs) through episomal nucleofection, as described previously,[28] and subsequently differentiated into photoreceptor precursor cells (PPCs).[17,29] iPSC lines were generated at the Stem Cell Technology Center of the Radboudumc (Nijmegen, the Netherlands). AONs or a SON were gymnotically delivered to the PPCs on day 28 by adding them directly to the culture medium. Cycloheximide (CHX, in a final concentration of 100 μg/mL) was added on day 29 and cells were harvested in sterile PBS on day 30. Levels of PE inclusion and reversal after treatment were determined with PCR and RT-qPCR (in duplicate) according to standard protocols (primer sequences in Table S4). AON efficacy was assessed based on the splicing correction capacity at the different AON concentrations.

Results

Forty-one probands were (likely) solved having biallelic *USH2A* variants

One hundred unrelated subjects were included for WGS analysis. Forty-seven of these subjects were clinically diagnosed with non-syndromic arRP, 49 with USH, three with DFNB, and one with CRD and hearing loss. The subjects with DFNB had one (likely) pathogenic *USH2A* variant and were still in the first or second decade of life, potentially not (yet) presenting early signs of vision loss.

In total, 6,054 unique SNVs were identified within *USH2A* and predicted regulatory elements. Of these, 2,820 variants had a gnomAD AF ≤1% in the general population. Ninety-seven variants were considered potentially pathogenic, as they met our inclusion criteria for being a stop gain variant, frameshift variant, in-frame insertion or deletion, a canonical splice site variant, or a potentially pathogenic missense variant or putative splice-modulating variant (Table S5). Nineteen of these SNVs were not reported in ClinVar or the Leiden Open (source) Variation Database (LOVD) and were therefore considered novel variants in our study. Twelve *USH2A* SVs on 13 alleles adhered to our criteria, including two duplications, two inversions,[16] and eight deletions of which one (c.4627+25436_4987+659del) was identified in two probands. The largest SV with both breakpoints within *USH2A* was a duplication from exon 15 to 65 (c.2994-3030_14343+488dup), the shortest SV was a deletion of exon 43 (c.8655_8681+1681del). All SVs were confirmed with PCR and subsequent Sanger sequencing. After assessment of all individual variants from the total cohort, 41 cases were genetically explained with (likely) biallelic *USH2A* variants. An overview of all variants that were classified as (likely) pathogenic or were VUS that were deemed potentially causative is presented in Table 1.

All samples were also evaluated for the presence of potentially pathogenic variants in other arRP- or USH-associated genes (Table S2), applying the same criteria to detect biallelic variants in these genes. We identified 133 unique variants in these genes, resulting in eight of the remaining probands being (possibly) solved with biallelic variants in the following genes: *ARSG* (two cases, described in Velde et al.[14]), *EYS, MYO7A, PEX6, PQCL2* (described in Millo et al.[15]), *PROM1,* and *RPE65* (described in Panneman et al.[30]). An overview of solve rates is visualized in Figure 1.

Assessment of the effect of variants on splicing using minigene splice assays

We identified 36 variants with two out of four SpliceAI Δ scores ≥0.1 or one SpliceAI Δ score ≥0.15 (Table S6).[20] Stop gain variants (n = 2) and canonical splice site variants (n = 7) were excluded from these analyses as they are

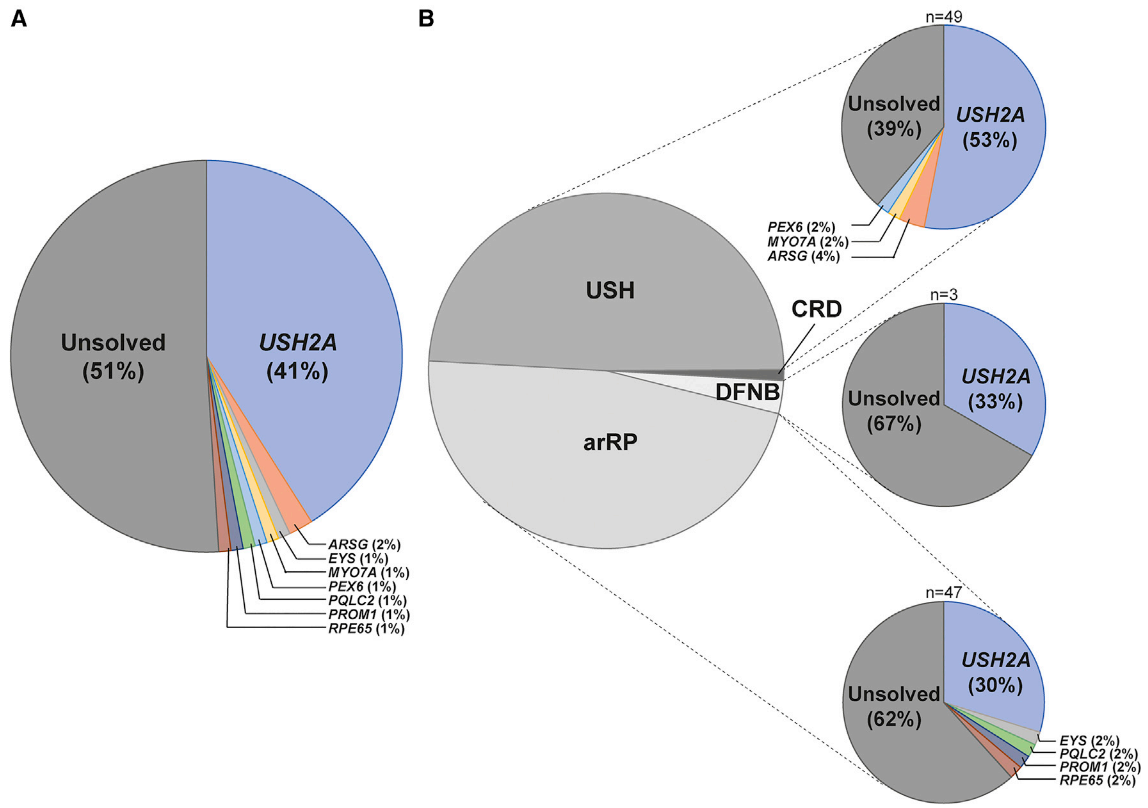

Figure 1. Overview of causative genes in a cohort of 100 USH and arRP-affected individuals
(A) In 49 of the 100 probands, (likely) biallelic (likely) pathogenic variants were identified in genes associated with autosomal recessive retinitis pigmentosa (arRP) or Usher syndrome (USH).
(B) The solved rate per phenotype (arRP, deafness [DFNB] or USH) is shown. The majority (61%) of USH subjects are genetically explained, compared with 38% of arRP subjects and one out of three DFNB affected individuals. The remaining subject (in dark gray) was diagnosed with cone-rod dystrophy (CRD) with hearing loss and remained unsolved.

generally considered to be pathogenic. For three variants (c.5573-834A>G, c.7595-2144A>G, and c.12295-3T>A) a deleterious effect on *USH2A* pre-mRNA splicing was already previously determined[1,2,31] and they were therefore omitted for testing. We also excluded one variant that was an artifact and confirmed to be absent with Sanger sequencing (c.1551-508C>T), one variant in a proband that would remain mono-allelic after performance of a minigene splice assay (c.4732C>T), and one variant for which gain of canonical splice sites was predicted (c.9975G>A) (Table S6). Minigene splice assays were performed for the remaining 21 variants (Figures 2A–2D, Table S7). Thirteen of these variants showed a(n) (partial) effect on splicing (Figure S1), of which eight variants affected pre-mRNA splicing in all amplified transcripts and these were therefore classified as (likely) pathogenic. The remaining five variants (c.1841-377A>G, c.2303G>A, c.9959-3C>G, c.14134-5T>C, and c.14583-26A>G) only revealed a partial effect on splicing and were therefore classified as VUS.

(a) Deep-intronic variants (n = 7)

Seven of the 21 variants that were evaluated in a minigene splice assay were deep-intronic *USH2A* variants (Figure 2A). Four of seven tested deep-intronic variants were shown to have an effect on pre-mRNA splicing. Variant c.1551-504C>T induced the inclusion of a 118 nt PE (PE8; chr1:216495824–216495941) in intron 8, resulting in premature termination of protein translation (p.Arg517_Cys518ins*13). Variant c.4397-3890A>G resulted in the inclusion of an 87 nt PE (PE20; chr1:216352719–216352805) in intron 20, containing an in-frame stop codon (p.Ala1465_Ala1466ins*5). Variant c.4885+375A>G was already recently described.[32] We confirmed inclusion of a 130 nt PE (PE23; chr1:216261985–216262114) in intron 23 (p.Ser1629Valfs*52). A deep-intronic variant located in intron 10, c.1841-377A>G (in individual USH50), resulted in the inclusion of a 94 nt PE in part of the transcripts (PE10; chr1:216463130–216463223) (p.[Thr613_Gly614ins*9,=]). Variants c.1644+7453A>G, c.4396+6885T>C and c.6806-7599C>G (identified *in cis* with c.2276G>T) did not reveal an effect on pre-mRNA splicing.

(b) Variants in non-canonical splice site region (n = 6)

Six *USH2A* non-canonical splice site variants were identified (Figure 2B). Two variants resulted in the complete skipping of either exon 28 (c.5775A>T; p.Gly1858_Thr1925del)

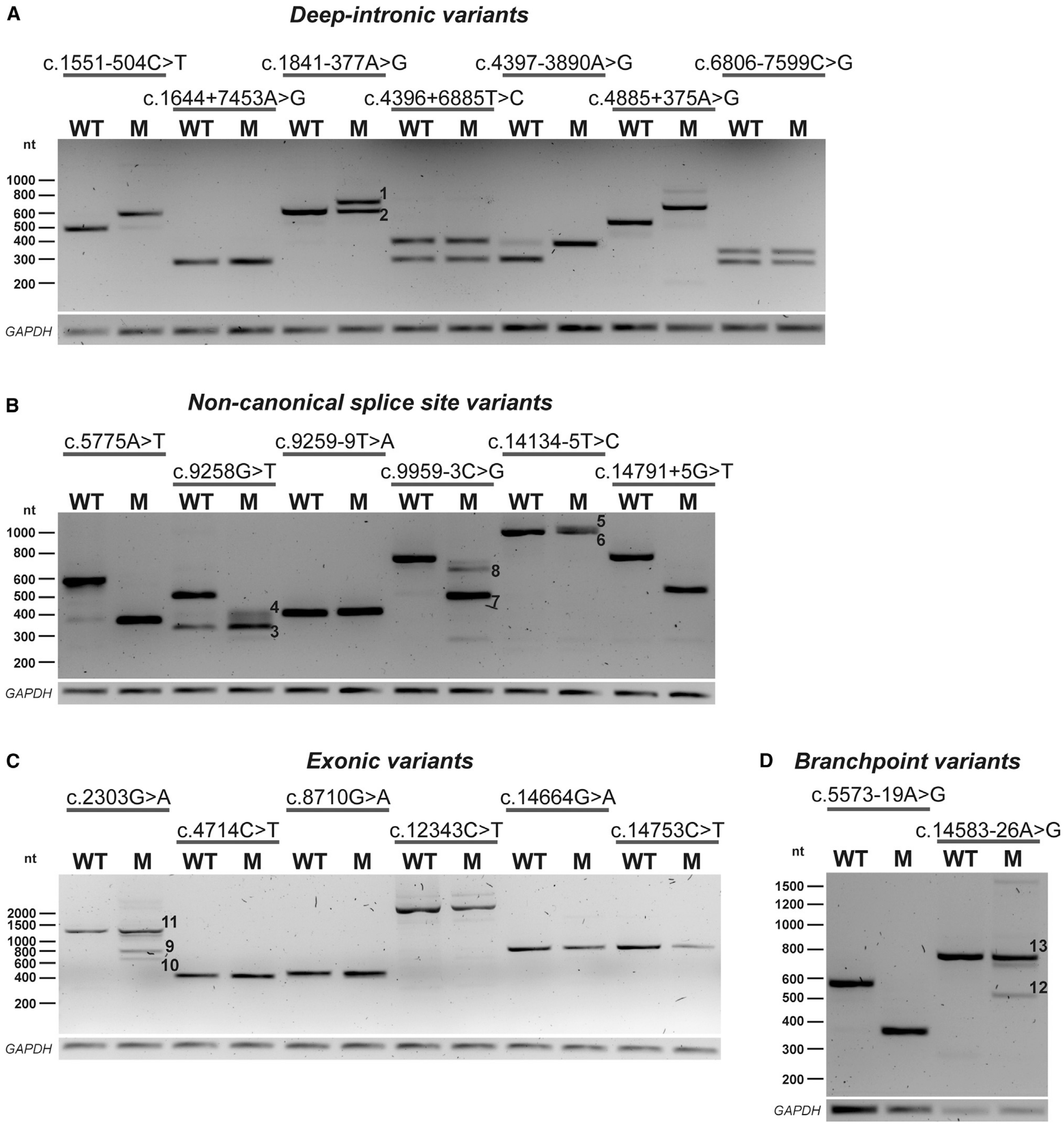

Figure 2. Minigene splice assays for 21 variants with a potential effect on splicing

Representative gel images from minigene splice assays for deep-intronic variants, non-canonical splice site variants, exonic variants, and branchpoint variants. The numbers visualized on the gel correspond to the protein effects described below.

(A) Three deep-intronic variants (c.1551-504C>T, c.4397-3890A>G, and c.4885+375A>G[32]) resulted in PE inclusion in all amplified transcripts. Variant c.1841-377A>G, resulted in the inclusion of a 94 nt PE (1) and remaining conventionally spliced transcript (2).

(B) Two non-canonical splice site variants (c.5775A>T and c.14791+5G>T) resulted in the complete skipping of exon 28 or exon 67 in all detected transcripts, whereas variant c.9259-9T>A resulted in a 7-nt extension. Variant c.9258G>T was shown to have a dual effect: partial skipping of exon 46 (3) and alternative partial skipping of exon 46 (4). Likewise, variant c.14134-5T>C resulted in a 47-nt extension upstream of exon 65 (5) and remaining conventionally spliced transcript (6). Variant c.9959-3C>G induced two alternative pre-mRNA splicing events: skipping of complete exon 51 (7) and partial skipping of exon 51 (8) with incomplete penetrance.

(C) Variant c.2303G>A had multiple effects, with part of the transcripts lacking the center region of exon 13 (9), part lacking the 3′ side of exon 13 (10) and part being conventionally spliced mRNA (11).

(D) Variant c.5573-19A>G resulted in skipping of exon 28 in all transcripts, whereas variant c.14583-26A>G resulted in skipping of exon 67 (12) and conventionally spliced mRNA (13). Transfections were performed in duplicate, *GAPDH* was used as loading control in all experiments. An overview of the observed effects on pre-mRNA splicing and the consequences on protein level is listed in Table S7. M, variant of interest; nt, nucleotides; WT, wild type.

or exon 67 (c.14791+5G>T; p.Tyr4862Alafs*22) in all detected transcripts, whereas variant c.9259-9T>A resulted in a 7-nt extension upstream of exon 47 (p.Val3087Phefs*4). Variant c.9258G>T was shown to have a dual effect; partial skipping of exon 46 (Δ153 nt) in part of the transcripts combined with an alternative partial skipping of exon 46 (Δ116 nt) in the remaining transcripts, as a consequence of the use of another alternative splice donor site in exon 46 (p.[Arg3037_Val3087del,Val3049*]). Likewise, variant c.14134-5T>C resulted in a 47-nt extension upstream of exon 65 in part of the transcripts (p.[=,Val4712Profs*2]). Variant c.9959-3C>G induced multiple alternative pre-mRNA splicing events; skipping of complete exon 51 and partial skipping of exon 51 (Δ57 nt) (p.[Met3321Asnfs*22,=,Gly3320_Ser3338del]), with incomplete penetrance.

(c) Exonic variants (n = 6)

The potential effect on splicing of six exonic variants outside of canonical splice site regions was evaluated (Figure 2C). Variant c.2303G>A (p.(Cys768Tyr)) had multiple effects on pre-mRNA splicing, with part of the transcripts lacking the center region of exon 13, leaving the canonical splice sites intact and with use of a novel donor splice site 129 nt downstream of the canonical splice acceptor site and a novel splice acceptor site 115 nt upstream of the canonical splice donor site. Other transcripts lacked 513 nt at the 3′ side of exon 13 or were conventionally spliced mRNA (p.[Cys766Tyrfs*3,Glu767_Gly937del,Cys768Tyr]). The other five exonic variants did not show an effect on pre-mRNA splicing using the corresponding minigene splice assays, i.e., c.4714C>T (*in cis* with c.2299del), c.8710G>A, c.12343C>T, c.14664G>A, and c.14753C>T.

(d) Branchpoint variants (n = 2)

Two variants (c.5573-19A>G, c.14583-26A>G, Figure 2D) were predicted to affect the adenine in a branch sequence ("branchpoint A") upstream of exons 28 and 67, respectively. A minigene splice assay revealed that variant c.5573-19A>G resulted in skipping of exon 28 (p.Gly1858_Thr1925del) in all transcripts, while variant c.14583-26A>G resulted in skipping of exon 67 and conventionally spliced mRNA (p.[=,Tyr4862Alafs*22]).

Design and evaluation of antisense oligonucleotides to correct aberrant pre-mRNA splicing events

AONs and controls were designed and evaluated for their potential to correct aberrant splicing induced by four deep-intronic variants resulting in PE inclusion (c.1551-504C>T, c.1841-377A>G, c.4397-3890A>G, and c.4885+375A>G). Characteristics of all AONs and their position relative to the targeted PE and exonic splice enhancers are described in Table S8 and Figure S2.

For all four targets, at least one of the AONs was capable of redirecting aberrant splicing at the lowest concentration (0.01 μM) using the matching minigene splice assay vectors (Figure 3). Transfection with an SON did not show effect for three of the four targets, which indicates that the splicing correction is sequence-specific. To further confirm the sequence-specificity, 3ntMM AONs were also assessed for their potential to redirect splicing in the corresponding minigene splice assays. For c.1551-504C>T and c.4397-3890A>G, these 3ntMM AONs were indeed less effective than the original AONs, even at the highest concentration used (0.1 μM), corroborating the effect of the original AON to be sequence-specific. In contrast, no difference in efficiency of splicing redirection was observed between the original and 3ntMM AONs against c.1841-377A>G (PE10) and c.4885+375A>G (PE23) at all tested concentrations. The SON of PE23 also redirected splicing. Therefore, it is unclear whether or not the effect of the designed AON to correct aberrant PE23 splicing is sequence-specific.

AONs are effective in patient-derived PPCs

PPCs were generated from iPSCs derived from two subjects (arRP36 and USH46) who were confirmed compound heterozygous for c.4397-3890A>G (PE20) and c.2276G>T (p.(Cys759Phe)) and c.1551-504C>T (PE8) and c.9959-3C>G (p.[Met3321Asnfs*22,=,Gly3320_Ser3338del]), respectively. These PPCs enabled us to confirm the PE inclusion caused by these variants and to test the AONs in a cellular model resembling human photoreceptors within the entire genetic context of the patient. RT-qPCR analyses of several neuronal progenitor, photoreceptor, and retinal pigment epithelium markers indicated that the differentiation of iPSCs into PPCs was successful for both cell lines (Figure S3).

As expected, PE inclusion was observed in PPCs derived from subjects having the c.1551-504C>T (PE8) or c.4397-3890A>G (PE20) variant (Figure 4). For variant c.4397-3890A>G, AON2 was effective in redirecting aberrant splicing at all three tested concentrations, while the 3ntMM AON and SON did not show any effect. This effect was confirmed with RT-qPCR as a reduction of 49% (after treatment with 0.01 μM AON) to 92% (treated with 0.1 μM AON) of transcripts containing PE20 as compared with the control sample (treated with CHX, but not with AON) was observed. Interestingly, inclusion of an additional PE (PE20a) was observed in the untreated samples and samples that were treated with 3ntMM AON and SON, but not in the samples treated with AON2. This PE20a is possibly a result of variant c.4396+7263del and is assumed to be part of the c.4397-3890A>G haplotype because it was detected with WGS in both subjects having the c.4397-3890A>G variant.

For variant c.1551-504C>T, AON3 redirected aberrant *USH2A* pre-mRNA splicing, albeit with a lower efficiency than was observed in the minigene splice assay, as still remaining PE8-containing transcripts could be detected after AON treatment in all tested concentrations. Compared with the amount of transcript that contains PE8 in the control sample that was not treated with AON, a reduction

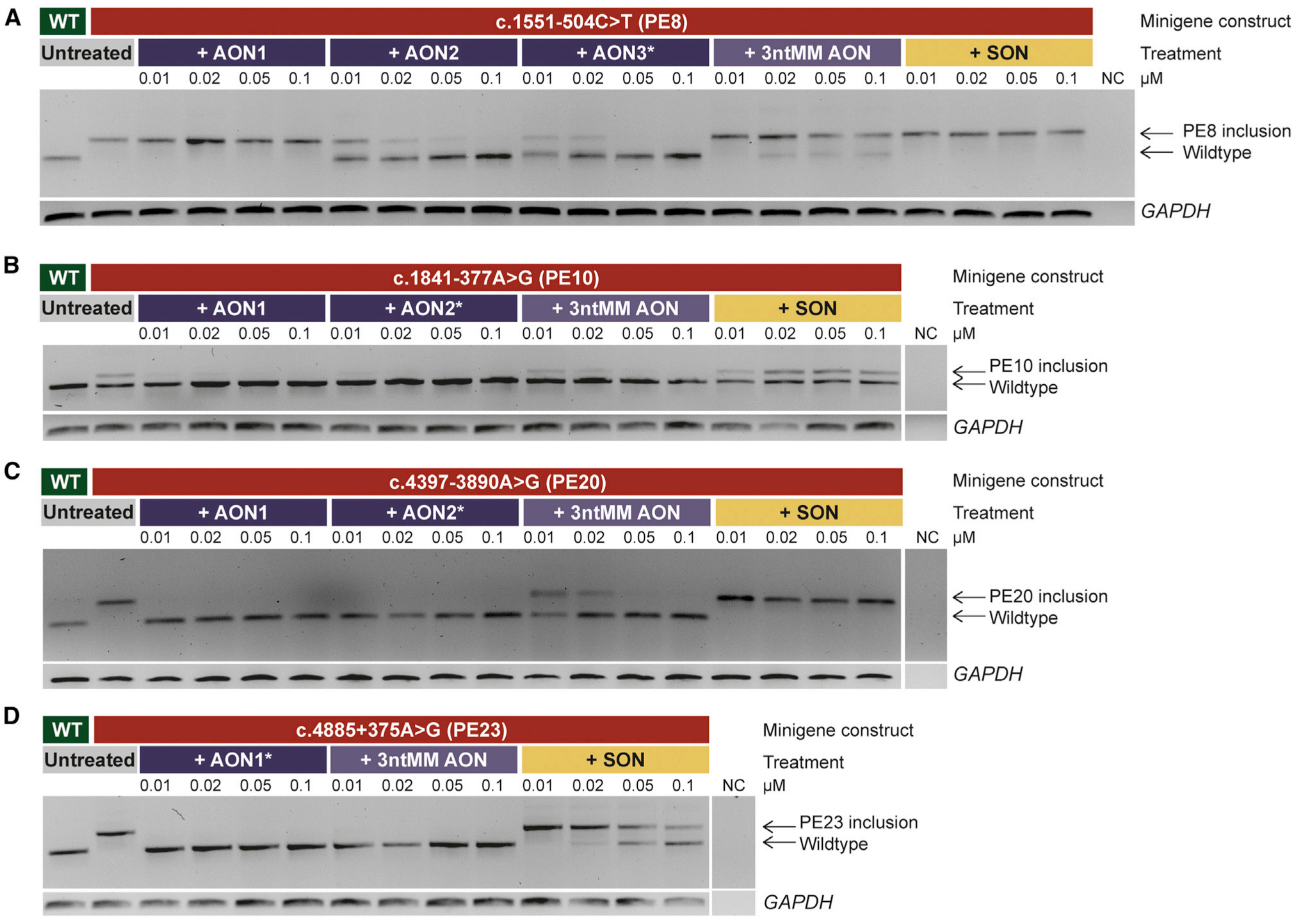

Figure 3. Antisense oligonucleotide (AON) treatment dilution series for four deep-intronic targets
Representative gel images from AON treatment studies in minigenes.
(A) Two out of three AONs (AON2 and AON3) were effective in reverting a 118-nt pseudoexon (PE) inclusion in intron 8, caused by variant c.1551-504C>T, for all tested concentrations. A three-nucleotide mismatch antisense oligonucleotide (3ntMM AON) of the most potent AON (AON3, marked with an asterisk) and a scrambled oligonucleotide (SON), had minimal to no effect on reverting PE inclusion.
(B) Variant c.1841-377A>G resulted in partial inclusion of a 94-nt PE in intron 10. Both AONs efficiently reverted PE inclusion, while a 3ntMM AON of AON2 had reduced effect and an SON was not effective.
(C) Variant c.4397-3890A>G caused inclusion of an 87-nt PE in intron 20. Both AONs efficiently reverted PE inclusion, while a 3ntMM AON of AON2 had reduced effect and an SON was not effective.
(D) Variant c.4885+375A>G causes inclusion of a 130-nt PE in intron 23. The AON that could be designed for the region was efficient in reverting PE inclusion. A 3ntMM AON was fully effective as well and an SON had reduced effect. NC, negative control; nt, nucleotides; WT, wild type. Transfections were performed in duplicate, *GAPDH* was used as loading control in all experiments.

of 33% (treated with 0.1 µM AON) to 50% (treated with 1 µM AON) of PE8-containing transcript was observed.

Discussion

Through our study, 49 out of 100 (49%) previously unsolved arRP and (suspected) USH individuals now received a conclusive or possibly conclusive genetic diagnosis. Our analysis confirmed that WGS is a powerful method for solving cases that remained genetically unsolved after screening the *USH2A* exons and flanking intronic regions. Using the combination of WGS and subsequent dedicated minigene splice assays, we identified novel pathogenic deep-intronic variants and SVs and confirmed a splice defect for other exonic, branchpoint and non-canonical splice site variants. WGS followed by testing of splice defects should therefore be considered as a valuable method to increase genetic solve rates, which is especially important in the current era of upcoming genetic therapies and increased focus on personalized medicine. Furthermore, we showed that AONs are a valuable tool to correct aberrant splicing as a consequence of deep-intronic variants in *USH2A*.

To compare the efficacy of our WGS analysis with published WGS studies is complex, due to varying prescreening conditions and inclusion criteria. However, the solve rate in our study seems in line with other WGS studies that include *USH2A* variants. A WGS study from the United Kingdom solved 31% of persons with IRDs that did not receive a complete genetic diagnosis after WES and 55% of cases that were directly screened with

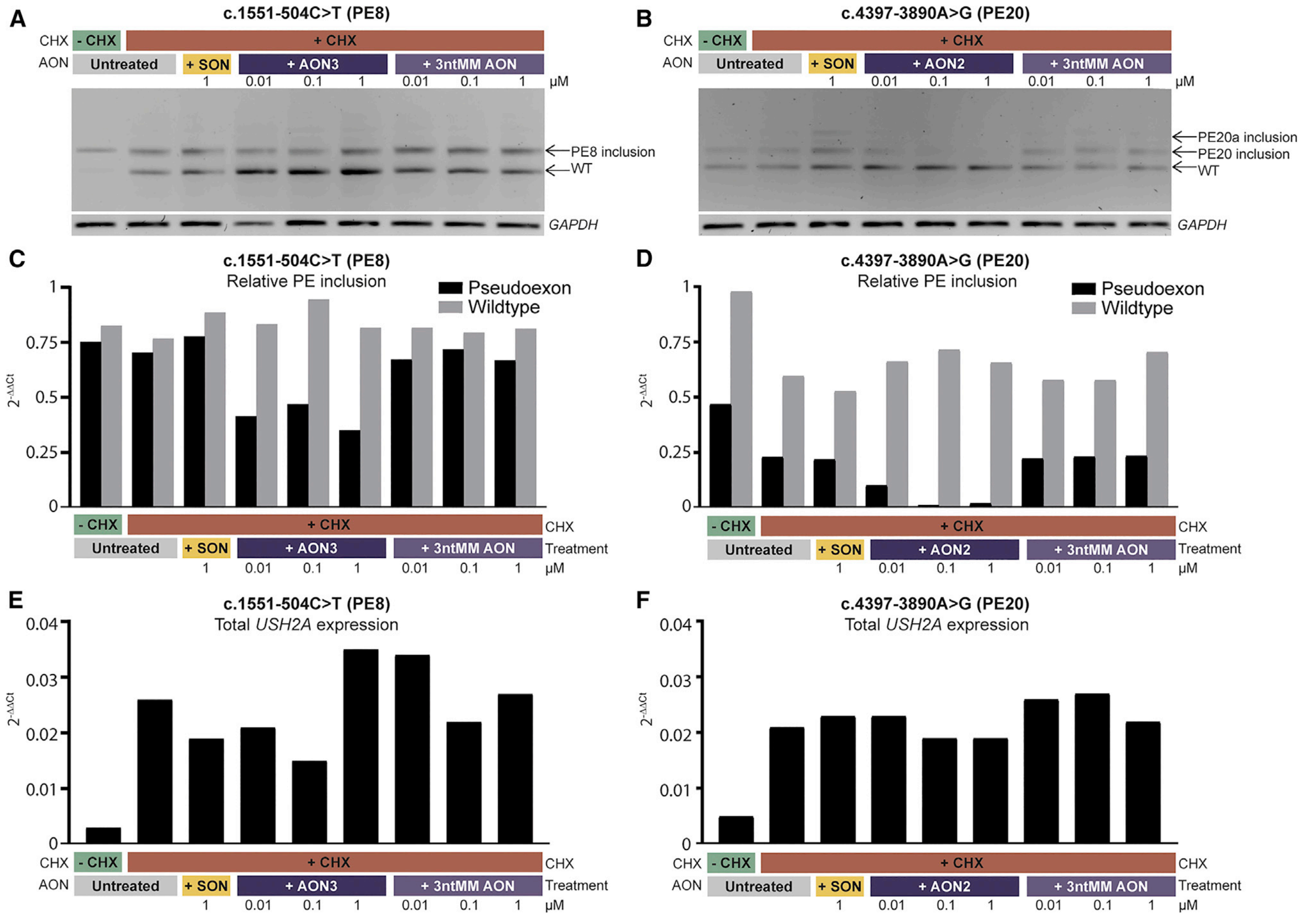

Figure 4. AON treatment of photoreceptor precursor cells (PPCs) differentiated from patient-derived induced pluripotent stem cells (iPSCs)

(A) RT-PCR on mRNA of PPC samples that were heterozygous for c.1551-504C>T and subsequent gel electrophoresis showed that ~50% of transcripts harbor a pseudoexon (PE), while 50% are wild-type (WT) transcripts. Treatment with three different concentrations of antisense oligonucleotide 3 (AON3) against PE8 for 48 h resulted in a reduction, but not complete reversion of PE inclusion. Treatment with a scrambled oligonucleotide (SON) or 3-nucleotide mismatch AON (3ntMM AON) did not result in reduction of PE inclusion. *GAPDH* was used as loading control.

(B) In PPCs heterozygous for c.4397-3890A>G, inclusion of PE20 was fully reverted after treatment with AON2 against PE20, while an SON and 3ntMM AON did not have an effect.

(C and D) The effect observed with RT-PCR was confirmed with RT-qPCR. Levels of transcripts containing the PE of interest as well as WT transcript was compared with *USH2A* exon 13 expression (normalized against *GUSB*) and confirmed that AON3 against PE8 can partially rescue PE8 inclusion, while AON2 against PE20 has a full effect.

(E and F) Expression of *USH2A* (exon 13) compared with *GUSB*. *USH2A* expression was relatively low in all samples; however, lowest in samples not treated with cycloheximide (CHX).

WGS.[4] In another study in which 57 mono-allelic IRD cases from the Netherlands, Ireland, and Israel were included that were previously screened with WES or target-capture sequencing, the solve rate was 21% (12 of 57).[8] In our study, we solved 49% of cases that underwent prior testing using WES or targeted sequencing, such as molecular inversion probe-based sequencing. We identified several variants that are not (easily) being detected by WES or targeted sequencing, such as pathogenic deep-intronic *USH2A* variants (in seven cases) or SVs (13 alleles in 12 cases). SVs have been previously shown to explain a portion of missing heritability in cases that were screened with targeted sequencing panels.[33] The detection of 13 (likely) pathogenic *USH2A* SVs in our cohort of 100 subjects, representing 11% of (likely) pathogenic variants identified within *USH2A*, emphasized the value of WGS compared with targeted sequencing panels and WES for a substantial increase in diagnostic yield for IRDs.

Of the solved probands, 22 subjects had SNVs that could have been detected through WES or targeted sequencing. This was likely to be partially caused by lack of detection due to incomplete screening with targeted sequencing. However, nine variants resulted in deviations in pre-mRNA splicing in a minigene splice assay and were located either in an exon or in a non-canonical splice site region. Previous reports estimated that up to a third of disease-causing variants disrupt pre-mRNA splicing, either directly or through mis-regulation of splice-regulating factors.[34] Our results emphasize that increased knowledge on variant interpretation and functional testing of possible

effects on splicing of branchpoint variants, missense variants, synonymous variants, and non-canonical splice site variants will increase diagnostic yield in *USH2A* screening, and in genetic testing for hereditary disorders in general. Re-evaluation of targeted sequencing and WES data of currently still unsolved cases with a particular focus on splice-modulating properties is therefore recommended.

The genetic diagnosis for 13 subjects could be completed after performing a minigene splice assay for variants that were selected based on our criteria for SpliceAI. Eight of these variants displayed an effect on all detected transcripts in the corresponding minigene splice assays (Figure S1 and Table S7). To our knowledge, the c.5573-19A>G variant, reported previously as VUS in both LOVD and ClinVar, is one of the first suggested branchpoint variants within *USH2A* resulting in a pathogenic effect.[35] The identification of an additional potential branchpoint variant c.14583-26A>G (p.[=,Tyr4862Alafs*22]) stresses the importance of including the evaluation of branchpoint variants in genetic screening efforts. Five variants that were tested in a minigene splice assay showed residual conventionally spliced transcripts along with a deleterious effect on splicing and they were therefore classified as VUS.

Recent studies have been published on specificity and sensitivity of splice prediction tools.[36,37] In a study by Rowlands et al.,[36] SpliceAI (suggested Δ score ≥0.2) outperformed other splice prediction tools, although false positive and false negative calls were observed. In general, our findings are in line with the findings of Rowlands et al. that SpliceAI is able to adequately predict effects on pre-mRNA splicing. Only four of the 17 variants (24%) that met our inclusion criteria (two out of four SpliceAI Δ scores ≥0.1 or one Δ score ≥0.15) did not display an effect on pre-mRNA splicing in HEK293T cells, which were all exonic variants. Four other variants, including one exonic variant, that were tested in this study did not meet our SpliceAI inclusion criteria, but for none of these variants an effect on pre-mRNA splicing was observed. The inclusion criteria in our study were less restrictive compared with those used by Rowlands et al. and Riepe et al. (≥0.2),[36,37] yielding three additional variants (c.1841-377A>G, c.5573-19A>G, and c.5775A>T) with a maximum SpliceAI Δ score between 0.1 and 0.2, which did show an effect on pre-mRNA splicing. We would therefore suggest using 0.1 as a criterion for SpliceAI for deep-intronic, non-canonical splice site and branchpoint variants, but this could be adapted for exonic variants as no effect on pre-mRNA splicing was observed for the majority of exonic variants that we tested. A SpliceAI criterion of ≥0.2 for exonic variants outside the non-canonical splice site regions would exclude all exonic variants that did not have an effect on pre-mRNA splicing in our minigene splice assays but would yield the remaining variant (c.2303G>A) that did have an effect. The use of other splice prediction tools in addition to SpliceAI could also be considered for this type of variants.

Screening of variants with a potential effect on pre-mRNA splicing in a broader genetic context, such as larger minigenes or use of patient-derived PPCs, may result in alternative outcomes. In addition, previous research has shown that recognition of splice sites can be cell type specific,[38] and the splicing patterns observed in our minigene splice assay may deviate from the natural situation in the human retina and inner ear. However, these variants have effect on *USH2A* pre-mRNA splicing and were all identified in arRP and USH subjects with a previously identified mono-allelic pathogenic *USH2A* variant, suggesting that additional functional evidence may determine that these variants are indeed (likely) pathogenic according to ACMG guidelines. Furthermore, similar studies performed for another IRD gene, *ABCA4*, have shown that variants creating new splice sites are recapitulated in patient-derived cells, and only those creating splicing enhancers may show tissue-dependent specificity.[38] Another important point is that little is known about the minimal amount of remaining wild-type transcript that would be required to retain normal retinal function. Functional data derived from the in-frame skipping of zebrafish *ush2a* exon 13 suggests that as little as ~20% of functional transcripts could be enough to retain retinal function.[11] Future research in cellular and animal models will shed light on this question, potentially resulting in the reclassification of variants with an incomplete effect on splicing.

We were able to obtain patient material from two individuals carrying deep-intronic variants that was subsequently used to generate iPSCs to differentiate them to PPCs. Using these cells we could validate our findings in the minigene system. Unexpectedly, we identified an additional PE (PE20a; chr1:216356236–216356337), incorporated in the mRNA derived from cultured PPCs of subject arRP36 with variant c.4397-3890A>G (PE20). PE20a was only identified in samples that were treated with CHX, and treatment with AON against PE20 also appeared to revert inclusion of PE20a. The presence of an additional PE could be an artifact of our assay; however, this stresses the importance of considering an entire haplotype when designing a therapeutic strategy and the importance of using large sequence parts flanking the variant of interest, preferably including flanking exons, in dedicated minigene splice assays.

We also identified variants and splicing defects in *USH2A* exon 13. Currently a phase II/III clinical trial to induce exon 13 skipping is ongoing. Therefore, this information is very valuable for patients, as they might be eligible to receive future treatments. Although mutations in exon 13 are relatively recurrent, this is also important for individuals with ultra-rare or even unique pathogenic variants. The impact of Milasen, an AON customized for a single subject suffering from Batten disease, has led to (inter)national initiatives to develop treatments for those ultra-rare cases with no commercial interest.[39] Highlighting once again the importance of having a concise genetic

diagnosis in order to become eligible for future therapeutic genetic interventions.

Unfortunately, we were unable to provide 51 of 100 probands a solid genetic diagnosis. In most of these cases, one (likely) pathogenic *USH2A* variant is present, and therefore another pathogenic *USH2A* variant might well reside *in trans*. However, in the general population, approximately 36% of individuals are healthy carriers of a pathogenic variant in an IRD-associated gene.[40] While we screened for causative coding and non-coding variants in other genes associated with arRP or USH, by chance, subjects in our cohort may have a pathogenic variant in *USH2A*, with the underlying genetic cause of their disease elsewhere. In fact, eight cases in our cohort were solved after identification of biallelic variants in IRD genes other than *USH2A*. Furthermore, SVs could remain undetected or unrecognized, due to the nature of short-read sequencing techniques.[16,41,42] Pathogenic variants may also reside in low coverage regions, in genes associated with disorders that are phenotypically overlapping, or in genes not yet associated with IRDs. We did not observe potentially pathogenic variants in 10 potential regulatory regions of the *USH2A* gene[6] nor in the known retinal disease modifier *PDZD7*.[19] Improved knowledge on *USH2A* regulatory elements, potential modifiers, being either protective or negative, or potentially digenic inheritance patterns is likely to result in more precise classification of variants in the future.

In conclusion, we confirmed that our strategy, with elaborate WGS screening and thorough variant interpretation and thorough assessment of splice defects associated with all types of SNVs, is an effective method to significantly improve diagnostic solve rates. As a result of our strategy, we (possibly) solved 49% (49 of 100) of genetically unexplained probands with suspected *USH2A*-associated disease. In 21 subjects, we identified (likely) pathogenic variants in *USH2A* exon 13 of whom 14 subjects received a definite genetic diagnosis and these are now considered eligible for receiving *Ultevursen*/QR-421a-mediated exon 13 skipping therapy when available.[11] Moreover, we identified three novel pathogenic deep-intronic variants that result in the inclusion of a PE and we were able to develop effective AONs that redirected aberrant pre-mRNA splicing. Collectively, our experimental approach has been effective in the identification of novel, potentially treatable, disease-causing variants in *USH2A* and can be applied to the diagnostic pipelines for all inherited disorders known so far.

Data and code availability

Data are available upon request. Pathogenic variant data are uploaded to the Leiden Open (source) Variation Database (LOVD: https://databases.lovd.nl/shared/genes/USH2A). All other whole genome sequencing data are subject to controlled access because these may compromise research participant privacy. These data may become available upon a data transfer agreement approved by the local ethics committee and can be obtained from corresponding author S.R. upon reasonable request.

Supplemental information

Supplemental information can be found online at https://doi.org/10.1016/j.xhgg.2023.100181.

Acknowledgments

The work of J.R. was funded by the Velux Stiftung (Project number: 1129 to H.K., S.R., F.P.M.C., and E.v.W.). The work of S.R. is funded by the Foundation Fighting Blindness-career development award (CD-GE-0621-0809-RAD to S.R.). The research was also supported by the Algemene Nederlandse Vereniging ter voorkoming van Blindheid, Oogfonds, Landelijke Stichting voor Blinden en Slechtzienden, Rotterdamse Stichting Blindenbelangen, Stichting Blindenhulp, Stichting tot Verbetering van het Lot der Blinden, and Stichting Blinden-Penning (to S.R. and F.P.M.C.). The work of S.E.d.B. was funded by the European Union's Horizon 2020 Research and Innovation Program under the EJP RD COFUND-EJP N° 825575 (to F.P.M.C. and S.R.). The work of P.L. and L.D. was funded by EJP RD Solve-RET (EJPRD19-234) and GAUK (136121).

We thank all individuals who participated in this study. We thank the Radboud Stem Cell Technology Center for the generation of iPSCs and the Radboud Technology Center Genomics for their assistance with whole genome sequencing. We also thank Saskia van der Velde-Visser and Marlie Jacobs-Camps for DNA sample registration and administration.

Declaration of interests

The authors declare no competing interests.

Received: October 11, 2022
Accepted: January 12, 2023

Web resources

ClinVar: https://www.ncbi.nlm.nih.gov/clinvar/
DECIPHER: https://www.deciphergenomics.org/
Franklin: https://franklin.genoox.com/clinical-db/home
gnomAD SV: https://gnomad.broadinstitute.org/gene/ENSG00000042781?dataset=gnomad_sv_r2_1
gnomAD SNV: https://gnomad.broadinstitute.org/gene/ENSG00000042781?dataset=gnomad_r3
GoNL: https://www.nlgenome.nl/
LOVD: https://databases.lovd.nl/shared/genes/USH2A
RetNet: https://sph.uth.edu/retnet/disease.htm

References

1. Vaché, C., Besnard, T., le Berre, P., García-García, G., Baux, D., Larrieu, L., Abadie, C., Blanchet, C., Bolz, H.J., Millan, J., et al. (2012). Usher syndrome type 2 caused by activation of an USH2A pseudoexon: implications for diagnosis and therapy. Hum. Mutat. *33*, 104–108. https://doi.org/10.1002/humu.21634.
2. Liquori, A., Vaché, C., Baux, D., Blanchet, C., Hamel, C., Malcolm, S., Koenig, M., Claustres, M., and Roux, A.F. (2016). Whole USH2A gene sequencing identifies several new deep intronic mutations. Hum. Mutat. *37*, 184–193. https://doi.org/10.1002/humu.22926.

3. Baux, D., Vaché, C., Blanchet, C., Willems, M., Baudoin, C., Moclyn, M., Faugère, V., Touraine, R., Isidor, B., Dupin-Deguine, D., et al. (2017). Combined genetic approaches yield a 48% diagnostic rate in a large cohort of French hearing-impaired patients. Sci. Rep. *7*, 16783. https://doi.org/10.1038/s41598-017-16846-9.
4. Carss, K.J., Arno, G., Erwood, M., Stephens, J., Sanchis-Juan, A., Hull, S., Megy, K., Grozeva, D., Dewhurst, E., Malka, S., et al. (2017). Comprehensive rare variant analysis via whole-genome sequencing to determine the molecular pathology of inherited retinal disease. Am. J. Hum. Genet. *100*, 75–90. https://doi.org/10.1016/j.ajhg.2016.12.003.
5. de Bruijn, S.E., Fiorentino, A., Ottaviani, D., Fanucchi, S., Melo, U.S., Corral-Serrano, J.C., Mulders, T., Georgiou, M., Rivolta, C., Pontikos, N., et al. (2020). Structural variants create new topological-associated domains and ectopic retinal enhancer-gene contact in dominant retinitis pigmentosa. Am. J. Hum. Genet. *107*, 802–814. https://doi.org/10.1016/j.ajhg.2020.09.002.
6. Reurink, J., de Vrieze, E., Li, C.H.Z., van Berkel, E., Broekman, S., Aben, M., Peters, T., Oostrik, J., Neveling, K., Venselaar, H., et al. (2022). Scrutinizing pathogenicity of the USH2A c.2276 G > T; p.(Cys759Phe) variant. NPJ Genom. Med. *7*, 37. https://doi.org/10.1038/s41525-022-00306-z.
7. Ellingford, J.M., Barton, S., Bhaskar, S., Williams, S.G., Sergouniotis, P.I., O'Sullivan, J., Lamb, J.A., Perveen, R., Hall, G., Newman, W.G., et al. (2016). Whole genome sequencing increases molecular diagnostic yield compared with current diagnostic testing for inherited retinal disease. Ophthalmology *123*, 1143–1150. https://doi.org/10.1016/j.ophtha.2016.01.009.
8. Fadaie, Z., Whelan, L., Ben-Yosef, T., Dockery, A., Corradi, Z., Gilissen, C., Haer-Wigman, L., Corominas, J., Astuti, G.D.N., de Rooij, L., et al. (2021). Whole genome sequencing and in vitro splice assays reveal genetic causes for inherited retinal diseases. NPJ Genom. Med. *6*, 97. https://doi.org/10.1038/s41525-021-00261-1.
9. Reurink, J., Dockery, A., Oziębło, D., Farrar, G.J., Ołdak, M., Ten Brink, J.B., Bergen, A.A., Rinne, T., Yntema, H.G., Pennings, R.J.E., et al. (2021). Molecular inversion probe-based sequencing of USH2A exons and splice sites as a cost-effective screening tool in USH2 and arRP cases. Int. J. Mol. Sci. *22*, 6419. https://doi.org/10.3390/ijms22126419.
10. Toualbi, L., Toms, M., and Moosajee, M. (2020). USH2A-retinopathy: from genetics to therapeutics. Exp. Eye Res. *201*, 108330. https://doi.org/10.1016/j.exer.2020.108330.
11. Dulla, K., Slijkerman, R., van Diepen, H.C., Albert, S., Dona, M., Beumer, W., Turunen, J.J., Chan, H.L., Schulkens, I.A., Vorthoren, L., et al. (2021). Antisense oligonucleotide-based treatment of retinitis pigmentosa caused by USH2A exon 13 mutations. Mol. Ther. *29*, 2441–2455. https://doi.org/10.1016/j.ymthe.2021.04.024.
12. Fuster-García, C., García-García, G., González-Romero, E., Jaijo, T., Sequedo, M.D., Ayuso, C., Vázquez-Manrique, R.P., Millán, J.M., and Aller, E. (2017). USH2A gene editing using the CRISPR system. Mol. Ther. Nucleic Acids *8*, 529–541. https://doi.org/10.1016/j.omtn.2017.08.003.
13. Pendse, N.D., Lamas, V., Pawlyk, B.S., Maeder, M.L., Chen, Z.Y., Pierce, E.A., and Liu, Q. (2019). In vivo assessment of potential therapeutic approaches for USH2A-associated diseases. Adv. Exp. Med. Biol. *1185*, 91–96. https://doi.org/10.1007/978-3-030-27378-1_15.
14. Velde, H.M., Reurink, J., Held, S., Li, C.H.Z., Yzer, S., Oostrik, J., Weeda, J., Haer-Wigman, L., Yntema, H.G., Roosing, S., et al. (2022). Usher syndrome type IV: clinically and molecularly confirmed by novel ARSG variants. Hum. Genet. *141*, 1723–1738. https://doi.org/10.1007/s00439-022-02441-0.
15. Millo, T., Rivera, A., Obolensky, A., Marks-Ohana, D., Xu, M., Li, Y., Wilhelm, E., Gopalakrishnan, P., Gross, M., Rosin, B., et al. (2022). Identification of autosomal recessive novel genes and retinal phenotypes in members of the solute carrier (SLC) superfamily. Genet. Med. *24*, 1523–1535. https://doi.org/10.1016/j.gim.2022.03.020.
16. de Bruijn, S.E., Rodenburg, K., Corominas, J., Ben-Yosef, T., Reurink, J., Kremer, H., Whelan, L., Plomp, A.S., Berger, W., Farrar, G.J., et al. (2022). Optical genome mapping and revisiting short-read genome sequencing data reveal previously overlooked structural variants disrupting retinal disease-associated genes. Genet. Med., 100345.
17. Garanto, A., Duijkers, L., Tomkiewicz, T.Z., and Collin, R.W.J. (2019). Antisense oligonucleotide screening to optimize the rescue of the splicing defect caused by the recurrent deep-intronic ABCA4 variant c.4539+2001G>A in stargardt disease. Genes *10*, 452. https://doi.org/10.3390/genes10060452.
18. Farek, J., Hughes, D., Mansfield, A., Krasheninina, O., Nasser, W., Sedlazeck, F.J., et al. (2022). xAtlas: scalable small variant calling across heterogeneous next-generation sequencing experiments. Gigascience *12*. giac125. https://doi.org/10.1093/gigascience/giac125.
19. Ebermann, I., Phillips, J.B., Liebau, M.C., Koenekoop, R.K., Schermer, B., Lopez, I., Schäfer, E., Roux, A.F., Dafinger, C., Bernd, A., et al. (2010). PDZD7 is a modifier of retinal disease and a contributor to digenic Usher syndrome. J. Clin. Invest. *120*, 1812–1823. https://doi.org/10.1172/jci39715.
20. Jaganathan, K., Kyriazopoulou Panagiotopoulou, S., McRae, J.F., Darbandi, S.F., Knowles, D., Li, Y.I., Kosmicki, J.A., Arbelaez, J., Cui, W., Schwartz, G.B., et al. (2019). Predicting splicing from primary sequence with deep learning. Cell *176*, 535–548.e24. https://doi.org/10.1016/j.cell.2018.12.015.
21. Boeva, V., Popova, T., Bleakley, K., Chiche, P., Cappo, J., Schleiermacher, G., Janoueix-Lerosey, I., Delattre, O., and Barillot, E. (2012). Control-FREEC: a tool for assessing copy number and allelic content using next-generation sequencing data. Bioinformatics *28*, 423–425. https://doi.org/10.1093/bioinformatics/btr670.
22. Chen, X., Schulz-Trieglaff, O., Shaw, R., Barnes, B., Schlesinger, F., Källberg, M., Cox, A.J., Kruglyak, S., and Saunders, C.T. (2016). Manta: rapid detection of structural variants and indels for germline and cancer sequencing applications. Bioinformatics *32*, 1220–1222. https://doi.org/10.1093/bioinformatics/btv710.
23. Sangermano, R., Bax, N.M., Bauwens, M., van den Born, L.I., De Baere, E., Garanto, A., Collin, R.W.J., Goercharn-Ramlal, A.S.A., den Engelsman-van Dijk, A.H.A., Rohrschneider, K., et al. (2016). Photoreceptor progenitor mRNA analysis reveals exon skipping resulting from the ABCA4 c.5461-10T→C mutation in stargardt disease. Ophthalmology *123*, 1375–1385. https://doi.org/10.1016/j.ophtha.2016.01.053.
24. Abou Tayoun, A.N., Pesaran, T., DiStefano, M.T., Oza, A., Rehm, H.L., Biesecker, L.G., Harrison, S.M.; and ClinGen Sequence Variant Interpretation Working Group ClinGen SVI (2018). Recommendations for interpreting the loss of function PVS1 ACMG/AMP variant criterion. Hum. Mutat. *39*, 1517–1524. https://doi.org/10.1002/humu.23626.
25. Richards, S., Aziz, N., Bale, S., Bick, D., Das, S., Gastier-Foster, J., Grody, W.W., Hegde, M., Lyon, E., Spector, E., et al. (2015).

Standards and guidelines for the interpretation of sequence variants: a joint consensus recommendation of the American college of medical genetics and genomics and the association for molecular pathology. Genet. Med. *17*, 405–424. https://doi.org/10.1038/gim.2015.30.

26. Slijkerman, R., Kremer, H., and van Wijk, E. (2018). Antisense oligonucleotide design and evaluation of splice-modulating properties using cell-based assays. Methods Mol. Biol. *1828*, 519–530. https://doi.org/10.1007/978-1-4939-8651-4_34.
27. Aartsma-Rus, A. (2012). Overview on AON design. Methods Mol. Biol. *867*, 117–129. https://doi.org/10.1007/978-1-61779-767-5_8.
28. Koolen, L., Gagliardi, G., Ten Brink, S.C.A., de Breuk, A., Heesterbeek, T.J., Hoyng, C.B., Albert, S., and den Hollander, A.I. (2022). Generation and characterization of human induced pluripotent stem cells (iPSCs) from three patients with age-related macular degeneration carrying rare variants in the CFH gene. Stem Cell Res. *60*, 102669. https://doi.org/10.1016/j.scr.2022.102669.
29. Flamier, A., Barabino, A., and Bernier, G. (2016). Differentiation of human embryonic stem cells into cone photoreceptors. Bio-protocol *6*, e1870. https://doi.org/10.21769/BioProtoc.1870.
30. Panneman, D.M., Hitti-Malin, R.J., Holtes, L.K., de Bruijn, S.E., Reurink, J., Boonen, E.G., et al. (2023). Cost-effective sequence analysis of 113 genes in 1,192 probands with retinitis pigmentosa and Leber congenital amaurosis. Front. Cell Dev. Biol. https://doi.org/10.3389/fcell.2023.1112270.
31. Lenassi, E., Vincent, A., Li, Z., Saihan, Z., Coffey, A.J., Steele-Stallard, H.B., Moore, A.T., Steel, K.P., Luxon, L.M., Héon, E., et al. (2015). A detailed clinical and molecular survey of subjects with nonsyndromic USH2A retinopathy reveals an allelic hierarchy of disease-causing variants. Eur. J. Hum. Genet. *23*, 1318–1327. https://doi.org/10.1038/ejhg.2014.283.
32. Mansard, L., Baux, D., Vaché, C., Blanchet, C., Meunier, I., Willems, M., Faugère, V., Baudoin, C., Moclyn, M., Bianchi, J., et al. (2021). The study of a 231 French patient cohort significantly extends the mutational spectrum of the two major usher genes MYO7A and USH2A. Int. J. Mol. Sci. *22*, 13294. https://doi.org/10.3390/ijms222413294.
33. Van Schil, K., Naessens, S., Van de Sompele, S., Carron, M., Aslanidis, A., Van Cauwenbergh, C., Kathrin Mayer, A., Van Heetvelde, M., Bauwens, M., Verdin, H., et al. (2018). Mapping the genomic landscape of inherited retinal disease genes prioritizes genes prone to coding and noncoding copy-number variations. Genet. Med. *20*, 202–213. https://doi.org/10.1038/gim.2017.97.
34. Sibley, C.R., Blazquez, L., and Ule, J. (2016). Lessons from non-canonical splicing. Nat. Rev. Genet. *17*, 407–421. https://doi.org/10.1038/nrg.2016.46.
35. Le Guédard-Méreuze, S., Vaché, C., Baux, D., Faugère, V., Larrieu, L., Abadie, C., Janecke, A., Claustres, M., Roux, A.F., and Tuffery-Giraud, S. (2010). Ex vivo splicing assays of mutations at noncanonical positions of splice sites in USHER genes. Hum. Mutat. *31*, 347–355. https://doi.org/10.1002/humu.21193.
36. Rowlands, C., Thomas, H.B., Lord, J., Wai, H.A., Arno, G., Beaman, G., Sergouniotis, P., Gomes-Silva, B., Campbell, C., Gossan, N., et al. (2021). Comparison of in silico strategies to prioritize rare genomic variants impacting RNA splicing for the diagnosis of genomic disorders. Sci. Rep. *11*, 20607. https://doi.org/10.1038/s41598-021-99747-2.
37. Riepe, T.V., Khan, M., Roosing, S., Cremers, F.P.M., and t Hoen, P.A.C. (2021). Benchmarking deep learning splice prediction tools using functional splice assays. Hum. Mutat. *42*, 799–810. https://doi.org/10.1002/humu.24212.
38. Albert, S., Garanto, A., Sangermano, R., Khan, M., Bax, N.M., Hoyng, C.B., Zernant, J., Lee, W., Allikmets, R., Collin, R.W.J., and Cremers, F.P.M. (2018). Identification and rescue of splice defects caused by two neighboring deep-intronic ABCA4 mutations underlying stargardt disease. Am. J. Hum. Genet. *102*, 517–527. https://doi.org/10.1016/j.ajhg.2018.02.008.
39. Kim, J., Hu, C., Moufawad El Achkar, C., Black, L.E., Douville, J., Larson, A., Pendergast, M.K., Goldkind, S.F., Lee, E.A., Kuniholm, A., et al. (2019). Patient-customized oligonucleotide therapy for a rare genetic disease. N. Engl. J. Med. *381*, 1644–1652. https://doi.org/10.1056/NEJMoa1813279.
40. Hanany, M., Rivolta, C., and Sharon, D. (2020). Worldwide carrier frequency and genetic prevalence of autosomal recessive inherited retinal diseases. Proc. Natl. Acad. Sci. USA *117*, 2710–2716. https://doi.org/10.1073/pnas.1913179117.
41. Fadaie, Z., Neveling, K., Mantere, T., Derks, R., Haer-Wigman, L., den Ouden, A., Kwint, M., O'Gorman, L., Valkenburg, D., Hoyng, C.B., et al. (2021). Long-read technologies identify a hidden inverted duplication in a family with choroideremia. HGG Adv. *2*, 100046. https://doi.org/10.1016/j.xhgg.2021.100046.
42. Chaisson, M.J.P., Sanders, A.D., Zhao, X., Malhotra, A., Porubsky, D., Rausch, T., Gardner, E.J., Rodriguez, O.L., Guo, L., Collins, R.L., et al. (2019). Multi-platform discovery of haplotype-resolved structural variation in human genomes. Nat. Commun. *10*, 1784. https://doi.org/10.1038/s41467-018-08148-z.

HGGA, Volume *4*

Supplemental information

Whole genome sequencing for *USH2A*-associated disease reveals several pathogenic deep-intronic variants that are amenable to splice correction

Janine Reurink, Nicole Weisschuh, Alejandro Garanto, Adrian Dockery, L. Ingeborgh van den Born, Isabelle Fajardy, Lonneke Haer-Wigman, Susanne Kohl, Bernd Wissinger, G. Jane Farrar, Tamar Ben-Yosef, Fatma Kivrak Pfiffner, Wolfgang Berger, Marianna E. Weener, Lubica Dudakova, Petra Liskova, Dror Sharon, Manar Salameh, Ashley Offenheim, Elise Heon, Giorgia Girotto, Paolo Gasparini, Anna Morgan, Arthur A. Bergen, Jacoline B. ten Brink, Caroline C.W. Klaver, Lisbeth Tranebjærg, Nanna D. Rendtorff, Sascha Vermeer, Jeroen J. Smits, Ronald J.E. Pennings, Marco Aben, Jaap Oostrik, Galuh D.N. Astuti, Jordi Corominas Galbany, Hester Y. Kroes, Milan Phan, Wendy A.G. van Zelst-Stams, Alberta A.H.J. Thiadens, Joke B.G.M. Verheij, Mary J. van Schooneveld, Suzanne E. de Bruijn, Catherina H.Z. Li, Carel B. Hoyng, Christian Gilissen, Lisenka E.L.M. Vissers, Frans P.M. Cremers, Hannie Kremer, Erwin van Wijk, and Susanne Roosing

Table of Contents

Figure S1: Effects on pre-mRNA splicing observed in minigene splice assays for thirteen variants....... 2

Figure S2: Position of antisense oligonucleotides (AONs) and 3 nucleotide mismatch AONs (3ntMM AONs) relative to their targets. 4

Figure S3: Differentiation efficiency of patient-derived photoreceptor precursor cells (PPCs). 5

Table S1: Pre-screening methods of all samples 6

Table S2: All genes associated with Usher(-like) syndrome and autosomal recessive retinitis pigmentosa that were assessed in genome sequencing data of 100 cases. 9

Table S3: Sequences of primers used to generate constructs for the minigene splice assays. 10

Table S4: Sequences of primers used in qPCR. 11

Table S5*: All *USH2A* variants that met our variant inclusion criteria in 100 cases. 12

Table S6*: All *USH2A* variants with a predicted effect on splicing. 13

Table S7: Overview of observed effects on pre-mRNA splicing and consequences on protein level of 21 variants that were tested with minigene splice assays. 14

Table S8: Characteristics of all oligonucleotides designed in this study 15

Supplemental references 16

* Table S5 and Table S6 are separate excel files

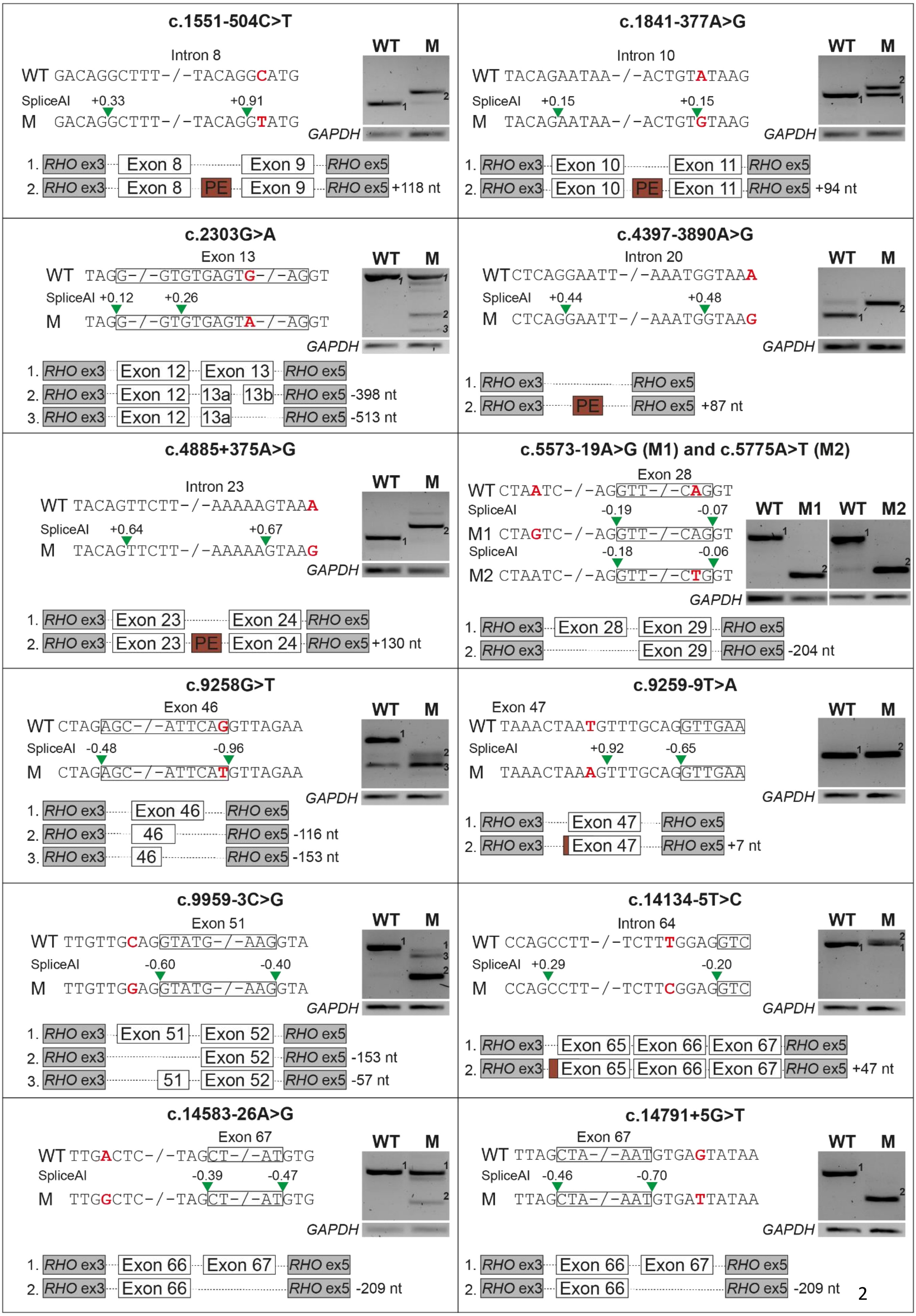
c.1551-504C>T
Intron 8
WT GACAGGCTTT-/-TACAGGCATG
SpliceAI +0.33 +0.91
M GACAGGCTTT-/-TACAGGTATG
WT M
GAPDH
1. RHO ex3 Exon 8 Exon 9 RHO ex5
2. RHO ex3 Exon 8 PE Exon 9 RHO ex5 +118 nt
c.1841-377A>G
Intron 10
WT TACAGAATAA-/-ACTGTATAAG
SpliceAI +0.15 +0.15
M TACAGAATAA-/-ACTGTGTAAG
WT M
GAPDH
1. RHO ex3 Exon 10 Exon 11 RHO ex5
2. RHO ex3 Exon 10 PE Exon 11 RHO ex5 +94 nt
c.2303G>A
Exon 13
WT TAGG-/-GTGTGAGTG-/-AGGT
SpliceAI +0.12 +0.26
M TAGG-/-GTGTGAGTA-/-AGGT
WT M
GAPDH
1. RHO ex3 Exon 12 Exon 13 RHO ex5
2. RHO ex3 Exon 12 13a 13b RHO ex5 -398 nt
3. RHO ex3 Exon 12 13a RHO ex5 -513 nt
c.4397-3890A>G
Intron 20
WT CTCAGGAATT-/-AAATGGTAAA
SpliceAI +0.44 +0.48
M CTCAGGAATT-/-AAATGGTAAG
WT M
GAPDH
1. RHO ex3 RHO ex5
2. RHO ex3 PE RHO ex5 +87 nt
c.4885+375A>G
Intron 23
WT TACAGTTCTT-/-AAAAAGTAAA
SpliceAI +0.64 +0.67
M TACAGTTCTT-/-AAAAAGTAAG
WT M
GAPDH
1. RHO ex3 Exon 23 Exon 24 RHO ex5
2. RHO ex3 Exon 23 PE Exon 24 RHO ex5 +130 nt
c.5573-19A>G (M1) and c.5775A>T (M2)
Exon 28
WT CTAATC-/-AGGTT-/-CAGGT
SpliceAI -0.19 -0.07
M1 CTAGTC-/-AGGTT-/-CAGGT
SpliceAI -0.18 -0.06
M2 CTAATC-/-AGGTT-/-CTGGT
WT M1 WT M2
GAPDH
1. RHO ex3 Exon 28 Exon 29 RHO ex5
2. RHO ex3 Exon 29 RHO ex5 -204 nt
c.9258G>T
Exon 46
WT CTAGAGC-/-ATTCAGGTTAGAA
SpliceAI -0.48 -0.96
M CTAGAGC-/-ATTCATGTTAGAA
WT M
GAPDH
1. RHO ex3 Exon 46 RHO ex5
2. RHO ex3 46 RHO ex5 -116 nt
3. RHO ex3 46 RHO ex5 -153 nt
c.9259-9T>A
Exon 47
WT TAAACTAATGTTTGCAGGTTGAA
SpliceAI +0.92 -0.65
M TAAACTAAAGTTTGCAGGTTGAA
WT M
GAPDH
1. RHO ex3 Exon 47 RHO ex5
2. RHO ex3 Exon 47 RHO ex5 +7 nt
c.9959-3C>G
Exon 51
WT TTGTTGCAGGTATG-/-AAGGTA
SpliceAI -0.60 -0.40
M TTGTTGGAGGTATG-/-AAGGTA
WT M
GAPDH
1. RHO ex3 Exon 51 Exon 52 RHO ex5
2. RHO ex3 Exon 52 RHO ex5 -153 nt
3. RHO ex3 51 Exon 52 RHO ex5 -57 nt
c.14134-5T>C
Intron 64
WT CCAGCCTT-/-TCTTTGGAGGTC
SpliceAI +0.29 -0.20
M CCAGCCTT-/-TCTTCGGAGGTC
WT M
GAPDH
1. RHO ex3 Exon 65 Exon 66 Exon 67 RHO ex5
2. RHO ex3 Exon 65 Exon 66 Exon 67 RHO ex5 +47 nt
c.14583-26A>G
Exon 67
WT TTGACTC-/-TAGCT-/-ATGTG
SpliceAI -0.39 -0.47
M TTGGCTC-/-TAGCT-/-ATGTG
WT M
GAPDH
1. RHO ex3 Exon 66 Exon 67 RHO ex5
2. RHO ex3 Exon 66 RHO ex5 -209 nt
c.14791+5G>T
Exon 67
WT TTAGCTA-/-AATGTGAGTATAA
SpliceAI -0.46 -0.70
M TTAGCTA-/-AATGTGATTATAA
WT M
GAPDH
1. RHO ex3 Exon 66 Exon 67 RHO ex5
2. RHO ex3 Exon 66 RHO ex5 -209 nt

Figure S1: Effects on pre-mRNA splicing observed in minigene splice assays for thirteen variants. Thirteen of the 21 tested variants revealed differences in pre-mRNA splicing between mutant (M) and wildtype (WT) constructs in our minigene splice assays. The effects observed after RT-PCR are shown as well as a schematic representation of these effects including the *RHO* exons 3 and 5 that are flanking the *USH2A* genomic region cloned in the minigene. Splice predictions were obtained with SpliceAI. nt: nucleotides PE: pseudoexon

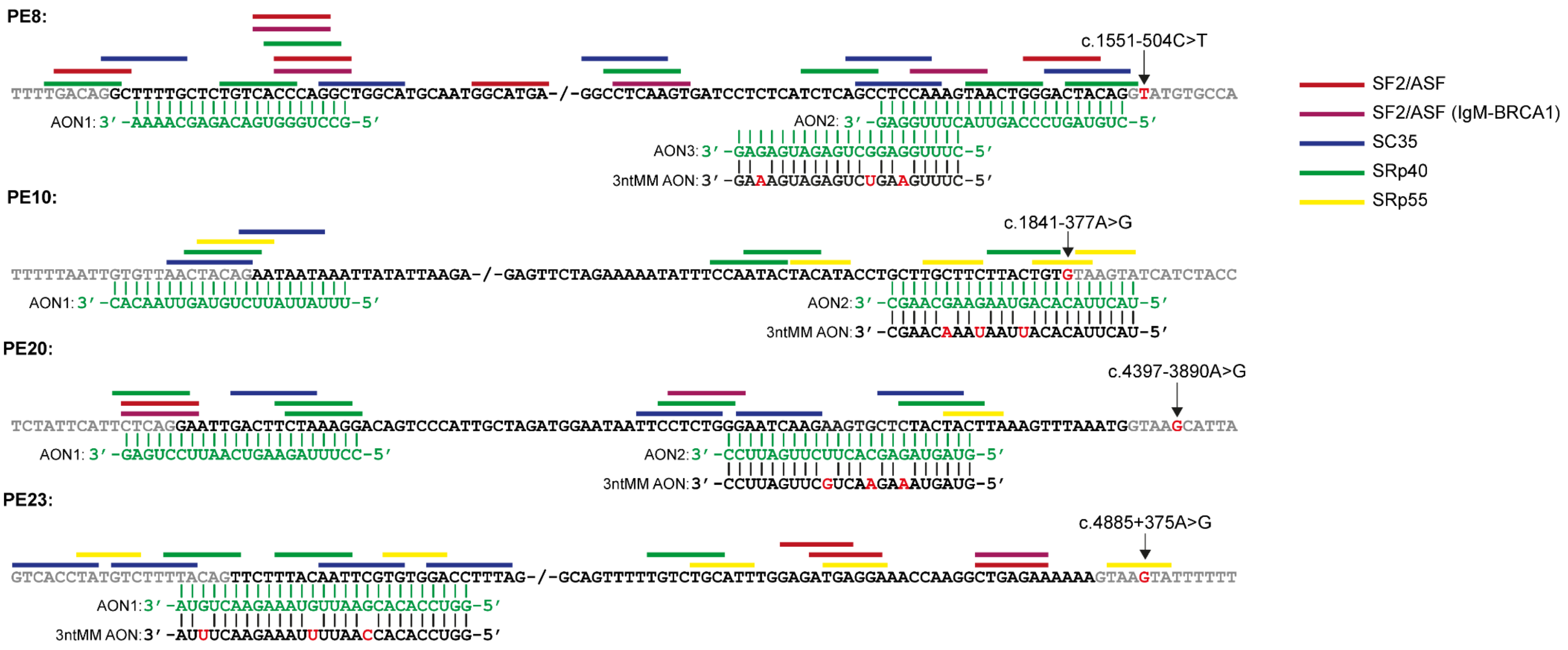

Figure S2: Position of antisense oligonucleotides (AONs) and 3 nucleotide mismatch AONs (3ntMM AONs) relative to their targets.
AONs were designed complementary to the splice sites or exonic splice enhancers of each specific pseudoexon (PE). The sequence of each AON is shown in green, 3ntMM AONs are in black with mismatches in red. The PE sequences that are incorporated in the mature *USH2A* transcript as a consequence of the identified deep-intronic variants are depicted in black, intronic sequences are in grey and the identified genetic variants are indicated in red. The bars above the sequences represent different types of putative exonic splice enhancers based on predictions from the ESEfinder tool[1] accesssed from Alamut Visual Plus v1.4.

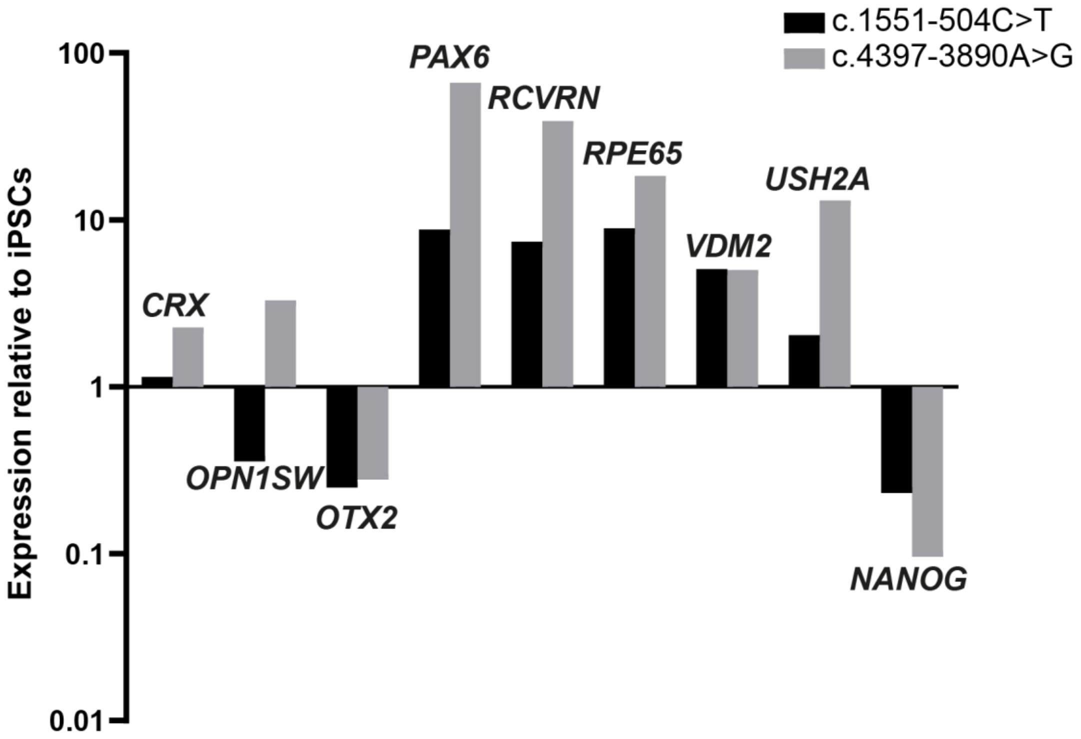

Figure S3: Differentiation efficiency of patient-derived photoreceptor precursor cells (PPCs). Expression of several neuronal progenitor, photoreceptor and retinal pigment epithelium markers (*CRX, OPN1SW, OTX2, PAX6, RCVRN, RPE65, VDM2*) as well as expression of *USH2A* and a pluripotency marker (*NANOG*) were determined with RT-qPCR in induced pluripotent stem cells (iPSCs) and compared PPCs after 30 days of differentiation. Expression was normalized against *GUSB* and compared to the corresponding iPSC line at the start of differentiation (day 0) using "delta delta Ct". As expected, pluripotency markers decreased while most of the other markers showed an increased. These data indicate that the differentiation of iPSCs towards PPCs was successful for both cell lines.

Table S1: Pre-screening methods of all samples

Study ID	Causal gene	Status of proband	Pre-screening
arRP1	*USH2A*	Possibly solved	Molecular inversion probe based sequencing
arRP2	-	Unsolved	Molecular inversion probe based sequencing
arRP3	-	Unsolved	Molecular inversion probe based sequencing
arRP4	*PQLC2*	Solved	Molecular inversion probe based sequencing
arRP5	-	Unsolved	Whole exome sequencing
arRP6	*EYS*	Possibly solved	Whole exome sequencing
arRP7	-	Unsolved	Whole exome sequencing
arRP8	*USH2A*	Possibly solved	Whole exome sequencing
arRP9	-	Unsolved	Whole exome sequencing
arRP10	*USH2A*	Possibly solved	Whole exome sequencing
arRP11	*USH2A*	Solved	Target 5000 sequencing
arRP12	-	Unsolved	Target 5000 sequencing
arRP13	-	Unsolved	Target 5000 sequencing
arRP14	-	Unsolved	Target 5000 sequencing
arRP15	-	Unsolved	Whole exome sequencing
arRP16	-	Unsolved	Whole exome sequencing
arRP17	-	Unsolved	Targeted sequencing
arRP18	-	Unsolved	Target 5000 sequencing
arRP19	*USH2A*	Solved	Molecular inversion probe based sequencing
arRP20	*USH2A*	Solved	Molecular inversion probe based sequencing
arRP21	*USH2A*	Possibly solved	Molecular inversion probe based sequencing
arRP22	*USH2A*	Solved	Molecular inversion probe based sequencing
arRP23	-	Unsolved	Molecular inversion probe based sequencing
arRP24	*USH2A*	Possibly solved	Molecular inversion probe based sequencing
arRP25	-	Unsolved	Molecular inversion probe based sequencing
arRP26	*USH2A*	Solved	Molecular inversion probe based sequencing
arRP27	-	Unsolved	Molecular inversion probe based sequencing
arRP28	-	Unsolved	Cegat panel sequencing
arRP29	*PROM1*	Possibly solved	Cegat panel sequencing
arRP30	-	Unsolved	Cegat panel sequencing
arRP31	-	Unsolved	Cegat panel sequencing
arRP32	-	Unsolved	Molecular inversion probe based sequencing
arRP33	-	Unsolved	Sanger sequencing
arRP34	*USH2A*	Solved	Micro array and Sanger sequencing
arRP35	-	Unsolved	Whole exome sequencing
arRP36	*USH2A*	Solved	Whole exome sequencing
arRP37	*RPE65*	Solved	Whole exome sequencing
arRP38	-	Unsolved	Whole exome sequencing
arRP39	-	Unsolved	Whole exome sequencing
arRP40	-	Unsolved	Whole exome sequencing
arRP41	*USH2A*	Solved	Whole exome sequencing

arRP42	*USH2A*	Solved	Whole exome sequencing
arRP43	-	Unsolved	Whole exome sequencing
arRP44	-	Unsolved	Whole exome sequencing
arRP45	-	Unsolved	Whole exome sequencing
arRP46	-	Unsolved	Whole exome sequencing
arRP47	-	Unsolved	Whole exome sequencing
CRD1	-	Unsolved	Molecular inversion probe based sequencing
DFNB1	-	Unsolved	Targeted sequencing
DFNB2	*USH2A*	Solved	Targeted sequencing
DFNB3	-	Unsolved	Targeted sequencing
USH1	-	Unsolved	Molecular inversion probe based sequencing and multiplex ligation-dependent probe amplification
USH2	-	Unsolved	Whole exome sequencing
USH3	-	Unsolved	Whole exome sequencing
USH4	-	Unsolved	Molecular inversion probe based sequencing and whole exome sequencing
USH5	*USH2A*	Solved	Targeted sequencing
USH6	-	Unsolved	Targeted sequencing
USH7	*USH2A*	Solved	Targeted sequencing
USH8	*USH2A*	Solved	Targeted sequencing
USH9	-	Unsolved	Targeted sequencing
USH10	*USH2A*	Solved	Molecular inversion probe based sequencing
USH11	*PEX6*	Solved	Molecular inversion probe based sequencing
USH12	*USH2A*	Solved	Targeted resequencing panel of 10 USH genes
USH13	*USH2A*	Solved	Targeted resequencing panel of 10 USH genes
USH14	-	Unsolved	Whole exome sequencing
USH15	-	Unsolved	Whole exome sequencing
USH16	*USH2A*	Solved	Whole exome sequencing
USH17	*USH2A*	Solved	Target 5000 sequencing
USH18	-	Unsolved	Target 5000 sequencing
USH19	*USH2A*	Solved	Target 5000 sequencing
USH20	*ARSG*	Solved	Target 5000 sequencing
USH21	-	Unsolved	Target 5000 sequencing
USH22	*USH2A*	Solved	Target 5000 sequencing
USH23	*USH2A*	Possibly solved	Target 5000 sequencing
USH24	*USH2A*	Solved	Targeted sequencing of 13 USH genes and multiplex ligation-dependent probe amplification
USH25	*MYO7A*	Solved	Whole exome sequencing
USH26	*USH2A*	Solved	Whole exome sequencing
USH27	-	Unsolved	Targeted sequencing
USH28	*USH2A*	Solved	Treatrush sequencing
USH29	*USH2A*	Possibly solved	Treatrush sequencing
USH30	-	Unsolved	Treatrush sequencing

USH31	*USH2A*	Solved	Treatrush sequencing
USH32	*USH2A*	Possibly solved	Cegat panel sequencing
USH33	*USH2A*	Solved	Treatrush sequencing
USH34	*USH2A*	Solved	Treatrush sequencing
USH35	*ARSG*	Solved	Cegat panel sequencing
USH36	-	Unsolved	Cegat panel sequencing
USH37	*USH2A*	Solved	Sanger sequencing
USH38	-	Unsolved	Whole exome sequencing
USH39	*USH2A*	Solved	Micro array and Sanger sequencing
USH40	-	Unsolved	Whole exome sequencing
USH41	*USH2A*	Solved	Micro array and Sanger sequencing
USH42	*USH2A*	Solved	Molecular inversion probe based sequencing
USH43	-	Unsolved	Whole exome sequencing
USH44	*USH2A*	Solved	Whole exome sequencing
USH45	-	Unsolved	Whole exome sequencing
USH46	*USH2A*	Possibly solved	Whole exome sequencing
USH47	-	Unsolved	Whole exome sequencing
USH48	-	Unsolved	Whole exome sequencing
USH49	*USH2A*	Possibly solved	Targeted sequencing and multiplex ligation-dependent probe amplification

Table S2: All genes associated with Usher(-like) syndrome and autosomal recessive retinitis pigmentosa that were assessed in genome sequencing data of 100 cases.

**Autosomal recessive retinitis pigmentosa**	*ABCA4*	*AGBL5*	*AHR*	*ARHGEF18*	*ARL2BP*
	ARL6	*BBS1*	*BBS2*	*BEST1*	*C2orf71*
	C8orf37	*CERKL*	*CLCC1*	*CLRN1*	*CNGA1*
	CNGB1	*CRB1*	*CYP4V2*	*DHDDS*	*DHX38*
	EMC1	*EYS*	*FAM161A*	*GPR125 (ADGRA3)*	*HGSNAT*
	IDH3B	*IFT140*	*IFT172*	*IMPG2*	*KIAA1549*
	KIZ	*LRAT*	*MAK*	*MERTK*	*MVK*
	NEK2	*NEUROD1*	*NR2E3*	*NRL*	*PDE6A*
	PDE6B	*PDE6G*	*POMGNT1*	*PRCD*	*PROM1*
	RBP3	*REEP6*	*RGR*	*RHO*	*RLBP1*
	RP1	*RP1L1*	*RPE65*	*SAG*	*SAMD11*
	SLC7A14	*SPATA7*	*TRNT1*	*TTC8*	*TULP1*
	ZNF408	*ZNF513*			
**Usher syndrome**	*ABHD12*	*ADGRV1*	*ARSG*	*CDH23*	*CEP250*
	CEP78	*CIB2*	*CLRN1*	*ESPN*	*HARS1*
	MYO7A	*PCDH15*	*PEX1*	*PEX6*	*USH1C*
	USH1G	*WHRN*			
**Retinal modifier of Usher syndrome**	*PDZD7*				

Table S3: Sequences of primers used to generate constructs for the minigene splice assays.

Target variant (NM_206933.2)	Forward primer (5'>3')	Reverse primer (5'>3')
c.1551-504C>T	GGGGACAAGTTTGTACAAAAAAGCAGGCTTCtcccttcaccaccaacttcc	GGGGACCACTTTGTACAAGAAAGCTGGGTGagtgccatgctatccaaacac
c.1644+7453A>G	GGGGACAAGTTTGTACAAAAAAGCAGGCTTCtaagaggccccaatgtgtgt	GGGGACCACTTTGTACAAGAAAGCTGGGTGtggggcggaagagttaacat
c.1841-377A>G	GGGGACAAGTTTGTACAAAAAAGCAGGCTTCggagatcgagaccatgctgg	GGGGACCACTTTGTACAAGAAAGCTGGGTGatgcaaaggaccaccgaact
c.2303G>A	GGGGACAAGTTTGTACAAAAAAGCAGGCTTCtgcattcttttcaaaccagatgc	GGGGACCACTTTGTACAAGAAAGCTGGGTGtttcaggggacatagggtgg
c.4396+6885T>C	GGGGACAAGTTTGTACAAAAAAGCAGGCTTCacatcaatggaaacgagtgacc	GGGGACCACTTTGTACAAGAAAGCTGGGTGtggaaaggagaaaatgtaggctc
c.4397-3890A>G	GGGGACAAGTTTGTACAAAAAAGCAGGCTTCttctggcctagcagtgtttg	GGGGACCACTTTGTACAAGAAAGCTGGGTGcagggaagcaatggagaacc
c.4714C>T	GGGGACAAGTTTGTACAAAAAAGCAGGCTTCacaattcccgccaaatccttc	GGGGACCACTTTGTACAAGAAAGCTGGGTGtcttcctttccctctggctg
c.4885+375A>G	GGGGACAAGTTTGTACAAAAAAGCAGGCTTCtgtcatacatgttgtcgagctc	GGGGACCACTTTGTACAAGAAAGCTGGGTGaagaggaggagaaggtgcac
c.5573-19A>G	GGGGACAAGTTTGTACAAAAAAGCAGGCTTCtcaccaccatccctctgaag	GGGGACCACTTTGTACAAGAAAGCTGGGTGcaggccacaagatcgagttg
c.5775A>T	GGGGACAAGTTTGTACAAAAAAGCAGGCTTCtcaccaccatccctctgaag	GGGGACCACTTTGTACAAGAAAGCTGGGTGcaggccacaagatcgagttg
c.6806-7599C>G	GGGGACAAGTTTGTACAAAAAAGCAGGCTTCcacatccctgcctcattcac	GGGGACCACTTTGTACAAGAAAGCTGGGTGcactgtctttgctacatcccag
c.8710G>A	GGGGACAAGTTTGTACAAAAAAGCAGGCTTCccatgccacaccacctttg	GGGGACCACTTTGTACAAGAAAGCTGGGTGgctgtaaccaaattcaaggctg
c.9258G>T	GGGGACAAGTTTGTACAAAAAAGCAGGCTTCccaagcaagtgttccaggtc	GGGGACCACTTTGTACAAGAAAGCTGGGTGgatgggttgttaggtgcagc
c.9259-9T>A	GGGGACAAGTTTGTACAAAAAAGCAGGCTTCcagaatgtaggcccttgatagtg	GGGGACCACTTTGTACAAGAAAGCTGGGTGtctcctgagcttgtgatccg
c.9959-3C>G	GGGGACAAGTTTGTACAAAAAAGCAGGCTTCaaacaattcaggaccccagg	GGGGACCACTTTGTACAAGAAAGCTGGGTGgcttgaagtgcatttggagc
c.12343C>T	GGGGACAAGTTTGTACAAAAAAGCAGGCTTCagatatggcagtccccttcc	GGGGACCACTTTGTACAAGAAAGCTGGGTGtgttccctgtattcactgtactc
c.14134-5T>C	GGGGACAAGTTTGTACAAAAAAGCAGGCTTCtgctagctccttacttccctg	GGGGACCACTTTGTACAAGAAAGCTGGGTGtgcagaaatgatggtggttcc
c.14583-26A>G	GGGGACAAGTTTGTACAAAAAAGCAGGCTTCcgttaacacgtttgaggcac	GGGGACCACTTTGTACAAGAAAGCTGGGTGgccacgggaaatgcaaatac
c.14664G>A	GGGGACAAGTTTGTACAAAAAAGCAGGCTTCcgttaacacgtttgaggcac	GGGGACCACTTTGTACAAGAAAGCTGGGTGgccacgggaaatgcaaatac
c.14753C>T	GGGGACAAGTTTGTACAAAAAAGCAGGCTTCcgttaacacgtttgaggcac	GGGGACCACTTTGTACAAGAAAGCTGGGTGgccacgggaaatgcaaatac
c.14791+5G>T	GGGGACAAGTTTGTACAAAAAAGCAGGCTTCccagcacgatgaagactcttg	GGGGACCACTTTGTACAAGAAAGCTGGGTGtctgggtggaggggtataca
RHO exons 3 to 5 (RT-PCR)	cggaggtcaacaacgagtct	aggtgtaggggatgggagac
GAPDH control (RT-PCR)	ctgcaccaccaactgcttag	agctcagggatgaccttgc

The sequence of the Gateway® attB site is depicted in upper case, the *USH2A*-specific sequence in lower case letters.

Table S4: Sequences of primers used in qPCR.

Target	Remark	Forward primer (5'>3')	Reverse primer (5'>3')
GUSB	Housekeeping gene	AGAGTGGTGCTGAGGATTGG	CCCTCATGCTCTAGCGTGTC
CRX		CCCCAGTGTGGATCTGATG	CAAACAGTGCCTCCAGCTC
NANOG		CCTGTGATTTGTGGGCCTG	CAGTCTCCGTGTGAGGCAT
OPN1SW		TTCTTCTCCAAGAGTGCTTGC	CCTTCCCACACACCATCTTC
OTX2		TATCTTAAGCAACCGCCTTACG	GGAGGGGTGCAGCAAGTC
PAX6		GCTGCAAAGAAATAGAACATCC	TTGGCTGCTAGTCTTTCTCG
RCVRN		ACACCAAGTTCTCGGAGGAG	ACTTGGCGTAGATGCTCTGG
RPE65		TTACTACGCTTGCACAGAGACC	GCCCCATTGACAGAGACATAG
VDM2		TCAGTGTGGACACCTGTATGC	AAGCTGTACACCGCCACAG
USH2A Exon 8-9	Specific for wildtype transcript	ACAACTGAGACTGCTGTTAACC	GACCATGGCACTGACATCTC
USH2A exon 8-PE8	Specific for transcript with PE	ACAACTGAGACTGCTGTTAACC	CAGAGCAAAAGCCTCCCAC
USH2A exon 12-13	Reference for expression levels	GCAAAGCAAACGTTATTGGGCT	TACACTGGCAGGGCTCACAT
USH2A exon 20-21	Specific for wildtype transcript	ACTTTAGCAGCAGCACCAGC	GGAGAGGGTCCATTCAGTTC
USH2A PE20-exon 21	Specific for transcript with PE	GGACAGTCCCATTGCTAGATG	AGTTCTTGGGATTTAGCAGTGTG

PE: pseudoexon

Table S5: All *USH2A* variants that met our variant inclusion criteria in 100 cases.

See separate excel sheet

Table S6: All *USH2A* variants with a predicted effect on splicing.

See separate excel sheet

Table S7: Overview of observed effects on pre-mRNA splicing and consequences on protein level of 21 variants that were tested with minigene splice assays.

Variant	Effect on splicing	Protein effect
**Deep-intronic variants**		
c.1551-504C>T	PE inclusion (Δ118 nt)	p.Arg517_Cys518ins*13
c.1644+7453A>G	No effect	p.=
c.1841-377A>G	1. PE inclusion (Δ94 nt), 2. No effect	p.[Gly614Aspfs*9,=]
c.4396+6885T>C	No effect	p.=
c.4397-3890A>G	PE inclusion (Δ87 nt)	p.Ala1465_Ala1466ins*5
c.4885+375A>G[2]	PE inclusion (Δ130 nt)	p.Ser1629Valfs*52
c.6806-7599C>G	No effect	p.=
**Non-canonical splice site variants**		
c.5775A>T	Exon 28 skipping (Δ204 nt)	p.Gly1858_Thr1925del
c.9258G>T	1. Partial exon 46 skipping (Δ153 nt), 2. Partial exon 46 skipping (Δ116 nt)	p.[Arg3037_Val3087del,Val3049*]
c.9259-9T>A	Inclusion of last 7 nt of intron 46 to exon 47	p.Val3087Phefs*4
c.9959-3C>G	1. Exon 51 skipping (Δ224 nt), 2. Partial exon 51 skipping (Δ57 nt)	p.[Met3321Asnfs*22,=,Gly3320_Ser3338del]
c.14134-5T>C	1. No effect, 2. Intron 64 inclusion (Δ47 nt)	p.[=,Val4712Profs*2]
c.14791+5G>T	Exon 67 skipping (Δ209 nt)	p.Tyr4862Alafs*22
**Exonic variants**		
c.2303G>A	1. No effect, 2. Partial skipping of center exon 13 (Δ398 nt), 3. Partial exon 13 skipping (Δ513 nt)	p.[Cys768Tyr,Cys766Tyrfs*3,Glu767_Gly937del]
c.4714C>T	No effect	p.(Leu1572Phe)
c.8710G>A	No effect	p.(Val2904Ile)
c.12343C>T	No effect	p.(Arg4115Cys)
c.14664G>A	No effect	p.(Thr4888=)
c.14753C>T	No effect	p.(Thr4918Met)
**Branchpoint variants**		
c.5573-19A>G	Exon 28 skipping (Δ204 nt)	p.Gly1858_Thr1925del
c.14583-26A>G	1. No effect, 2. Exon 67 skipping (Δ209 nt)	p.[=,Tyr4862Alafs*22]

nt: nucleotide; PE: pseudoexon

Table S8: Characteristics of all oligonucleotides designed in this study

Oligonucleotide	Sequence (3’>5’)	Target variant	Coordinates of pseudoexon (hg19)	Length (nt)	GC-content (%)
AON1 USH2A PE8	GCCUGGGUGACAGAGCAAAA	c.1551-504C>T (p.Arg517_Cys518ins*13)	chr1:216495824-216495941	20	55
AON2 USH2A PE8^	CUGUAGUCCCAGUUACUUUGGAG			23	48
AON3 USH2A PE8	CUUUGGAGGCUGAGAUGAGAG			21	52
SON USH2A PE8	CUCUCAUCUCAGCCUCCAAAG			21	52
AON3 3ntMM USH2A PE8	CUUUGaAGuCUGAGAUGAaAG			21	38
AON1 USH2A PE10	UUUAUUAUUCUGUAGUUAACAC	c.1841-377A>G (p.[Thr613_Gly614ins*9,=])	chr1:216463130-216463223	22	23
AON2 USH2A PE10	UACUUACACAGUAAGAAGCAAGC			23	39
AON2 3ntMM USH2A PE10	UACUUACACAuUAAuAAaCAAGC			23	26
SON USH2A PE10*	GUAGUAAAGAACUGCUUGAUUCC			23	39
AON1 USH2A PE20	CCUUUAGAAGUCAAUUCCUGAG	c.4397-3890A>G (p.Ala1465_Ala1466ins*5)	chr1:216352719-216352805	22	41
AON2 USH2A PE20	GUAGUAGAGCACUUCUUGAUUCC			23	43
SON USH2A PE20	GGAAUCAAGAAGUGCUCUACUAC			23	43
AON2 3ntMM USH2A PE20*	GUAGUAaAGaACUgCUUGAUUCC			23	39
AON1 USH2A PE23	GGUCCACACGAAUUGUAAAGAACUGUA	c.4885+375A>G[2] (p.Ser1629Valfs*52)	chr1:216261985-216262114	27	41
SON USH2A PE23*	GUAGUAAAGAACUGCUUGAUUCC			27	33
AON1 3ntMM USH2A PE23	GGUCCACACcAAUUuUAAAGAACUuUA			23	39

Oligonucleotides labeled with an asterisk are the same. For 3 nucleotide mismatch antisense oligonucleotides (3ntMM AONs), lowercase nucleotides are mismatches. PE: pseudoexon; SON: sense oligonucleotide; ^: many off-target binding regions predicted for this oligonucleotide

Supplemental references

1. Cartegni, L., Wang, J., Zhu, Z., Zhang, M. Q. & Krainer, A. R. ESEfinder: A web resource to identify exonic splicing enhancers. Nucleic Acids Res 31, 3568-3571, doi:10.1093/nar/gkg616 (2003).
2. Mansard, L. et al. The Study of a 231 French Patient Cohort Significantly Extends the Mutational Spectrum of the Two Major Usher Genes MYO7A and USH2A. Int J Mol Sci 22, doi:10.3390/ijms222413294 (2021).
3. Liquori, A. et al. Whole USH2A Gene Sequencing Identifies Several New Deep Intronic Mutations. Hum Mutat 37, 184-193, doi:10.1002/humu.22926 (2016).
4. Vaché, C. *et al.* Usher syndrome type 2 caused by activation of an USH2A pseudoexon: implications for diagnosis and therapy. *Hum Mutat* **33**, 104-108, doi:10.1002/humu.21634 (2012).
5. Lenassi, E. et al. A detailed clinical and molecular survey of subjects with nonsyndromic USH2A retinopathy reveals an allelic hierarchy of disease-causing variants. Eur J Hum Genet 23, 1318-1327, doi:10.1038/ejhg.2014.283 (2015).
